# The chromosome‐scale reference genome of safflower (*Carthamus tinctorius*) provides insights into linoleic acid and flavonoid biosynthesis

**DOI:** 10.1111/pbi.13586

**Published:** 2021-04-08

**Authors:** Zhihua Wu, Hong Liu, Wei Zhan, Zhichao Yu, Erdai Qin, Shuo Liu, Tiange Yang, Niyan Xiang, Dave Kudrna, Yan Chen, Seunghee Lee, Gang Li, Rod A. Wing, Jiao Liu, Hairong Xiong, Chunjiao Xia, Yongzhong Xing, Jianwei Zhang, Rui Qin

**Affiliations:** ^1^ Hubei Provincial Key Laboratory for Protection and Application of Special Plant Germplasm in Wuling Area of China Key Laboratory of State Ethnic Affairs Commission for Biological Technology College of Life Sciences South‐Central University for Nationalities Wuhan China; ^2^ National Key Laboratory of Crop Genetic Improvement Huazhong Agricultural University Wuhan China; ^3^ Arizona Genomics Institute School of Plant Sciences University of Arizona Tucson AZ USA; ^4^ Center for Desert Agriculture, Biological and Environmental Sciences and Engineering Division (BESE) King Abdullah University of Science and Technology (KAUST) Thuwal Saudi Arabia

**Keywords:** safflower, linoleic acid, flavonoid, genome, evolution, transcriptome

## Abstract

Safflower (*Carthamus tinctorius* L.), a member of the Asteraceae, is a popular crop due to its high linoleic acid (LA) and flavonoid (such as hydroxysafflor yellow A) contents. Here, we report the first high‐quality genome assembly (contig N50 of 21.23 Mb) for the 12 pseudochromosomes of safflower using single‐molecule real‐time sequencing, Hi‐C mapping technologies and a genetic linkage map. Phyloge

nomic analysis showed that safflower diverged from artichoke (*Cynara cardunculus*) and sunflower (*Helianthus* *annuus*) approximately 30.7 and 60.5 million years ago, respectively. Comparative genomic analyses revealed that uniquely expanded gene families in safflower were enriched for those predicted to be involved in lipid metabolism and transport and abscisic acid signalling. Notably, the fatty acid desaturase 2 (FAD2) and chalcone synthase (CHS) families, which function in the LA and flavonoid biosynthesis pathways, respectively, were expanded via tandem duplications in safflower. *CarFAD2‐12* was specifically expressed in seeds and was vital for high‐LA content in seeds, while tandemly duplicated *CarFAD2* genes were up‐regulated in ovaries compared to *CarFAD2‐12*, which indicates regulatory divergence of *FAD2* in seeds and ovaries. *CarCHS1*, *CarCHS4* and tandem‐duplicated *CarCHS5*˜*CarCHS6*, which were up‐regulated compared to other *CarCHS* members at early stages, contribute to the accumulation of major flavonoids in flowers. In addition, our data reveal multiple alternative splicing events in gene families related to fatty acid and flavonoid biosynthesis. Together, these results provide a high‐quality reference genome and evolutionary insights into the molecular basis of fatty acid and flavonoid biosynthesis in safflower.

## Introduction

Safflower (*Carthamus tinctorius* L., 2*n* = 2*x* = 24) is a member of the largest family of flowering plants, the Asteraceae, which contains approximately 24 000–35 000 species. Asteraceae species, including sweet wormwood (*Artemisia annua*), sunflower (*Helianthus* *annuus*), lettuce (*Lactuca sativa*) and chrysanthemums (*Chrysanthemum nankingense*), are medicinally, ornamentally or economically valuable (Barreda *et al.,* 2015). One of the oldest annual oil seed crops in human history, safflower is believed to have been domesticated in the Fertile Crescent region over 4000 years ago (Chapman and Burke, 2007) and has been widely cultivated in Asia, Europe, Australia and the Americas for its agronomic traits (Bowers *et al.,* 2016). Safflower has a high proportion of polyunsaturated fatty acids in its seeds, mainly in the form of linoleic acid (LA) or oleic acid (OA), which is essential for human health (Knutzon *et al.,* 1992). Safflower flowers have also been explored as a source of yellow and red dyes as a medicine and natural food colourant due to their high flavonoid contents. The major bioactive flavonoid hydroxysafflor yellow A (HSYA) is uniquely isolated from safflower petals and has a variety of potent biological functions, such as antioxidative and myocardial and cerebral protective effects (Zhu *et al.,* 2003). As a traditional Chinese medicine, dried safflower flowers have been widely used to improve cerebral blood flow and to treat various ailments, such as gynaecological, cerebrovascular, and cardiovascular diseases, hypertension and coronary heart disease (Lou and Liu, 1956).

For years, efforts have been made to understand the molecular mechanisms underlying the two most important traits in safflower, fatty acid composition (Golkar *et al.,* 2011) and flavonoid biosynthesis, via genetic analyses (Li *et al.,* 2010). Many flavonoid biosynthesis genes have been cloned in safflower, including those encoding chalcone synthases (CHSs), UDP‐glucuronosyltransferases (UGTs), chalcone isomerases (CHIs) and flavanone 3‐hydroxylases (F3Hs; Chen *et al.,* 2020); however, the evolution and regulation of gene families involved the biosynthesis of flavonoids such as HSYA remain unclear at the genomic level. Until now, there has been only one whole‐genome sequencing effort for genetic mapping of safflower with short‐read sequencing (Bowers *et al.,* 2016). Elucidation of the molecular mechanisms related to fatty acid and flavonoid biosynthesis in safflower has been greatly hindered by the lack of a high‐quality reference genome sequence.

In this study, we used *de novo* assembly to prepare a chromosome‐level reference genome for ‘Anhui‐1’ safflower, a cultivar with high‐LA content, based on long‐read sequencing, Hi‐C chromatin contact maps and F_2_ genetic linkage groups; we then generated comprehensive transcriptome data from multiple tissues. Using 10 previously published genomes of Asteraceae and outgroup species, we performed an evolutionary analysis to assess the divergence of the safflower genome, as well as the genomic signatures of LA and flavonoid biosynthesis. Combined with measurement of the fatty acid and flavonoid contents, we carried out comparative analyses of multiple tissues and developmental stages to reveal the gene expression patterns, alternative splicing (AS) events, and gene clusters essential for LA and flavonoid biosynthesis. The genomic and transcriptomic resources provided here will be valuable not only for agronomy, medical research and the genetic improvement of safflower, but also for the study of evolution and speciation in the Asteraceae.

## Results

### Genome assembly and annotation

A genome survey using a *k*‐mer analysis (*k* = 17) revealed that the genome size and heterozygosity ratio of safflower cultivar ‘Anhui‐1’ with high‐LA content were approximately 1.17 Gb and 0.23%, respectively. Flow cytometry estimations also showed that the genome size of safflower was close to that of *Glycine max* (˜1.12 Gb), but with a lower heterozygosity (Figure S1). Based on the estimated genome size, a targeted genome coverage of 188× was obtained with about 207 Gb raw reads from 37 PacBio single‐molecule real‐time (SMRT) cells (Table S1).

The initial assembly size of the safflower genome was about 1.07 Gb, comprising a total of 368 contigs (ranging from 15 377 to 56 653 595 bp; N50 = 16.4 Mb), as *de novo* assembled using Canu (version 1.3; Koren *et al.,* 2017). To construct a chromosome‐scale reference genome, additional scaffold refinement was performed using 353 349 231 paired‐end reads from the Hi‐C sequencing. Given that the chromosome number of safflower is 2*n* = 24 (Raina *et al.,* 2005), the largest 12 superscaffolds were generated, comprising 213 contigs and about 1.06 Gb, reflecting a chromosome‐scale assembly representing 90.6% of the estimated genome size (1.17 Gb) or 99.1% of the initially assembled genome size generated using long‐read sequencing by PacBio Sequel (1.07 Gb; Figure S2; Table S2). The final 12 superscaffolds with 128 contigs (N50 = 21.23 Mb) were obtained by further correction of Falcon‐assembled contigs using the GPM pipeline (Zhang *et al.,* 2016). The chromosomes were assigned to 12 genetic linkage groups (Figure S3). A collinearity analysis (Bowers *et al.,* 2016) showed that the final assembled genome (the Safflower Reference Genome Sequence Version 1, SafflowerRS1) provides a high resolution at the chromosome level (Figure S4). A mapping ratio of ˜98.1% was reached by mapping all ˜35× paired‐end reads of Illumina‐generated sequences back to the scaffolds, while an average of ˜94.82% was achieved by the paired‐end reads of 87 RNA‐seq samples (Table S3), further demonstrating the quality and completeness of the assembly. The safflower genome assembly statistics are shown in Table 1.

**Table 1:** Statistics of the safflower genome assembly and gene annotation

Feature	SafflowerRS1
Genome assembly	
Estimated genome size (by *k*‐mer analysis) (Gb)	1.17
Number of contigs	128
Contig N50 (Mb)	21.23
Longest contig (Mb)	57.98
Assembly size (Gb) and % of genome*	1.06 (90.60%)
Repeat region % of assembly	60.13%
Gene annotation	
Predicted gene models	33 343
Number of transcripts	45 331
Average exons per gene	6.54
Mean exon length (bp)	269.59
Average CDS length (bp)	1265.89
Average intergenic region length (bp)	26 956.38
Number of long noncoding RNAs	10 646

\x90* Based on the estimated genome size of 1.17 Gb by *k*‐mer analysis.

**Figure 1:**
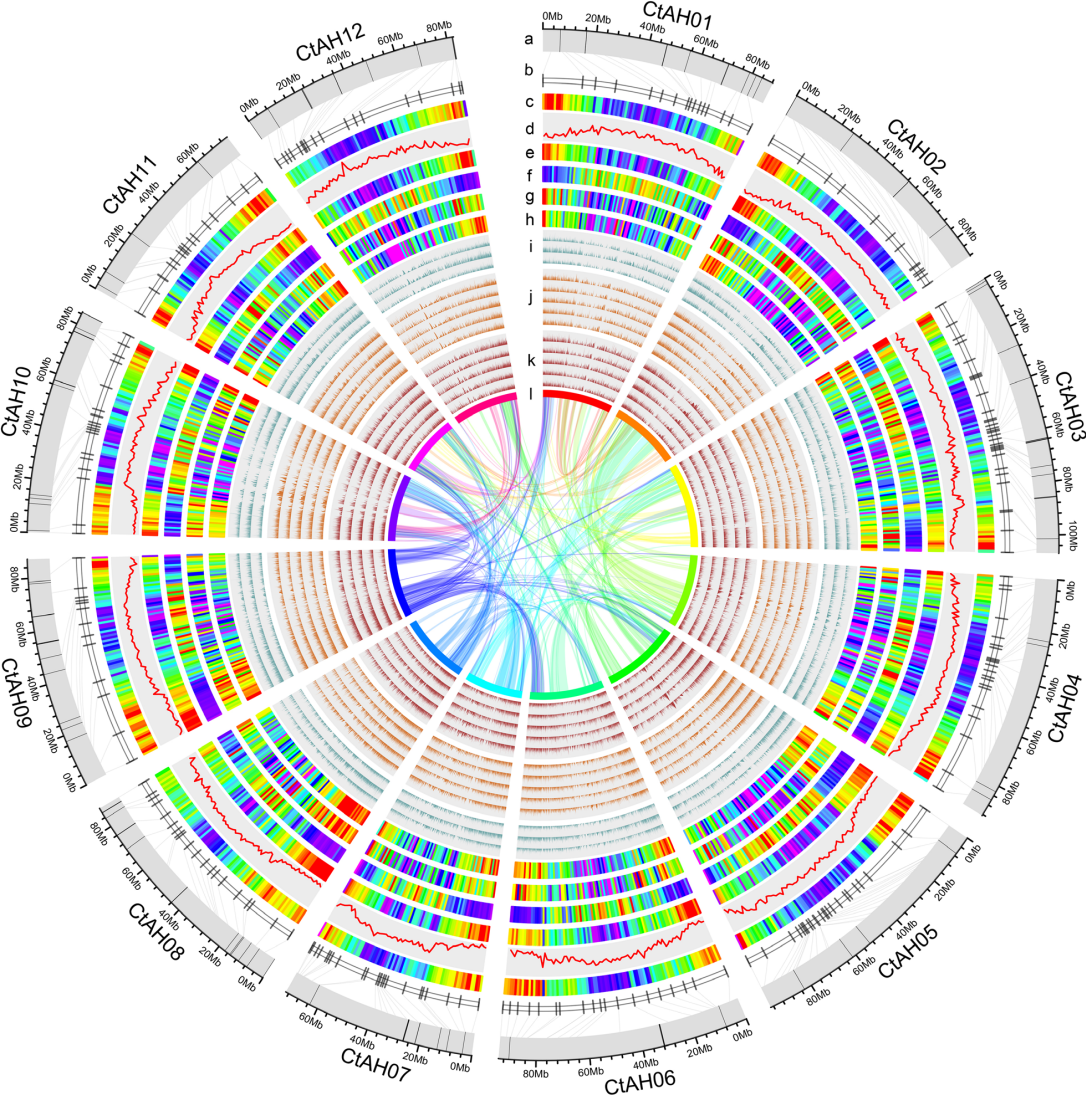
Landscape of the safflower genome and expression data. (a) Pseudochromosomes identified using Hi‐C. (b) The genetic linkage map from 248 simple sequence repeats (SSR). (c–h) The distribution of the SSR density, GC density, gene density, long terminal repeat retrotransposons density, long noncoding RNA density and differential alternative splicing events. (i–k) Expression of genes in different tissues. (i) Seeds at 0 days after flowering (DAF), 10 DAF and 20 DAF. (j) Flowers at initial flowering stage, middle bud stage, initial flowering stage, peak flowering stage and decayed flowering stage. (k) Cotyledons at 1 day after germination (DAG), 3 DAG, 5 DAG, 7 DAG and 10 DAG. (l) Syntenic blocks. The band width is proportional to the syntenic block size.

Benchmarking Universal Single‐Copy Ortholog (BUSCO; Simao *et al.,* 2015) analysis revealed 1306 (90.7%) complete orthologs against the database containing a total of 1440 highly conserved core proteins (Table S4). Repetitive elements comprise 60.13% of the genome. Of the repetitive elements, 27.13% were long terminal repeat (LTR) retrotransposons. LTR_Gypsy and LTR_Copia were the two most common of these, making up 45% and 36%, respectively, of the repetitive elements in the genome (Figure S5; Table S5). These transposable elements were distributed on the chromosomes with an inverse correlation to gene density (Figure 1).

Using about 439 Gb of RNA‐seq data from 87 samples (Table S3) and 254 353 PacBio transcripts in our study, we predicted 33 343 protein‐coding genes with average of 1266 bp length, and 94.78% of these were supported by PacBio long reads. Overall, 98.12% of safflower transcripts have functional descriptions, with matches to known proteins in InterPro (89.06%), NCBI Nr (86.89%), Arabidopsis (75.01%) and sunflower (83.63%). Alternative splicing (AS) is a regulatory process of gene expression by which multiple mRNA variants are produced from a single gene via different pre‐mRNA splicing events (Baralle and Giudice, 2017). AS transcripts were identified for 61.54% (20 518) of annotated genes, with intron retention being the most common AS type. The top three enriched processes of genes undergoing AS were actin filament‐based process, cytoskeleton organization and vesicle‐mediated transport. We also identified 10 646 long noncoding RNAs with different origins, with most originating from gene loci (68.02%) and sense strands (52.76%; Figure S6). An InterProScan Pfam analysis identified 4077 protein families containing 30 930 proteins and 14 098 genes with 2216 Gene Ontology (GO) terms, of which 41.47%, 13.45% and 45.08% of the genes were annotated in the biological process, cellular component and molecular function categories, respectively (Figure S7). Transcription factors (TFs), transcriptional regulators (TRs) and protein kinases (PKs) are three important classes of regulatory proteins associated with numerous aspects of plant growth and development, as well as biotic and abiotic stress responses (Zheng *et al.,* 2016). A total of 1755 TFs, 406 TRs and 1137 PKs were identified in the safflower genome, respectively. The total number (3298) of detected safflower regulatory proteins was greater than the number identified in the five other plant species analysed here as follows *Vitis vinifera* (grape, 2699), *Arabidopsis thaliana* (arabidopsis, 3214), *Coffea canephora* (robusta coffee, 2839), *Cynara cardunculus* (artichoke, 2868) and *Erigeron breviscapus* (dengzhanhua, 3273; Figure S8). In contrast to *A*. *thaliana*, *V. vinifera*, *C. canephora*, *L. sativa* (lettuce) and *C. nankingense* (chrysanthemums), more genes encoding FAR‐RED‐IMPAIRED RESPONSE1 (FAR1) family TFs were present in the safflower genome (209, 11.9% of the total safflower TFs) with the second most *FAR1* members detected in *A. annua* (208, 6.9%) and the third most in artichoke (57, 3.6%; Figure S9; Table S6).

### Comparative genomic and phylogenomic analyses

**Figure 2:**
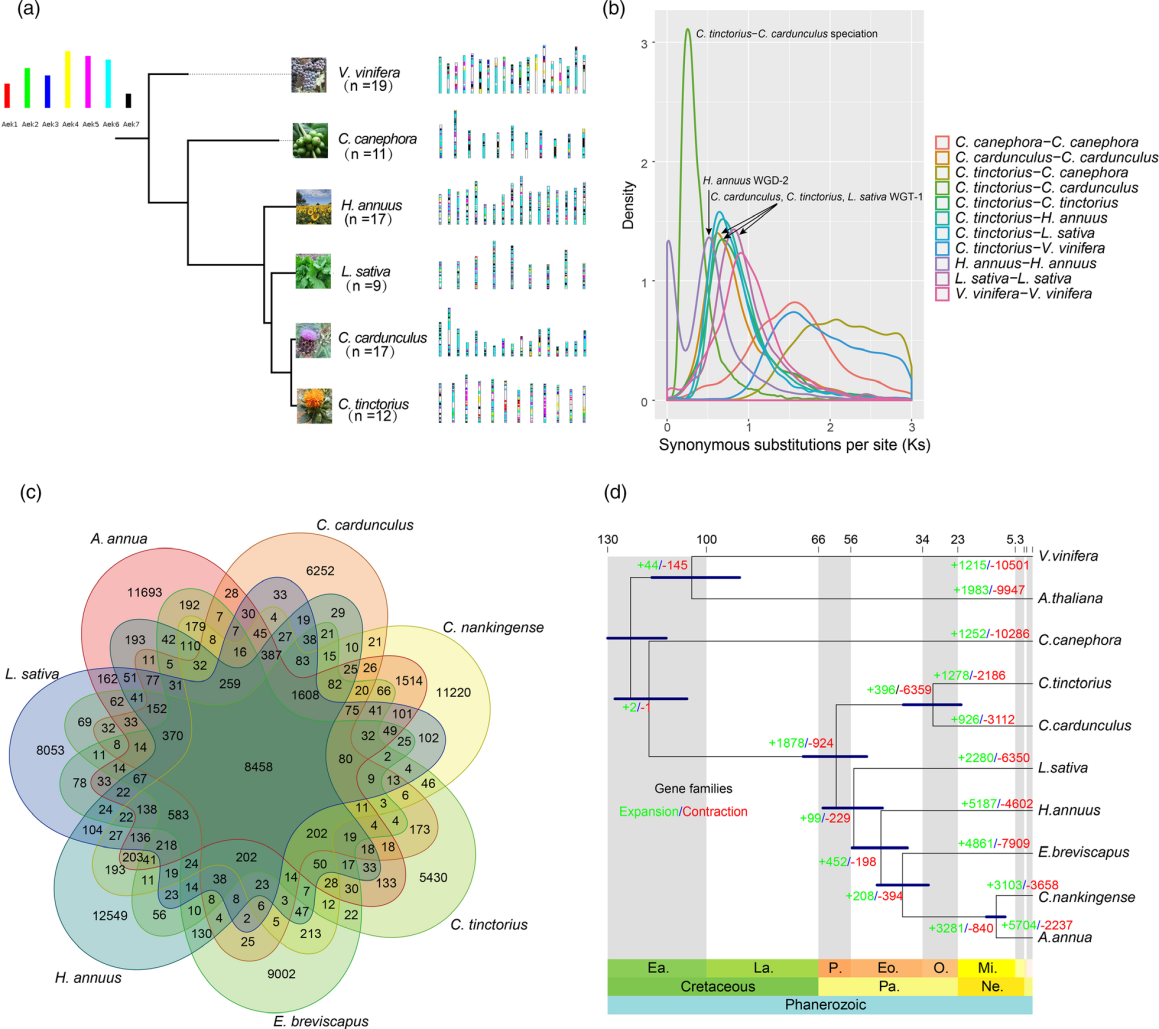
Comparative genomic analysis of safflower and other plant species. (a) Evolutionary scenario of the Asterids (*Coffea canephora*, *Helianthus* *annuus*, *Lactuca sativa*, *Cynara cardunculus* and *Carthamus tinctorius*) from the ancestral eudicot karyotype of seven (pre‐whole‐genome triplication event‐γ) protochromosomes. (b) Distribution of the synonymous substitution rates (*Ks*) for pairs of syntenic paralogs in safflower (*C. tinctorius*) and orthologs in the six other plants. (c) Number of gene families shared between safflower and six other species in the Asteraceae family. (d) Inferred phylogenetic tree with 385 single‐copy orthologs of ten species identified using OrthoFinder. The divergence times were estimated using MCMCTree and indicated by light blue bars at the internodes with a 95% highest posterior density. Ea, Early; La, Late; Pa, Paleogene; Ne, Neogene; P, Paleocene; Eo, Eocene; O, Oligocene; Mi, Miocene.

Whole‐genome duplication (WGD) is important for evolutionary innovations, as the resulting two copies of each gene have the potential to undergo functional diversification; for example, the species‐specific WGD event experienced by sunflower altered its genomic architecture and the regulation of flowering time (Badouin *et al.,* 2017). To study the evolution of the safflower genome, we performed a comparative analysis of six species with chromosome‐scale genomes to an ancestral eudicot karyotype (AEK) genome with seven protochromosomes (Murat *et al.,* 2017). Based on the AEK genome, we identified 6828 (25.9%) genes in *V*. *vinifera*, 14 893 (52.1%) in *C*. *canephora* (Asterid I), 18 669 (32.0%) in *H. annuus* (Asterid II), 16 220 (36.5%) in *L*. *sativa* (Asterid II), 15 691 (49.9%) in *C*. *cardunculus* (Asterid II) and 13 932 (34.9%) in safflower (Figure 2a). This suggested that after the γ‐WGT and Asterid II‐WGT events, these lineages underwent multiple chromosome rearrangements to varying degrees following their origin in the AEK genome. The types of duplication observed in the protein‐coding genes of safflower included WGD or segmental duplication (˜45.0%), tandem duplication (˜10.0%), singleton genes (˜13.8%), dispersed duplication (˜26.5%) and proximal duplication (˜4.7%). Similarly, high levels of WGD or segmental duplication were also observed in the closely related species artichoke, with ˜49.0% of genes displaying WGD or segmental duplication. This indicated that safflower and artichoke (which belong to the same subfamily, Carduoideae) may have shared a common WGD or segmental duplication event prior to their divergence from sunflower (Asteroideae) and lettuce (Cichorioideae; Figure S10). We further investigated and compared the genome duplication events during the evolution of safflower and the other Asterids. Our *Ks* and synteny analyses showed that safflower and artichoke shared a common WGD prior to their divergence (Figures 2b and S11).

To investigate the relationship between the gene families and the distinct traits of safflower, we compared the safflower genome and other eudicot genomes (Table S7). We observed that 5430 gene families containing 5658 genes were unique to safflower; these unique families were enriched for GO terms such as ‘GO:0006869, lipid transport’, ‘GO:0010876, lipid localization’, and ‘GO:0005992, trehalose biosynthetic process’ and Kyoto Encyclopedia of Genes and Genomes (KEGG) categories such as ‘ko00592, alpha‐linolenic acid metabolism’ and ‘ko00199, Cytochrome P450’ (Figures 2c and S12, Table S8). The enrichment of trehalose biosynthesis genes in this species is interesting because trehalose 6‐phosphate (the precursor of trehalose) positively regulates fatty acid biosynthesis in *Brassica napus* (Zhai *et al.,* 2018), and because trehalose has been reported to contribute to gamma‐linolenic acid accumulation in the fungus *Cunninghamella echinulata* (Li *et al.,* 2018).

A phylogenomic tree with the estimated divergence times for the 10 species was inferred using the maximum likelihood method with a joint coding sequence matrix from 385 single‐copy orthologs. The estimated divergence times indicated that safflower and artichoke diverged ˜30.7 million years ago (Mya; Oligocene), while safflower and sunflower diverged ˜60.5 Mya (Paleocene; Figure 2d). Safflower and artichoke diverged around when specific temperature conditions may have been established during the Oligocene (Barreda *et al.,* 2015). A total of 1278 expansions and 2186 contractions in the gene families were specific to safflower, among which 108 and 3 gene families showed rapid expansions and rapid contractions, respectively (Figure 2d; Table S9). GO and KEGG enrichment analyses of specific expanded gene families in safflower showed that ‘abscisic acid (ABA)‐activated signalling pathway’, ‘lipid biosynthesis proteins’, ‘alpha‐linolenic acid metabolism’ and ‘linoleic acid metabolism’ were enriched in the expanded gene families (Figure S13). ABA signalling is antagonistic to auxin and brassinosteroid (BR) signalling during plant development (Cai *et al.,* 2014). By contrast, the gene families involved in ‘response to auxin’ and ‘response to brassinosteroid’ contracted during safflower evolution (Figure S14).

### Genomic underpinning of LA biosynthesis during seed formation

To explore this fatty acid composition and the biosynthetic mechanism driving the high‐LA content in safflower, two cultivars were planted in the field in the autumn. One cultivar (‘HL’) had high‐LA content and low‐OA content; the other (‘LL’) had low‐LA and high‐OA content. The seeds of each cultivar were collected at 10 and 20 days after flowering (DAF) and analysed for their fatty acid composition and gene regulation (mRNAs, splicing isoforms and miRNAs) of genes related to the fatty acid biosynthesis pathway (Figures S15 and S16).

LA (18 : 2) and OA (18 : 1) were the main components of the total measured fatty acids in both ‘HL’ and ‘LL’ cultivars. The 10‐DAF (HL_DAF10) and 20‐DAF seed oils (HL_DAF20) of the ‘HL’ cultivar comprised 62.8% and 76.8% LA and 24.6% and 13.6% OA, respectively, while the ‘LL’ cultivar seeds contained only 1.9% and 0.5% LA but 66.4% and 90.6% OA at 10‐DAF (LL_DAF10) and 20‐DAF (LL_DAF20), respectively. Analysis of differentially expressed genes for HL_DAF10, HL_DAF20, LL_DAF10 and LL_DAF20 showed that 328 uniquely up‐regulated genes in HL_DAF20 were enriched in ‘ABA‐activated signalling pathway’ followed by ‘cellular response to lipid’, and 69 genes up‐regulated in HL_DAF20 but down‐regulated in LL_DAF20 were enriched in ‘biosynthesis of unsaturated fatty acids’, including multiple genes encoding FAD2 (fatty acid desaturase 2) enzymes, which are essential for LA biosynthesis (Figures S17 and S18). This suggested that high‐LA accumulation may be associated with activation of the ABA signalling pathway and biosynthesis of unsaturated fatty acids via FAD2s in the ‘HL’ cultivar.

**Figure 3:**
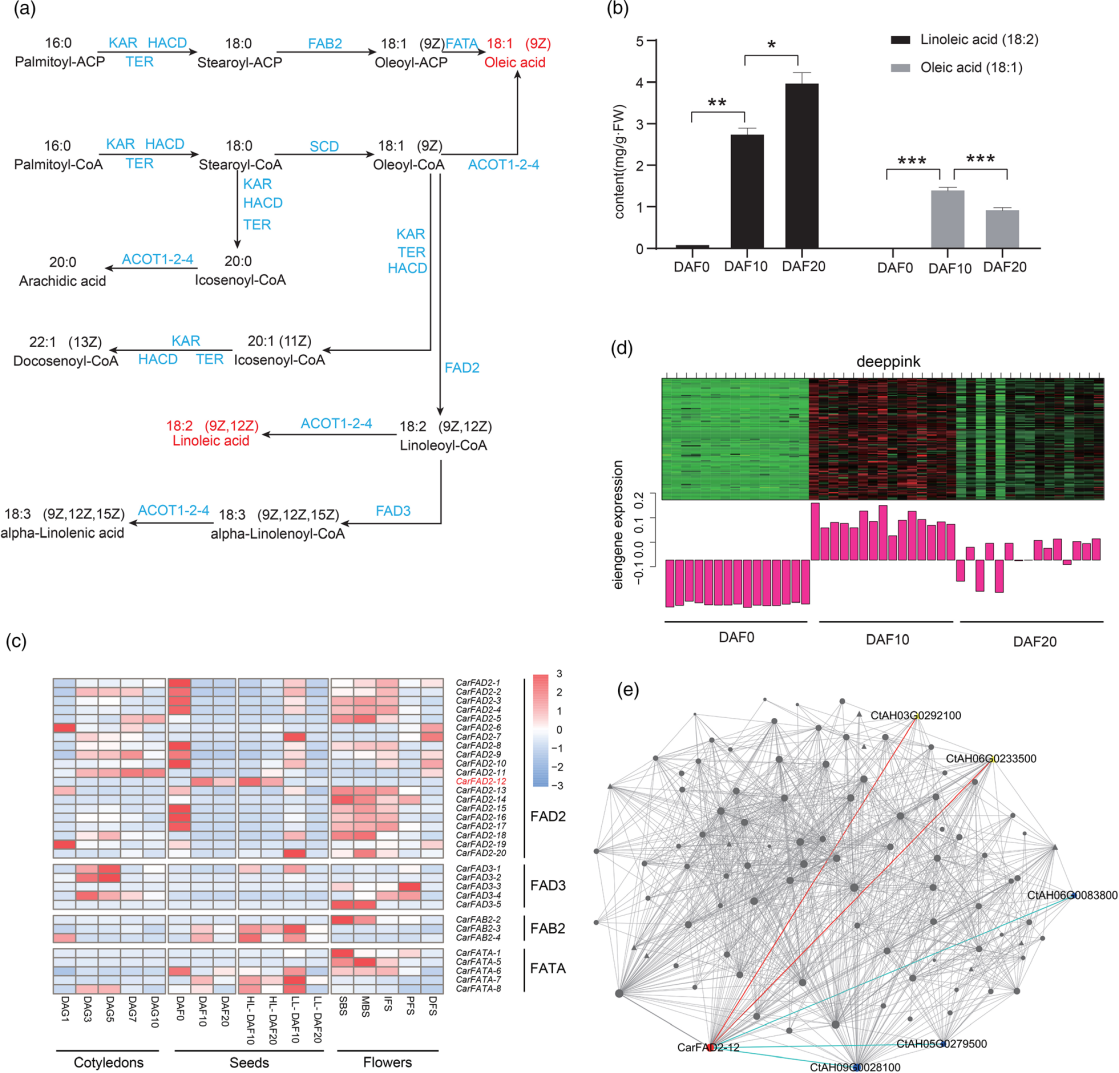
Analysis of gene families involved in linoleic acid (LA) and oleic acid (OA) biosynthesis in the ovaries and seeds of safflower. (a) Schematic representation of the unsaturated fatty acid biosynthesis pathway. In the biosynthesis pathway, LA and OA are marked in red, other intermediate compounds are in black, and enzymes are in blue. (b) Contents of LA and OA in ‘HL’ ovaries at 0 days after flowering (DAF), and ‘HL’ seeds at 10 and 20 DAF. Values are means ± SD from three independent experiments. Student’s *t*‐test: *P < 0.05; **P < 0.01; ***P < 0.001. (c) Expression patterns of key gene families (*FAD2*, *FAD3*, *FAB2* and *FATA*) involved in the biosynthesis of LA and OA in the cotyledons, seeds (including ovaries) and flowers. *CarFAD2‐12* is marked in red. The top right bar represents the normalized Z‐score values of FPKMs. (d) Gene expression pattern of genes in the ‘deeppink’ module, containing *CarFAD2‐12*, in the ovaries and seeds during seed development. € Genes coexpressed with *CarFAD2‐12* in the ‘deeppink’ module visualized using Cytoscape. *CarFAD2‐12* is marked in red; *FAB2* (*CtAH06G0083800*), *FAB1* (*CtAH05G0279500*) and *ROD1* (*CtAH09G0028100*) are shown in blue, and the positive regulators in ABA signalling pathway, *CPK4* (*CtAH06G0233500*) and *SNRK2.6* (*CtAH03G0292100*), are marked in yellow. Triangle nodes represent transcriptional regulators, and the node size represents the intra‐module connectivity of the genes.

To explore the differential regulation of the key gene families involved in LA and OA biosynthesis, we constructed a biosynthetic diagram of fatty acids based on the identified fatty acid composition and the KEGG database. Two pathways led to OA biosynthesis: one occurred via FAB2, which catalysed the conversion of stearic acid (18 : 0) to OA (18 : 1; Hwangbo *et al.,* 2013), while the other involved the activities of stearoyl‐CoA desaturase (SCD) and acyl‐coenzyme A thioesterase 1/2/4 (ACOT1_2_4) using stearoyl‐CoA as a substrate, which is also the sole pathway for LA biosynthesis (Figure 3a, Table S10).

Next, we performed a comparative transcriptome analysis of ‘HL’ seed formation at three stages: ovaries at 0 DAF (DAF0) and seeds at 10 DAF (DAF10) and 20 DAF (DAF20; Figure S19); seed germination at five stages: the cotyledons at 1, 3, 5, 7 and 10 days after germination (DAG1, DAG3, DAG5, DAG7 and DAG10, respectively; Figure S20); and flower development at five stages: small bud stage (SBS), middle bud stage (MBS), initial flowering stage (IFS), peak flowering stage (PFS) and decayed flowering stage (DFS; Figure S21). The LA content increased rapidly in ‘HL’ between DAF0 and DAF10 compared with the change between DAF10 and DAF20, while the OA content was highest at DAF0 and decreased at DAF20. LA but not OA could be detected in DAF0, suggesting that LA also accumulated in the ovaries before seed development began (Figure 3b).

Fatty acid desaturases (FADs) are of great importance in regulating plant fatty acid compositions. FAB2, FAD2 and FAD3 are desaturases involved in the biosynthesis of LA and OA; FAD2 is vital for converting OA to LA (Okuley *et al.,* 1994), while FAD3 controls the content of linolenic acid (18 : 3) through the desaturation of LA (18 : 2; Vrinten *et al.,* 2005). Of the 20 identified *FAD2*s, multiple tandem‐duplicated *FAD2*s (*CarFAD2‐1˜CarFAD2‐3*, *CarFAD2‐8˜CarFAD2‐10* and *CarFAD2‐15˜CarFAD2‐17*) were up‐regulated in ovaries, possibly contributing to ovary LA biosynthesis. *CarFAD2‐12* showed uniquely high expression in the ‘HL’ seeds at DAF10 followed by DAF20 compared to cotyledons and flowers (Figures 3c and S22). In the field‐planted ‘HL’ cultivar, *CarFAD2‐12* was also highly expressed at 10 DAF and 20 DAF, whereas it showed low expression levels at 10 DAF and 20 DAF in the ‘LL’ cultivar (Figure 3c; Table S11), which is consistent with the rapid accumulation of LA in ‘HL’ but not in ‘LL’ during seed development (Figure 3b). Sequencing of the 5ʹ UTR and coding sequence of *CarFAD2‐12* in ‘HL’ and ‘LL’ cultivars showed that there was single‐base deletion in the coding sequence of ‘LL’, resulting in premature termination of translation; the 5ʹ UTR showed complete identity between the two cultivars (Figure S23). Taken together, these observations suggest that *CarFAD2‐12* is key to converting OA to LA in safflower seeds.

A phylogenetic tree of the FAD2 family revealed that *CarFAD2‐12* was closely related to *CynFAD2‐1*, *LacFAD2‐2* and *HelFAD2‐27* in artichoke, lettuce and sunflower, respectively, and that this clade was separate from the *FAD2*s of non‐Asteraceae species (Figure S24; Table S12), all of which belong to the clade of *AtFAD2‐1*, defined by the gene responsible for the conversion of OA to LA in *Arabidopsis thaliana* (Lemieux *et al.,* 1990). *HelFAD2‐27* (*HanXRQChr14g0452931*) is highly expressed in developing high‐LA embryos, but its expression is extremely reduced in developing high‐OA embryos (Martinez‐Rivas *et al.,* 2001). These results indicated that one clade of Asteraceae FAD2s, including *CarFAD2‐12* and *HelFAD2‐27*, may have evolved as seed‐specific *FAD2s* and could contribute to the accumulation of high LA in this family.

To explore the relationship of *CarFAD2‐12* with other genes, we built a weighted gene coexpression network. In the coexpression network of seed development, *CarFAD2‐12* was contained in the ‘deeppink’ module, which comprised 114 genes that were co‐up‐regulated with the OA biosynthesis gene *FAB2* (*CtAH06G0083800*; Hwangbo *et al.,* 2013) at 10 DAF (Figure 3c,d). The genes in the adjacent ‘lightsteelblue1’ module were also co‐up‐regulated at 10 DAF. The ‘lightsteelblue1’ module contained genes associated with the ‘fatty acid biosynthetic process (GO:0006633)’, which included another *FAB2* homolog (*CtAH09G0227700*) and *FATA* (*CtAH10G0076400*), key genes for OA biosynthesis (Chen *et al.,* 2012a; Figures S25 and S26; Table S13). In addition, the homolog annotations for the ‘deeppink’ genes revealed that the fatty acid biosynthesis genes *FAB1* (*CtAH05G0279500*; Carlsson *et al.,* 2002) and *REDUCED OLEATE DESATURATION 1* (*CarROD1*, *CtAH09G0028100*; Hu *et al.,* 2012) were highly associated with *CarFAD2‐12* (Figure S27). As the key component in the ABA signalling pathway, SNRK2.6 was reported to be involved in unsaturated fatty acid biosynthesis. Genes encoding PKs in the ABA signalling pathway were also highly associated with *CarFAD2‐12*, such as *CALCIUM‐DEPENDENT PROTEIN KINASE 4* (*CarCPK4*, *CtAH06G0233500*) and *SUCROSE NONFERMENTING 1‐RELATED PROTEIN KINASE 2.6* (*CarSNRK2.6*, *CtAH03G0292100*; Figure 3e; Table S13), the homologs of which are positive regulators of the ABA signalling pathway in *Arabidopsis* (Zhu *et al.,* 2007).

**Figure 4:**
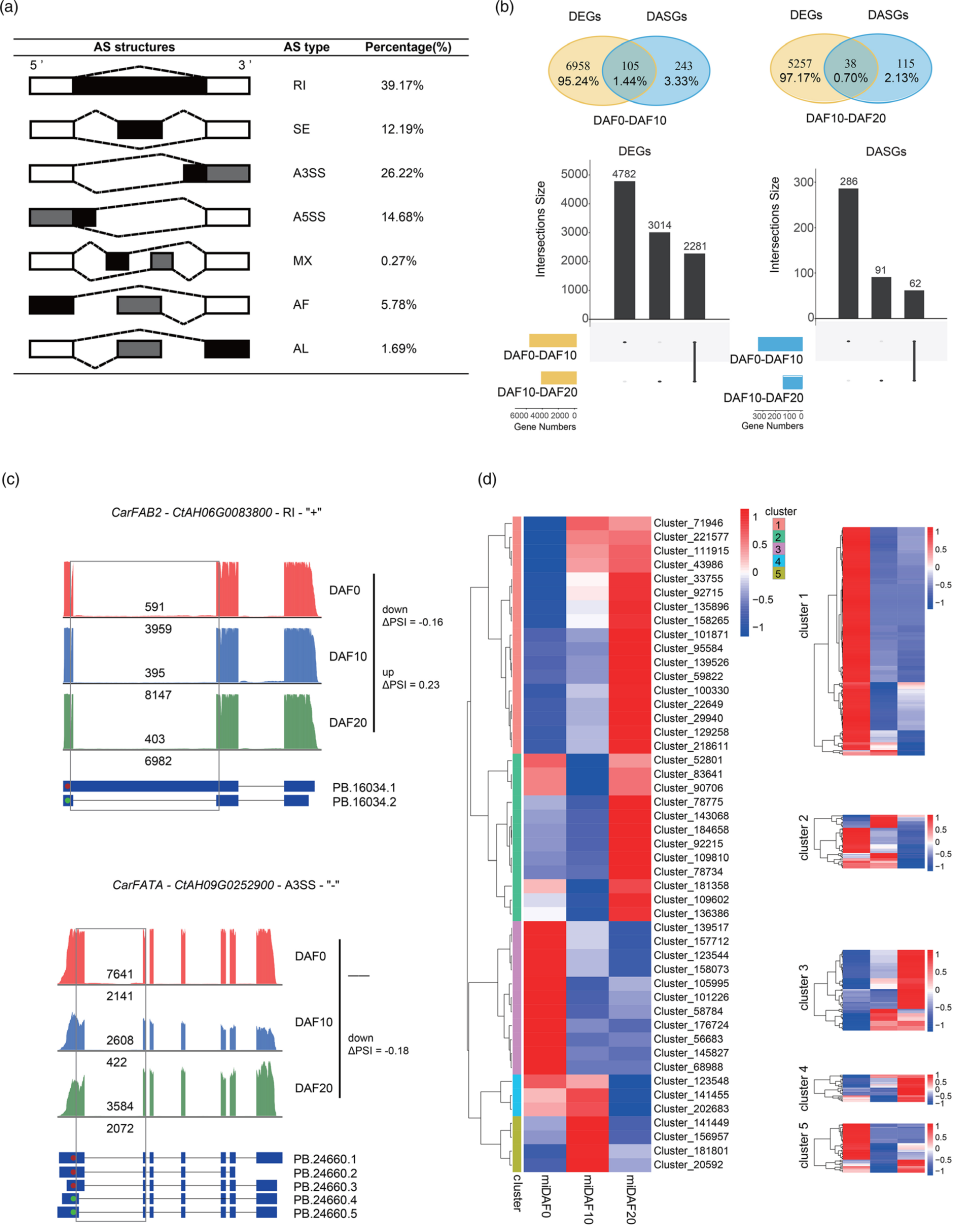
Gene regulation of the biosynthesis of linoleic acid (LA) and oleic acid (OA) in the ovaries and seeds of safflower. (a) Overview of the seven different types of alternative splicing (AS) and their frequency in the safflower seed formation. (b) Comparison of the differentially expressed genes (DEGs) and differentially alternatively spliced genes (DASGs) among the different developmental stages of the ovaries and seeds. Each vertical line at the bottom represents continuous DASGs among different stages. (c) AS variants for genes involved in OA biosynthesis. For each gene, AS‐covering and total long‐read counts are shown in AS variants in each stage of seed formation, and differential expression of AS variants is indicated by ΔPSI on the left. The ‘up’, ‘down’ and ‘‐’ on the left represent up‐regulated, down‐regulated, and no differential expression between adjacent groups, respectively. (d) Expression pattern of the miRNAs (left) and their targeted genes (right). The top right bar represents the normalized Z‐score values of miRNA FPKMs.

Analysis of the different splice isoforms in our data identified 10 664 AS events of seven types in seed formation: retained intron (RI), skipping exon (SE), alternative 5ʹ/3ʹ splice sites (A5SS/A3SS), mutually exclusive exons (MX) and alternative first or last exons (AF/AL). A high frequency of RI (39.17%) was identified, followed by A3SS, A5SS, SE, AF, AL and MX (Figure 4a). To investigate the relationship between the differentially expressed genes (DEGs) and the differentially AS genes (DASGs), we performed an adjacent comparison. We identified 403 AS events for 348 DASGs when comparing DAF10 seeds to DAF0 ovaries and 182 AS events in 153 DASGs when comparing DAF20 seeds with DAF10 seeds (Figure 4b). The number of DEGs was nearly 20 times the number of DASGs between the DAF10 and DAF0 samples and about 34 times the number of DASGs between DAF20 and DAF10. RI‐type AS of *CarFAB2* was down‐regulated at DAF10 versus (vs.) DAF0 and up‐regulated at DAF20 vs. DAF10, and A3SS‐type AS of *CarFATA* was down‐regulated at DAF20 vs. DAF10 (Figure 4c), indicating they may play key roles in OA biosynthesis. Some of the DASGs were involved in the related GO term ‘biosynthesis of fatty acids and unsaturated fatty acids’ during seed development, indicating that diverse AS events may be involved in the regulation of lipid accumulation (Figure S28). DEGs and DASGs had different enriched GO terms; lipid‐related GO terms were observed in DEGs, whereas the most enriched GO terms for DASGs were ‘vesicle‐mediated transport’ (up‐regulated in DAF10 relative to DAF0), ‘response to hormone’ (up‐regulated in DAF20 relative to DAF10), ‘DNA‐templated transcription, elongation’ (down‐regulated in DAF10 relative to DAF0) and ‘lipid biosynthetic process’ (down‐regulated in DAF20 relative to DAF10; Tables S14 and S15, Figures S29–S32). This also indicated that DEGs and DASGs might have different regulatory roles in the biosynthesis of fatty acids during seed development.

Diverse miRNAs have been reported to play important roles in fatty acid biosynthesis during seed development (Wang *et al.,* 2016). Here, we identified 52 miRNAs of 20–24, with 21 nt the most common during seed development; among these, 47 miRNAs corresponded to 295 potential target genes (Figure S33). The expression patterns of the miRNAs formed five clusters; cluster 1 contained the most miRNA genes, which were down‐regulated in the ovaries at 0 DAF and up‐regulated in the seeds at 10 DAF and 20 DAF. The target genes of cluster 1 miRNAs showed enrichment in ‘signalling’, while the targets of clusters 2 and 4 were both enriched in ‘developmental process’ (Figures 4d and S34, Table S16). Expression of Cluster_135896 showed negative correlation to its putative target gene, *CarFAD2‐4*, up‐regulated in ovary LA biosynthesis (Figures 3a and S35). In addition, four genes involved in the GO term ‘lipid biosynthetic process’ were regulated by miRNAs in cluster 1: *CtAH09G0055900* by Cluster_218611, *CtAH05G0100200* by Cluster_33755, *CtAH12G0118500* by Cluster_43986 and *CtAH10G0014200* by Cluster_95584. These results indicated that miRNAs may help regulate the fatty acid composition in safflower.

### Gene regulation of flavonoid biosynthesis during flower development

**Figure 5:**
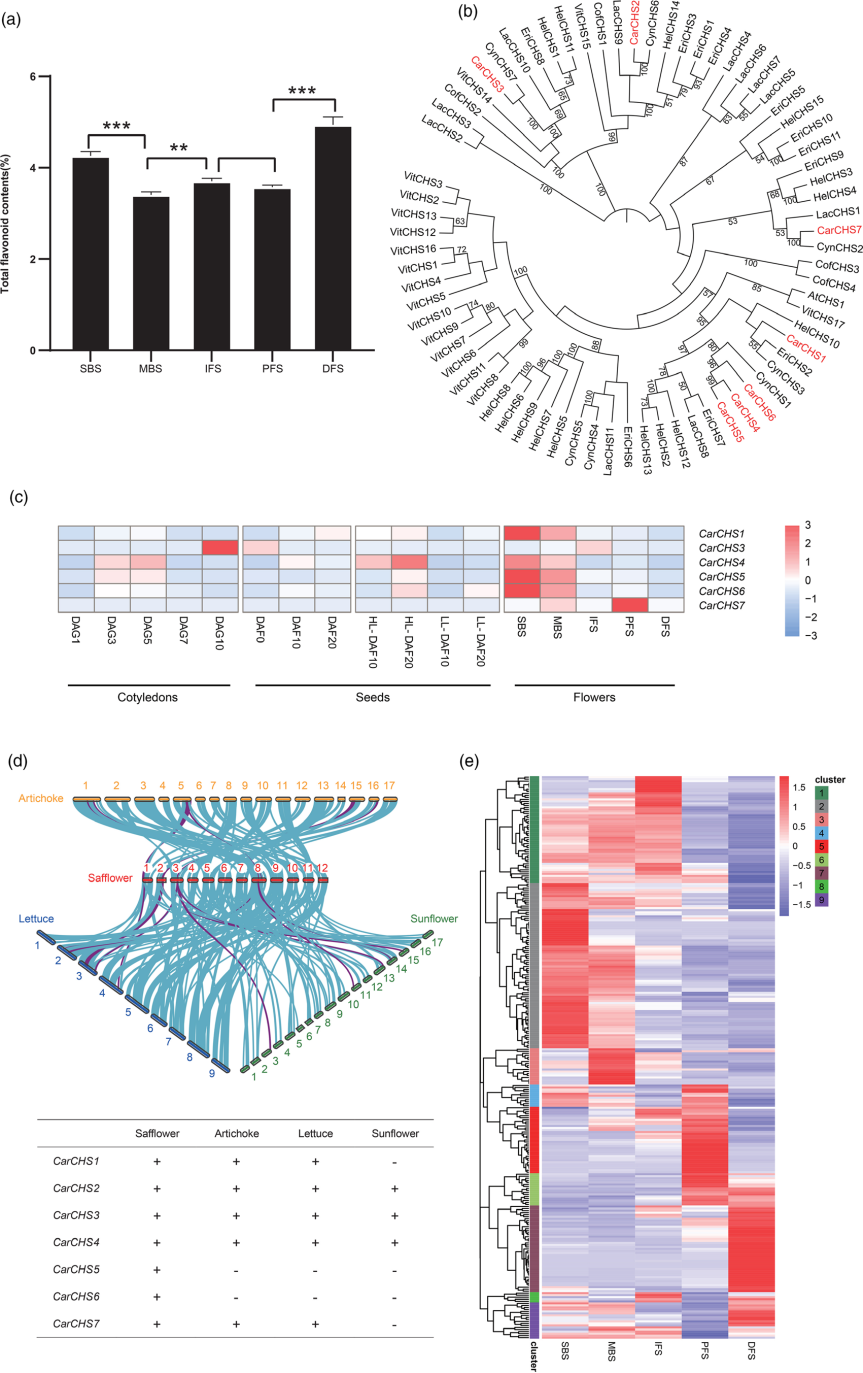
Gene families and clusters involved in the regulation of flavonoid biosynthesis. (a) Flavonoid content of flower filaments in the five stages of development. SBS, small bud stage; MBS, middle bud stage; IFS, initial flowering stage; PFS, peak flowering stage; DFS, decayed flowering stage. Values are means ± SD from three independent experiments. Student’s *t*‐test: *P < 0.05; **P < 0.01; ***P < 0.001. (b) Phylogenetic tree of the *CHS* gene families of eight species constructed by neighbour‐joining method. Only bootstrap values >50 are shown. Safflower *CarCHS*s are marked in red. (c) Expression pattern of the safflower *CarCHSs* except for *CarCHS2* not expressed in flowers. The top right bar represents the normalized Z‐score values of FPKMs. (d) Collinear block of flavonoid‐related gene clusters (top) and conserved collinearity of *CarCHSs* (bottom) among safflower, sunflower, artichoke and lettuce. (e) Expression patterns of gene clusters involved in flavonoid biosynthesis.

Among the five stages of flower development (SBS, MBS, IFS, PFS and DFS), flavonoids mainly accumulated during DFS (Figure 5a), and HSYA content was consistently higher than that of rutin, luteolin and quercetin across the five stages (Figure S36). In the conserved flavonoid biosynthesis pathway in plants, CHS is the first committed enzyme (Ferrer *et al.,* 2008). Based on our modified schematic pathway for flavonoid biosynthesis (Figure S37) integrated with the KEGG database and related literature (Chen *et al.,* 2020; Forkmann and Martens, 2001; Tu *et al.,* 2019), we identified flavonoid biosynthesis gene families ranging from the least common (cinnamate‐4‐hydroxylase, *C4H*, 2 genes) to the most populous (*UGTs*, 154 genes; Table S17). In safflower, overexpression of one *CHS* gene (*CtCHS1*) increases accumulation of HSYA in the quinochalcone biosynthetic pathway and up‐regulation of *CtCHS4* (Guo *et al.,* 2017). Seven *CHS* genes were identified in the safflower genome, and phylogenetic analysis showed that five *CarCHS*s (*CarCHS1* and *CarCHS4˜CarCHS7*) were closely related to *AtCHS* and were highly expressed specifically in the flowers, while the more distantly related members *CarCHS2* and *CarCHS3* were not expressed, or expressed in other non‐flower tissues, respectively (Figure 5b,c), indicating their regulatory divergence in safflower. Comparative analysis showed that *CarCHS4*, *CarCHS5* and *CarCHS6* were closely related to *CtCHS1* (with 100%, 97.9%, and 83.3% identity, respectively), while *CarCHS1* was closely related to *CtCHS4* with 90.8% identity (Figure S38).

**Figure 6:**
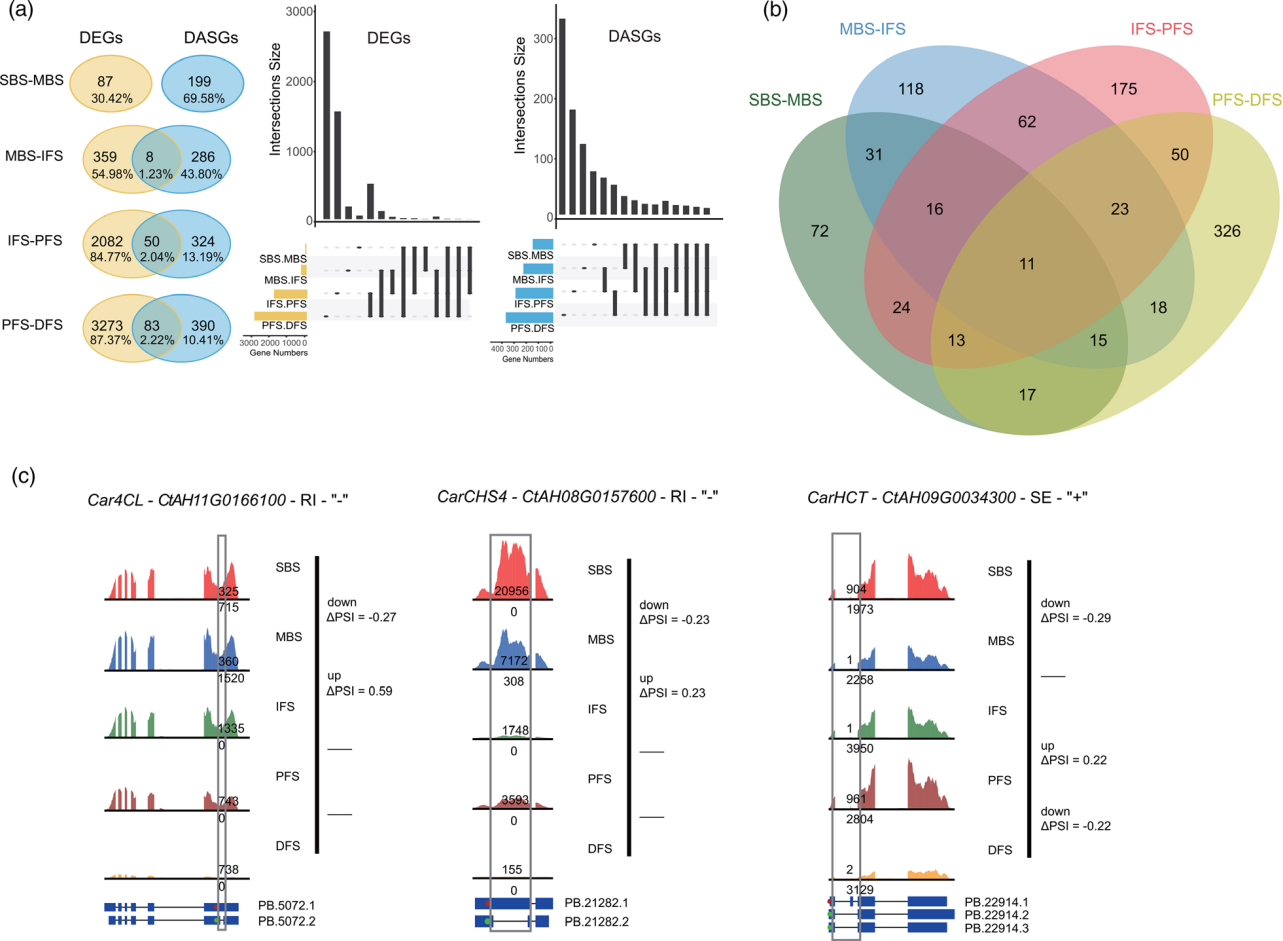
Differentially expressed genes (DEGs) and differentially alternatively spliced genes (DASGs) involved in flavonoid biosynthesis. (a) Comparison of DEGs and DASGs among the different stages of flower development. Each vertical line at the bottom represents continuous DASGs among different stages. (b) Venn of DASGs in each adjacent comparison. (c) Identified alternative splicing (AS) variants for genes involved in flavonoid biosynthesis. For each gene, AS‐covering and total long‐read counts are shown in AS variants in each stage of seed formation, and differential expression of AS variants is indicated by ΔPSI on the left. The ‘up’, ‘down’ and ‘‐’ on the left represent up‐regulated, down‐regulated and no differential expression between adjacent groups, respectively.

The coexpression of metabolism‐related genes present in species‐specific physical clusters within the genome (defined as gene clusters) can illustrate the divergent evolution of specialized metabolism in different plants (Chae *et al.,* 2014). Collinearity analysis revealed that most flavonoid biosynthetic genes, including *CarCHS1*, *CarCHS2*, *CarCHS3*, *CarCHS4* and *CarCHS7*, showed conserved collinearity among safflower, artichoke, lettuce and sunflower, while 18 gene clusters (such as the cluster of *CarCHS5* and *CarCHS6*) were unique to safflower (Figure 5d, Table S17). Combining the collinearity analysis with the phylogenetic analysis (Figure 5b), we identified a tandem duplication of *CarCHS5* and *CarCHS6* duplicated from the HSYA biosynthetic gene *CarCHS4*; this duplication only occurred in safflower after its divergence from the other Asteraceae species (Figures 5d and S39). Through CHS, the intermediate naringenin chalcone may be further catalysed by UGTs to generate HSYA based on their similar chemical structures (Tu *et al.,* 2019). Co‐up‐regulated with *CarCHS4* and multiple UGTs in cluster 2, the unique duplication of *CarCHS5* and *CarCHS6* may further contribute to HSYA biosynthesis in safflower (Figure 5e; Table S17). Further AS analysis showed little overlap between DEGs and DASGs, as well as between the top 20 enriched GO or KEGG terms for DEGs and DASGs (Figures 6a,b and S40–S46). This indicated the diverse regulation of DEGs and DASGs during flower development, which was also reported in other plants, such as rice (*Oryza sativa*; Dong *et al.,* 2018). Besides DEGs, AS events for genes in the flavonoid pathway, such as *CarCHS4*, *Car4CL* (*CtAH11G0166100*) and *CarHCT* (*CtAH09G0034300*), may be another mechanism regulating total flavonoid and HSYA biosynthesis (Figure 6c). Compared to intron‐splicing AS variant (PB.21282.1), which lacks the C‐terminal domain of chalcone and stilbene synthases, the RI‐type AS variant (PB.21282.2) of *CarCHS4* was down‐regulated at MBS vs. SBS but up‐regulated at IFS vs. MBS (Figures 6c and S47). High expression of the RI‐type AS variant (PB.21282.2) occurred at the SBS stage (Figure 6c) where HSYA accumulated, indicating that there may be another mechanism regulating *CarCHS4* and HSYA biosynthesis at the AS level. All these results suggested that the diverged regulation of flavonoid biosynthetic genes could involve synergistic changes at the genomic and transcriptomic levels.

## Discussion

In this work, we report a chromosome‐scale genome sequence of safflower with high‐LA content; our analysis provides important insights into the genomic landscape of safflower, opening a route to functional and molecular breeding of this economically and medicinally important crop. Besides expansion of activation of the ABA signalling pathway at the genomic level, expansion of the safflower *FAR1* family may be related to its moderate tolerance of abiotic stresses and extensive branching (Hussain *et al.,* 2015). For example, the expansion of the *FAR1* family that we observed in the safflower genome may underlie the high adaptability of safflower to extreme environments. FAR1 is a Mutator‐like transposase‐derived TF that is essential for phytochrome A‐mediated far‐red light signalling in *Arabidopsis* (Lin *et al.,* 2007). *FAR1* is widely distributed in the angiosperms, but not in other organisms, and is involved in diverse physiological and developmental processes, such as chlorophyll biosynthesis, circadian clock entrainment, ABA signalling and branching (Wang and Wang, 2015; Xie *et al.,* 2020). The evolution of *FAR1* within the safflower genome may have enhanced their fitness and adaptation to complex living environments by integrating various endogenous and exogenous signals for the coordinated regulation of growth and development (Wang and Wang, 2015).

Based on our findings, we conclude that duplication of *FAD2* genes along with their regulatory divergence contributed to LA biosynthesis in ovaries and seeds. In comparison with animals, WGD or gene duplication events are much more common in plants and have contributed to many of the successful innovations of the angiosperms (Jiao *et al.,* 2011). These specialized innovations have been attributed to WGDs, tandem duplication through unequal crossing over, transposon‐mediated gene duplication, segmental duplication and retro‐duplication events (Panchy *et al.,* 2016), as well as gene regulation at the transcriptional level (Chen *et al.,* 2012b). For example, the genomic architecture of flowering time has been shaped by the most recent WGD in sunflower (Badouin *et al.,* 2017). In safflower, tandem duplications of *FAD2s* were distributed on chromosomes 2 (*CarFAD2‐1*˜*CarFAD2‐3*), 7 (*CarFAD2‐7*˜*CarFAD2‐10*) and 12 (*CarFAD2‐16*˜*CarFAD2‐20*), and WGD or segmental duplication occurred between *CarFAD2‐12* and *CarFAD2‐14* and among *CarFAD2‐5*, *CarFAD2−13* and *CarFAD2‐15* (Figure S22). *FAD2* members created by tandem duplication were coexpressed and contributed to LA biosynthesis in ovaries, whereas *CarFAD2‐12*, which was derived from WGD, was highly expressed in seeds and vital for LA biosynthesis there. These patterns suggest that the different fates of duplicated *FAD2* genes result in the complexity of LA biosynthesis in different organs of safflower.

Our data indicate that *CarFAD2‐12* is likely regulated at multiple levels. Previous research showed that ABA plays a crucial role in fatty acid biosynthesis during seed maturation (Nguyen *et al.,* 2016). SnRK2.6, a positive regulator of ABA signalling in *Arabidopsis*, is involved in unsaturated fatty acid biosynthesis (Mustilli *et al.,* 2002; Zhu *et al.,* 2007). Besides being highly coexpressed with SnRK2.6, compared to other *CarFAD2* members, the promoter of *CarFAD2‐12* has a unique ABA‐responsive cis‐regulatory element (ABRE, GACACGTACGT) in addition to the common ABREs (ACGTG) predicted by PlantCARE (Lescot *et al.,* 2002). These results suggest that ABA signalling induces *CarFAD2‐12* expression. A long intron in the 5′ UTR plays a role in the enhancement of *FAD2* expression and further regulates LA content in seeds (Salimonti *et al.,* 2020). Compared to other safflower *CarFAD2*s, only *CarFAD2‐12* has a large intron in the 5′ UTR (Figure S48). Despite several *HelFAD2*s in sunflower having 5′ UTR introns, a gene tree constructed by protein sequences showed that only *HelFAD2‐27* with a large 5′ UTR intron, closely related to *CarFAD2‐12* and *AtFAD2‐1*, was up‐regulated in high‐LA embryos. Our comparative analysis between high‐LA and low‐LA safflowers showed that *CarFAD2‐12* had a single‐base deletion in the coding region in low‐LA safflower with the complete 5′ UTRs, causing its extreme down‐regulation. These findings suggest that *CarFAD2‐12* is regulated by both the UTR and coding region. In addition, *CarFAD2‐12* was coexpressed with several TFs and TRs, such as *MYB12*‐like (*CtAH11G0218800*), heat stress transcription factor *C‐1*‐like (*CtAH12G0115000*), ethylene‐responsive transcription factor *TINY*‐like (*CtAH05G0018300*), heat shock factor (HSF, *CtAH09G0091400*), auxin‐responsive protein IAA26 (*CtAH09G0016800*) and IAA13 (*CtAH06G0080400*). These TFs and TRs are strong candidates for further investigation in the regulation of LA biosynthesis.

The unique tandem duplication of *CarCHS*s and their coexpressed genes in the flower likely contributes to the biosynthesis of flavonoids such as HSYA. Since the first discovery of safflower HSYA (Meselhy *et al.,* 1993), ongoing efforts have been made to identify genes involved in HSYA biosynthesis despite the lack of the safflower genome sequence. However, the biosynthetic pathway of HSYA remains undetermined. The CHS family has been reported to undergo duplication and adaptive evolution in Asteraceae (Yang *et al.,* 2002). *CarCHS4* is thought to play an important role in HSYA biosynthesis rather than flavonol biosynthesis in safflower (Guo *et al.,* 2017). Tandemly duplicated *CarCHS5* and *CarCHS6* showed not only close relationships to *CarCHS4* in the phylogenetic tree (Figures 5b,d and S39) but also co‐up‐regulation with *CarCHS4* during flower development (Figure 5e), indicating this duplication would contribute to HSYA accumulation. The expression of flavonoid biosynthetic genes is regulated by MYB and bHLH TFs (Dubos *et al.,* 2010; Goossens *et al.,* 2017). Multiple enzymes (such as UGTs) and TFs (such as bHLHs and MYBs) coexpressed with *CarCHS*s would contribute to the complexity of flavonoid biosynthesis in safflower (Figure S49). For example, the antisense RNA (*CT‐wpr*) of the coexpressed *CtAH10G0189700* (encoding a member of the aspartyl protease family, ASP) was significantly associated with the presence of HSYA and was up‐regulated in flowers lacking HSYA (Li *et al.,* 2010). By contrast, *CtAH10G0189700* was up‐regulated in flowers containing HSYA compared with flowers lacking HSYA. Natural antisense transcripts are endogenous RNA molecules containing sequences that are complementary to other transcripts and can negatively regulate the corresponding sense transcript (Lapidot and Pilpel, 2006). Therefore, the sense transcript (*CtAH10G0189700*) was possibly regulated by its antisense transcript (*CT‐wpr*), which likely functions as a noncoding RNA to suppress the HSYA biosynthetic pathway (Li *et al.,* 2010).

HSYA biosynthesis in safflower flowers is more complicated than expected. Small heat shock proteins (sHsps) have an unusual diversity of functions in response to environmental stress and development. Linkage analysis of cDNA‐AFLP identified one HSYA‐associated gene (*CTL‐hsyapr*), encoding a sHsp, which was up‐regulated in the HSYA‐absent lines and had strong linkage with HSYA in a separate F_2_ population (Tang *et al.,* 2010). Expression of *CTL‐hsyapr* disturbed the HSYA biosynthetic pathway in flowers lacking HSYA. By BLAST searching against our safflower genome, we determined that the closest homolog (*CtAH12G0163400*) of *CTL‐hsyapr* is also up‐regulated in white flowers lacking HSYA, compared to flowers with HSYA. Moreover, HSYA biosynthesis can be promoted by an exogenous inducing factor, methyl jasmonate, via up‐regulating the expression of upstream genes in the flavonoid biosynthesis pathway (such as *CHSs*; Chen *et al.,* 2020). Therefore, genes involved in normal growth, development and stress may contribute to the regulation of safflower HSYA biosynthesis.

## Conclusion

This sequence for the safflower genome provides insights into the molecular regulation of fatty acids in seeds, as well as of flavonoids in flowers. The comprehensive safflower transcriptome data we provide here will be valuable for fundamental biological research and applied breeding programs. Our data highlight the importance of elucidating the genomic structure, evolution and expression of *FAD2* and *CHS* families, and suggest that future functional research into their coexpressed candidate genes could be instrumental in improving the fatty acid and flavonoid content in safflower. This study also lays the foundation for research into Asteraceae taxonomy, further enhancing our understanding of the evolution, phytochemistry and ecology of safflower.

## Methods

### Samples collected for genome assembly and transcriptomic analysis

For genomic sequencing, the ‘HL’ safflower (*Carthamus tinctorius* L.) cultivar ‘Anhui‐1’ was cultivated in the field in Wuhan, China, in 2017. For RNA‐seq, the seeds of ‘HL’ safflowers were soaked in sterilized water for 24 h in a Petri dish, then germinated for 1, 3, 5, 7 and 10 days under a 16‐h light/8‐h dark photoperiod (10 000 lux) in a 24–26 °C culture chamber. Three replicates of the safflower cotyledons were sampled and stored in a refrigerator at −80 °C. ‘HL’ safflowers were grown in a greenhouse, and the seeds were sampled at 0, 10 and 20 days after flowering. Fifteen replicates were performed. The flowers of the ‘HL’ safflowers were sampled at five developmental stages, small buds at bud stage I, medium buds at bud stage II, flowers at initial flowering stage, flowers at peak flowering stage and flowers at decayed flowering stage. Three replicates were taken at each stage. The roots, shoots, leaves, callus, flowers, ovaries and seeds mentioned above were mixed for the full‐length transcript sequencing.

### *Denovo* genome assembly

Genomic DNA was extracted using the modified CTAB method and sequenced using PacBio Sequel (Pacific Biosciences) combined with Illumina HiSeq 2500 sequencing for correction. The genome size and heterozygosity of safflower were determined using flow cytometry and *k*‐mer frequency analysis. The 17 *k*‐mer distribution displayed one major peak. The *k*‐mer depth of 33 was determined as the main peak depth of the *k*‐mer frequency distribution using 150‐bp Illumina paired‐end reads (35 Gb).

Briefly, we performed de novo assembly using Canu (version 1.3; Koren *et al.,* 2017) and Falcon (Chin *et al.,* 2016), and found Canu (N50 = 16.43 Mb with 368 contigs) is better than Falcon (1.40 Mb with 3195 contigs). Then, the draft genome from Canu assembly was furthered assembled into scaffold whit Hi‐C. Then, we used the GPM pipeline (Zhang *et al.,* 2016) to fill gaps of 12 superscaffolds (N50 = 14.17, 213 contigs; Superscaffold N50 = 88.21Mb) from Hi‐C with Falcon contigs after filtering then unanchored short contigs. Finally, the consensus sequence was corrected using the arrow method implemented in the SMRTLink and further polished with long reads using Pilon (version 1.22; Walker *et al.,* 2014) to get a final version (N50 = 21.23 Mb with 128 contigs).

### Chromosome assignment using both Hi‐C and a linkage map

The resulting high‐quality Hi‐C data following digestion with Hind III were used to assist the construction of chromosome‐level assemblies. The assembled scaffolds were ordered and oriented into chromosomes using ALLMAPS (Tang *et al.,* 2015) combined with our constructed genetic linkage map.

To anchor the scaffolds, a high‐density genetic linkage map was developed using the 144 lines of the F_2_ population derived from a cross between AH04 and YH04, which were genotyped using 1160 pairs of simple sequence repeat (SSR) primers and 1255 pairs of InDel primers. The genetic map spanned 1136.46 cM and contained 96 SSR molecular markers and 155 InDel molecular markers. Using QTL IciMapping (Meng *et al.,* 2015), around 988.92 Mb could be anchored to 12 chromosomes using the genetic linkage map, which covered 93.5% of the genomic assembly.

### Identification of repetitive genomic elements

The Repbase database and a *de novo* repeat library were used to annotate the repeated DNA sequences in the safflower genome. First, Repbase was downloaded from http://www.girinst.org/repbase/ and a *de novo* repeat library was generated from the assembled safflower genome using RepeatModeler (version open‐1.0.11; http://repeatmasker.org/RepeatModeler/). Second, the repetitive elements in Repbase and the safflower *de novo* repeat library were annotated using RepeatMasker (version open‐4.0.7).

### Annotation of protein‐coding genes

MAKER (Campbell *et al.,* 2014) was used to annotate the protein‐coding genes of safflower based on three different approaches: *de novo* prediction, homology‐based prediction and transcript‐based prediction (Campbell *et al.,* 2014). For the *de novo* prediction using Augustus (version 3.3.1; Keller *et al.,* 2011), tomato (*Solanum lycopersicum*) was used as the species model. To perform the *de novo* assembly of transcripts from our RNA‐seq data, the reads were trimmed using Trimmomatic (version 0.36; Bolger *et al.,* 2014) and assembled in Trinity (version 2.8.4; Grabherr *et al.,* 2011). The full‐length high‐quality transcripts from PacBio Sequel I were also identified using Isoseq3 (https://github.com/ben‐lerch/IsoSeq‐3.0) and corrected with proovread (version 2.14.1; Hackl *et al.,* 2014).

Using the assembled genome and RNA‐seq data, STAR (Dobin *et al.,* 2013), GeneMark_ET (version 4.38; Lomsadze *et al.,* 2005), HISAT2 (version 2.1.0; Pertea *et al.,* 2016) and StringTie (version 1.3.4d; Kovaka *et al.,* 2019) were used to predict the genes. For the homology‐based approach, the non‐redundant proteins from *Arabidopsis thaliana*, *Chrysanthemum nankingense*, *Helianthus* *annuus* and *Lactuca sativa* and the proteins of uniport sprot from the Swiss‐Prot database were jointly used to annotate the identified genes in MAKER. SNAP (version 2006‐07‐28; Korf, 2004) was also integrated into MAKER to train the gene models. The resulting gene annotation was evaluated by identifying the complete BUSCO hits (Simao *et al.,* 2015) and the mapping rates of the DNA and RNA reads from the Illumina sequencing.

Following the gene annotation, the singleton and duplicated genes were identified using MCScanX (http://chibba.pgml.uga.edu/mcscan2/). Based on the AEK, the evolutionary route of the genes was constructed for safflower and the other Asterids in MCScanX. The potential protein sequences encoded by each gene were subjected to a BLAST analysis against the NCBI non‐redundant protein database to identify homologous proteins in other species using diamond (version 0.9.24.125; Buchfink *et al.,* 2015). The functional domains and possible GO terms in the protein sequences were identified in InterProScan (Jones *et al.,* 2014). The genes were annotated with KEGG terms using eggNOG‐mapper (version 2; Huerta‐Cepas *et al.,* 2017). The standalone iTAK (Zheng *et al.,* 2016) program was used to predict the transcriptional factors, transcriptional regulators and protein kinases in the safflower data set.

### Gene family and phylogenomic analysis

Gene families for the ten analysed species (Table S7) were clustered using OrthoFinder (version 2.2.7) with default parameters (Emms and Kelly, 2015). A species tree for the ten species was inferred from a joint matrix of coding sequences from the orthogroups with a single‐copy ortholog for each species using RaxML (version 8.2.12; Stamatakis, 2014). Based on a calibration of divergence times using the Rosids and Asterids (>1.1 and <1.3 Mya) and Asteraceae and non‐Asteraceae (>0.8 Mya; Barreda *et al.,* 2015) divergences, the divergence times for the inferred species tree were calculated using MCMCtree implemented in PAML (version 4.8; Rannala and Yang, 2007). The divergence times were then recalculated to check the convergence of the two independent predictions using Pearson’s correlation coefficient. The phylogenetic tree was visualized using the R package MCMCtreeR (version 1.1). Gene families inferred from OrthoFinder were used to calculate the expansion or contraction of the gene families in each lineage using CAFÉ (version 4.2; De Bie *et al.,* 2006). The GO and KEGG enrichments of the genes in unique, expanded and contracted families were analysed using the SEA method implemented in eggNOG‐mapper (version 2; Huerta‐Cepas *et al.,* 2017).

### Investigation of WGD events

To study the evolution of the safflower genome, the genome‐wide duplications present in the assembled safflower genome were identified. The safflower genome was compared with those of six other plant species (grape, robusta coffee, sweet wormwood, chrysanthemum, lettuce and sunflower). The all‐vs‐all paralog analysis within each species was performed using the reciprocal best hits from the primary protein sequences in these species using self‐BLASTp (BLAST 2.7.1+). To detect possible small‐scale background duplications, a synteny analysis was performed on the safflower genes using MCScanX using default parameters from the top five self‐BLASTp hits, and on the orthologs between the seven species using the reciprocal best hits from the primary protein sequences. The number of synonymous substitutions per synonymous site (*Ks*) was calculated for each gene pair using ParaAT and KaKs_Calculator (version 2.0), based on the YN model (Wang *et al.,* 2010).

### Measurements of flavonoid contents in the flowers at five stages

Ten mg of rutin was dissolved in a small amount of methanol to prepare a standard solution with a concentration of 0.1 mg/mL. For the preparation of the standard curve, 0, 0.4, 0.8, 1.2, 1.6, 2.0 and 2.4 mL of the rutin standard solution were transferred into separate 10‐mL volumetric flasks and combined with 0.4 mL 5% sodium nitrite, 0.4 mL 10% aluminum nitrate and 4 mL of 1 M sodium hydroxide. The absorbance values of the combined solutions with different concentrations were measured at a 510‐nm wavelength using a UV spectrophotometer. The regression equation was obtained: *A* = 15.13*C* + 0.0056, *R* ^2^ = 0.9996, where A is the absorbance and C is the rutin concentration.

To measure the flavonoid content of flowers, 0.5 g of powdered flower tissue was dissolved in 20 mL methanol in a round bottom flask, then heated and refluxed at 80 °C for 40 min, cooled and filtered at room temperature. The total flavone content (%) was calculated as (*C* × *n* × *V*_0_ × 10^−3^)/m × 100, where C is the concentration of total flavonoids (mg/mL), n is the dilution ratio of the total flavonoids, V_0_ is the total volume of the constant volume (mL) and m is the flower quality (g). The statistical analysis of the flavonoid contents at the different stages of flower development was performed using a one‐way ANOVA. Similarly, HSYA, rutin, luteolin and quercetin in flowers were quantified according to their corresponding standards.

### Measurement of the fatty acid contents of the ovaries and seeds

The fatty acid composition was determined for the seeds of two cultivars, ‘HL’ and ‘LL’, planted in the autumn at 10 and 20 days after flowering. Three replicates for the seeds at each stage were performed. In addition, the total fatty acids in ‘HL’ developing seeds planted in the spring were extracted using the reflux method, after which the OA and LA contents were quantified using a liquid chromatograph, Agilent Technologies 1260 LC. The statistical analysis among the samples at different stages was performed using a one‐way ANOVA.

### Transcriptomic analysis of short reads from Illumina sequencing

The quality of the primitive RNA‐seq reads was evaluated using FastQC (version 0.11.7; http://www.bioinformatics.babraham.ac.uk/projects/fastqc/), and poor‐quality reads were trimmed using Trimmomatic (version 0.38; Bolger *et al.,* 2014). The cleaned high‐quality RNA reads were used for the *de novo* assembly of transcripts using Trinity (version 2.1.1; Grabherr *et al.,* 2011), providing EST evidence for the genome annotation. To estimate the expressed abundance of the annotated safflower genes, the clean reads were aligned against reference genome using HISAT2 (version 2.0.4; Pertea *et al.,* 2016). The genes were quantified with FPKMs using StringTie (Kovaka *et al.,* 2019), and differentially expressed genes were identified using DESeq2 (Love *et al.,* 2014).

The gene coexpression network for 45 samples, including DAF0 ovaries, DAF10 and DAF20 seeds, was constructed with a weighted gene coexpression network analysis (Wu *et al.,* 2019). GO and KEGG enrichment analyses for the modules were performed using clusterProfile (Yu *et al.,* 2012). The miRNAs were identified using ShortStack (Shahid and Axtell, 2014), and their targets were predicted using psRNATarget.

### Full transcript analysis for long reads from Pacbio sequencing

Full transcript identification: Polished representative circular consensus sequences were generated from the PacBio subreads raw data in ccs (version 4.1.0, https://github.com/PacificBiosciences/ccs), and the high‐quality reads (>99% accuracy) were generated using polish. Full‐length non‐chimeric reads were obtained using ‘pbtranscript classify’ implemented in SMRTLink (version 6.0.0.47841) with the default parameters. The pbtranscript cluster tool was used to cluster the polished reads without the polish, after which the reads were corrected with Illumina data using LoRDEC (version 0.9; Salmela and Rivals, 2014). The mapping of reads to the safflower genome assembly was carried out using minimap2 (version 2.17; Li, 2018). Further, the cDNA_Cupcake package (version 8.7, https://github.com/Magdoll/cDNA_Cupcake) was used to collapse the redundant transcripts.

DEG and DASG analysis: The RNA‐seq data comprised sequences from flowers from five developmental stages, ovaries at 0 DAF and seeds from 10 and 20 DAF. The RNA‐seq raw reads were filtered to remove the adaptors and low‐quality bases using Trimmomatic version 0.38 (Bolger *et al.,* 2014). The filtered reads were aligned to the safflower genome using HISAT2 (version 2.0.4; Pertea *et al.,* 2016), utilizing the PacBio full‐length transcript annotations. The TPM (Transcripts Per Kilobase Million) values, FPKM values and read counts were calculated using StringTie (version 1.3.4d; Kovaka *et al.,* 2019). For the differential splicing analysis, SUPPA2 (version 2.3; Trincado *et al.,* 2018) was used to identify the AS events in the full‐length transcripts from the PacBio data, and to calculate the percent spliced in index (PSI) value to quantify the AS event inclusion levels by the TPM values of transcripts from multiple samples. Differentially AS events between two consecutive developmental stages were identified using SUPPA2 if the difference in the PSI of the AS event between the two stages exceeded a stringent threshold (*P*‐value < 0.05, |ΔPSI| > 0.1). For the differential expression analysis, DESeq2 (Love *et al.,* 2014) was used to identify the DEGs with false discovery rate (FDR) < 0.05 and |fold change| > 2.

### Identification of miRNAs and prediction of their targeted genes

The ovaries at 0 DAF and seeds from 10 and 20 DAF were sequenced on an Illumina HiSeq X‐ten, with a read length of 50 bp. Shortstack (version 3.8.4; Axtell, 2013) was used to identify the miRNAs from these tissues. To annotate the miRNAs, the identified miRNAs were mapped to the miRBase database using BLAST (version 2.9.0+). The results were filtered using the criteria of no more than two mismatches and one InDel between the query miRNA sequence and a known miRNA. psRNATarget (http://plantgrn.noble.org/psRNATarget/) was used to identify the target gene of each miRNA. The candidate target genes were screened using an Expectation ≤ 3.5, and the target gene was reversed if its expression was negatively correlated (Pearson’s correlation coefficient < −0.5) with the miRNA expression across multiple stages. The miRNAs were clustered using the hcluster method, and the function of each miRNA’s target gene was analysed by cluster. DESeq2 was used to identify the differentially expressed miRNAs, with FDR < 0.05 and |fold change| > 2.

### Genome mining for gene clusters

Based on the results of the KEGG and GO annotations of the safflower genome, metabolic gene clusters were selected based on two criteria: (1) all annotated genes in each cluster must be associated with at least a KO (KEGG Orthology) number and (2) all metabolic‐related genes in a cluster must be contiguously located on the same chromosome. Further, each cluster that contained at least two KO numbers involved in a flavonoid or fatty acid biosynthesis pathway was considered to be a flavonoid‐ or fatty acid‐associated gene cluster.

### Identification of gene families involved in flavonoid and unsaturated fatty acid biosynthesis

The flavonoid biosynthesis pathways (ko00941, ko00940, ko00944 and ko00942) in the KEGG database (https://www.genome.jp/kegg/) and reported in related literature (Chen *et al.,* 2018) were used to identify the enzymes involved in each step. The KEGG biosynthesis of unsaturated fatty acids (ko01040) category was used as a reference to map the enzymes of unsaturated fatty acids onto the pathway. The protein sequences of gene families involved in unsaturated fatty acids and flavonoid biosynthetic pathways were obtained from the KEGG database. All gene family members potentially involved in the unsaturated fatty acid and flavonoid biosynthetic pathways in safflower and other representative plants (*Artemisia annua*, *Coffea canephora*, *Vitis vinifera*, *Chrysanthemum nankingense*, *Cynara cardunculus*, *Erigeron breviscapus*, *Helianthus* *annuus* and *Lactuca sativa*) were identified using a Python script, and domains with an e‐value < 1E−5 were further retained using interproscan version 5.39‐77.0 (Jones *et al.,* 2014). MUSCLE version 3.8.1551 (Edgar, 2004) was used to perform a multi‐sequence alignment on the results, and the neighbour‐Joining method with 500 bootstrap repetitions was performed in MEGAX (Kumar *et al.,* 2018) for the inference of the gene trees among the gene families.

### Accession numbers

The genome assembly and annotations used in this study are available at our safflower genome database (http://safflower.scuec.edu.cn). All the raw sequencing data generated during this study have been deposited at NCBI as a BioProject under accession PRJNA642978. Transcriptome sequence reads have been deposited in the SRA database under BioProject number PRJNA646045.

## Conflict of interest

The authors declare no competing interests.

## Author contributions

J.Z. and R.Q. designed and supervised the research. Z.W. and J.Z. performed the genome assemblies and annotation. Z.W., H.L. and T.Y. performed the phylogenomic analysis. W.Z., N.X., Y.C., G.L. and J.L. measured the contents of fatty acid and flavonoid, and constructed the genetic linkage map. Z.W., Z.Y., E.Q. and S.L. analysed RNA‐seq data. D.K. and S.L. performed PacBio sequencing. R.W., X.Z. and H.X. provided constructive comments and suggestions on data analysis. C.X. performed the flow cytometry experiment. Z.W. and H.L. wrote the paper with input from all other authors. All authors approved the paper.

## Supporting information

**Figure S1** Evaluation of safflower (*Carthamus tinctorius*) genome size estimated using a *k*‐mer frequency analysis (a) and flow cytometry using soybean (*Glycine max*) as a control (b).
**Figure S2** The Hi‐C interacted heatmap for chromosome‐scale genome assembly.
**Figure S3** The high‐density genetic linkage map of safflower constructed from the F_2_ population of a cross between the parents AH04 and YH04.
**Figure S4** Synteny plot between our assembled safflower genome using Pacbio and Hi‐C, and the published draft genome of safflower generated using Illumina Hi‐Seq.
**Figure S5** Characteristics of the repetitive elements in the safflower genome.
**Figure S6** Identification and classification of long noncoding RNAs according to their position in the safflower genome.
**Figure S7** Gene Ontology categories associated with the annotated genes in the safflower genome.
**Figure S8** The number of transcriptional factors (TFs), transcriptional regulators (TRs), and protein kinases (PKs) in the safflower (*Carthamus tinctorius*) and nine other plant genomes.
**Figure S9** Proportions of transcriptional factors (TFs), transcriptional regulators (TRs), and protein kinases (PKs) in the safflower and nine other plant genomes.
**Figure S10** Types of gene duplication in the safflower (*Carthamus tinctorius*) genome and five other plant species.
**Figure S11** Syntenic depths in the artichoke versus safflower genome comparison.
**Figure S12** Enrichment of biological process GO terms (a) and KEGG pathways (b) associated with the gene families specific to safflower with a q‐value <0.05.
**Figure S13** Enrichment of biological process GO terms (a) and KEGG pathways (b) associated with the expanded gene families in safflower with a q‐value <0.05.
**Figure S14** Enrichment of biological process GO terms (a) and KEGG pathways (b) associated with the contracted gene families in safflower with q‐value <0.05.
**Figure S15** Safflower seed oil content and fatty acid composition of ‘HL’ (high linoleic acid) and ‘LL’ (low linoleic acid) cultivar plants.
**Figure S16** Sample distance of 12 RNA‐seq samples of ‘HL’ (high linoleic acid) and ‘LL’ (low linoleic acid) cultivar seeds at 10 days after flowering (DAF) and 20 DAF.
**Figure S17** Venn diagram of four sets of differentially upregulated and downregulated genes in DAF20 versus DAF10 of ‘HL’ and ‘LL’ cultivars.
**Figure S18** Enrichment of biological process GO terms of 328 uniquely upregulated genes in DAF20 versus DAF10 in the ‘HL’ cultivar compared with ‘LL’ cultivar.
**Figure S19** Sample distance of 45 RNA‐seq samples, including 15 ovaries from the ‘HL’ cultivar at 0 DAF, 15 seeds at 10 DAF, and 15 seeds at 20 DAF, determined using a principal component analysis.
**Figure S20** Cotyledons at different days after germination (DAG; a) and sample distances (b) of 15 RNA‐seq samples at 1 DAG, 3 DAG, 5 DAG, and 10 DAG, as determined using a principal component analysis.
**Figure S21** Filaments (a) and sample distances (b) of 15 RNA‐seq samples at five different stages: small bud stage (SBS), middle bud stage (MBS), initial flowering stage (IFS), peak flowering stage (PFS), and decayed flowering stage (DFS) during flower development, as determined using a principal component analysis.
**Figure S22** Distribution of *CarFAD2s* in 12 safflower chromosomes.
**Figure S23** Sequence alignment of 5ʹ UTR region (a) and coding sequence (b) of *CarFAD2‐12* in ‘HL’ and ‘LL’ cultivars.
**Figure S24** The phylogenetic tree of *FAD2* (*FATTY ACID* *DESATURASE 2*) genes of eight species, *Arabidopsis thaliana* (At), *Vitis vinifera* (Vit), *Coffea canephora* (Cof), *Cynara cardunculus* (Cyn), *Erigeron breviscapus* (Eri), *Helianthus annuus* (Hel), *Lactuca sativa* (Lac), and *Carthamus tinctorius* (Car), constructed using Mega X with a Neighbor‐Joining method and 500 bootstraps.
**Figure S25** Relationships of the coexpressed modules from 45 RNA‐seq samples in seed development, revealed by the correlation of the module eigengene values (i.e., the first principal component).
**Figure S26** Expression pattern for each gene module from 45 RNA‐seq samples in seed development.
**Figure S27** Associated protein network of *Arabidopsis* homologs of the genes in ‘deeppink’ module including *CarFAD2‐12*, as determined using STRING 11.0.
**Figure S28** Differentially alternatively spliced (AS) genes involved in fatty acid biosynthesis.
**Figure S29** Top 20 GO and KEGG terms associated with the unique differentially expressed genes and differentially alternatively spliced genes in the comparison of seeds at 10 days after flowering (DAF10) versus DAF0.
**Figure S30** Top 20 GO and KEGG terms associated with the unique differentially expressed genes and differentially alternatively spliced genes in the comparison of seeds at 20 days after flowering (DAF20) versus DAF10.
**Figure S31** Top 20 GO and KEGG terms associated with the common differentially expressed genes and differentially alternatively spliced genes in the comparison of seeds at 10 days after flowering (DAF10) versus DAF0.
**Figure S32** Top 20 GO and KEGG terms associated with the common differentially expressed genes and differentially alternatively spliced genes in the comparison of seeds at 10 days after flowering (DAF20) versus DAF10.
**Figure S33** The length distribution of the 52 identified miRNAs in seed formation.
**Figure S34** Enrichment of GO terms in the miRNA‐targeted genes for each cluster with a q‐value < 0.05.
**Figure S54** Expression relationship of Cluster_135896 (ath‐miR156h) and its possible target gene *CarFAD2‐4* throughout seed development.
**Figure S36** Contents of HSYA, rutin, luteolin, and quercetin from five different stages: small bud stage (SBS), middle bud stage (MBS), initial flowering stage (IFS), peak flowering stage (PFS), and decayed flowering stage (DFS) during flower development.
**Figure S37** Schematic diagram of the flavonoid biosynthesis pathway in safflower.
**Figure S38** Gene tree of *CarCHSs* and reported *CtCHS1*, *CtCHS2*, and *CtCHS4* constructed by neighbor‐joining method.
**Figure S39** Distribution of *CarCHSs* in 12 safflower chromosomes.
**Figure S40** Top 20 GO and KEGG terms associated with the unique differentially expressed genes and differentially alternatively spliced genes in the comparison of flowers at the small bud stage (SBS) versus middle bud stage (MBS).
**Figure S41** Top 20 GO and KEGG terms associated with the unique differentially expressed genes and differentially alternatively spliced genes in the comparison of flowers at the middle bud stage (MBS) versus the initial flowering stage (IFS).
**Figure S42** Top 20 GO and KEGG terms associated with the unique differentially expressed genes and differentially alternatively spliced genes in the comparison of flowers at the initial flowering stage (IFS) versus the peak flowering stage (PFS).
**Figure S43** Top 20 GO and KEGG terms associated with the unique differentially expressed genes and differentially alternatively spliced genes in the comparison of flowers at the peak flowering stage (PFS) versus the decayed flowering stage (DFS).
**Figure S44** Top 20 GO and KEGG terms associated with the common differentially expressed genes and differentially alternatively spliced genes in the comparison of flowers at the initial flowering stage (IFS) versus the peak flowering stage (PFS).
**Figure S45** Top 20 GO and KEGG terms associated with the common differentially expressed genes and differentially alternatively spliced genes in the comparison of flowers at the peak flowering stage versus (PFS) the decayed flowering stage (DFS).
**Figure S46** Expression pattern of the 11 genes common in the differentially alternatively spliced of the four groups (SBS‐MBS, MBS‐IFS, IFS‐PFS, and PFS‐DFS).
**Figure S47** Protein sequence alignment of two alternative‐splicing variants of *CarCHS4* in flower development.
**Figure S48** Gene structure of *FAD2* members identified in safflower and sunflower.
**Figure S49***CarCHSs*‐associated coexpression network in flower.

**Table S1** Summary of the PacBio reads for safflower

**Table S2** Statistics of 12 chromosomes (superscaffolds) assembled by long reads assisted with Hi‐C sequencing
**Table S4** BUSCO results for the safflower genome
**Table S7** Genomes of the representative plant species used in the phylogenomic and comparative genomics analyses

**Table S3** Statistics of the mapping ratios of RNA‐seq data from 15 seedling samples, 15 flower samples, 45 ‘HL’ seed samples planted in house, 6 ‘HL’ seeds and 6 ‘LL’ seed samples planted in field

**Table S5** Identification and classification of the repeat element in the safflower genome

**Table S6** Transcriptional factors, transcriptional regulators, and protein kinases in the genomes of safflower and nine other species

**Table S8** Enrichment of the GO terms and KEGG pathways for the gene families specific to safflower

**Table S9** Enrichment of the GO terms and KEGG pathways for the gene families specifically expanded and contracted in safflower genome compared to other genomes

**Table S10** Identification and classification of the gene families involved in the biosynthesis of linoleic acid and oleic acid

**Table S11** Expression pattern of the gene families involved in the biosynthesis of linoleic acid and oleic acid

**Table S12** The gene symbols, identifiers, and protein sequences of *FAD2* (*FATTY ACID* *DESATURASE 2*) gene family identified from the eight species, *Arabidopsis thaliana*, *Vitis vinifera*, *Coffea canephora*, *Cynara cardunculus*, *Erigeron breviscapus*, *Helianthus annuus*, *Lactuca sativa*, and *Carthamus tinctorius*

**Table S13** Assigned modules for all expressed genes in the network, the annotation of ‘deeppink’ module containing *CarFAD2‐12*, and the ‘coral3’ module containing other *CarFAD2* members

**Table S14** Differentially expressed genes (DEGs) and differentially alternatively spliced genes (DASGs) in seed development

**Table S15** GO enrichment analysis of the differentially expressed genes (DEGs) and differentially alternatively spliced genes (DASGs) in seed development

**Table S16** Expression pattern of the miRNAs and annotation of their targeted genes

**Table S17** Identification, classification, and expression of gene families and gene clusters involved flavonoid biosynthesis
