# Supplementary material for: The chromosome‐scale reference genome of safflower (Carthamus tinctorius) provides insights into linoleic acid and flavonoid biosynthesis
Source: Plant Biotechnol J. 2021 Apr 8;19(9):1725–42. doi: 10.1111/pbi.13586 (PMC8428823; doi:10.1111/pbi.13586)
Supplement: Supplementary file 1 — Figure S1 Evaluation of safflower (Carthamus tinctorius) genome size estimated using a k‐mer frequency analysis (a) and flow cytometry using soybean (Glycine max) as a control (b). Figure S2 The Hi‐C interacted heatmap for chromosome‐scale genome assembly. Figure S3 The high‐density genetic linkage map of safflower constructed from the F2 population of a cross between the parents AH04 and YH04. Figure S4 Synteny plot between our assembled safflower genome using Pacbio and Hi‐C, and the published draft genome of safflower generated using Illumina Hi‐Seq. Figure S5 Characteristics of the repetitive elements in the safflower genome. Figure S6 Identification and classification of long noncoding RNAs according to their position in the safflower genome. Figure S7 Gene Ontology categories associated with the annotated genes in the safflower genome. Figure S8 The number of transcriptional factors (TFs), transcriptional regulators (TRs), and protein kinases (PKs) in the safflower (Carthamus tinctorius) and nine other plant genomes. Figure S9 Proportions of transcriptional factors (TFs), transcriptional regulators (TRs), and protein kinases (PKs) in the safflower and nine other plant genomes. Figure S10 Types of gene duplication in the safflower (Carthamus tinctorius) genome and five other plant species. Figure S11 Syntenic depths in the artichoke versus safflower genome comparison. Figure S12 Enrichment of biological process GO terms (a) and KEGG pathways (b) associated with the gene families specific to safflower with a q‐value <0.05. Figure S13 Enrichment of biological process GO terms (a) and KEGG pathways (b) associated with the expanded gene families in safflower with a q‐value <0.05. Figure S14 Enrichment of biological process GO terms (a) and KEGG pathways (b) associated with the contracted gene families in safflower with q‐value <0.05. Figure S15 Safflower seed oil content and fatty acid composition of ‘HL’ (high linoleic acid) and ‘LL’ (low linoleic acid) cultivar p [file PBI-19-1725-s016.pdf]

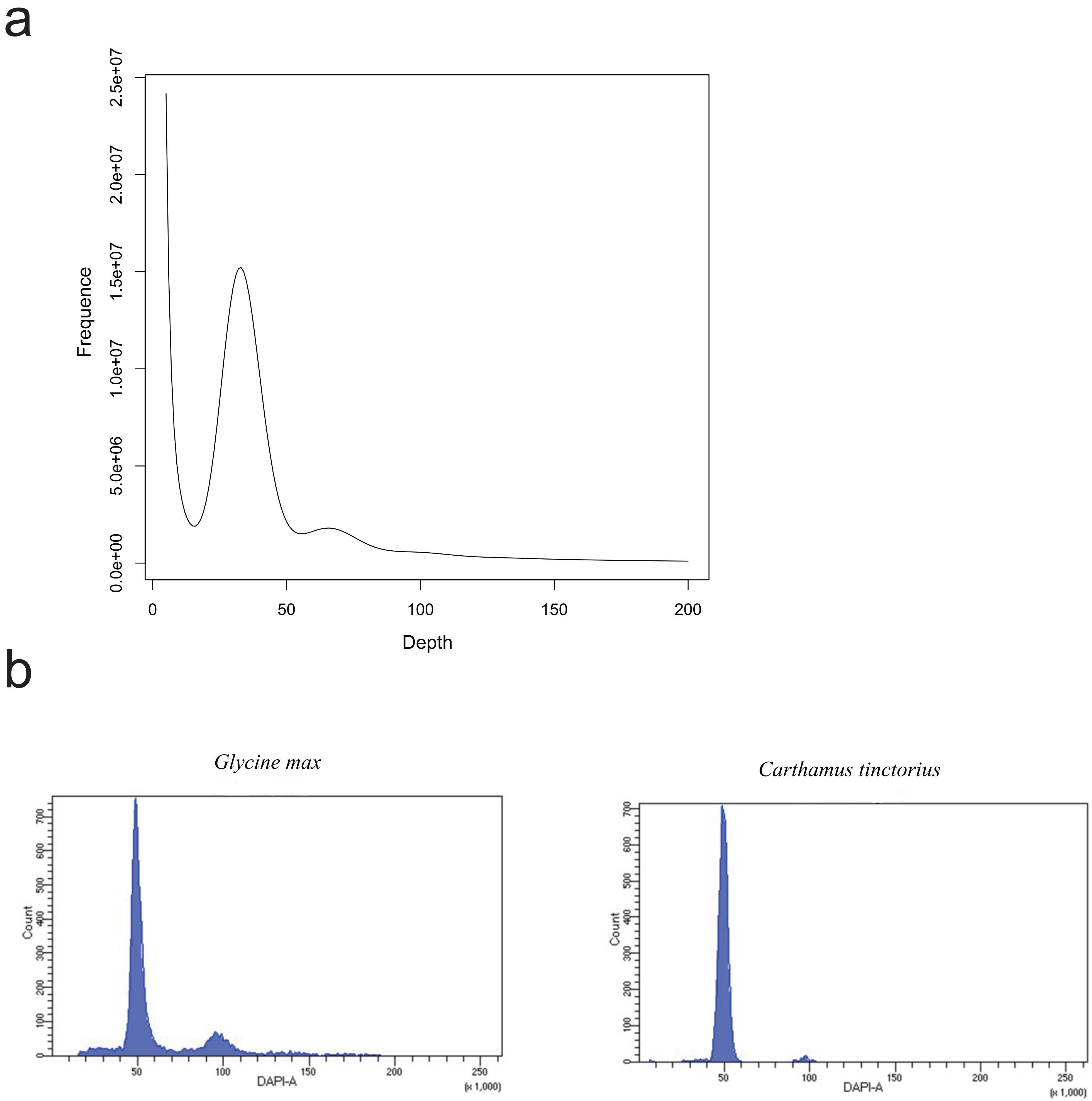

Fig. S1 Evaluation of safflower (*Carthamus tinctorius*) genome size estimated using a k-mer frequency analysis **(a)** and flow cytometry using soybean (*Glycine max*) as a control **(b)**. In **(a)**, one main peak was present at a depth of 33, with a minor peak at a depth of 66 where k = 17.

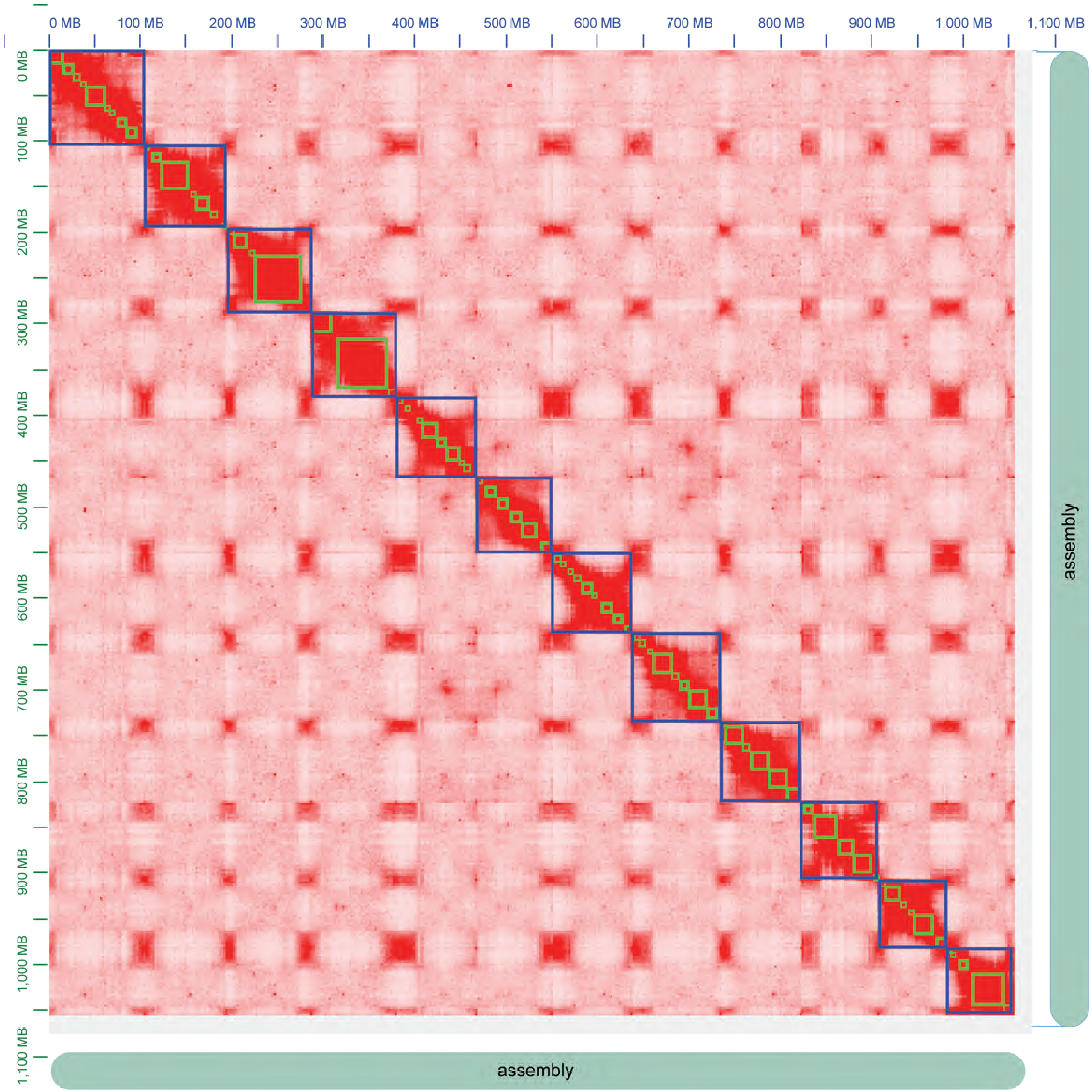

Fig. S2 The Hi-C interacted heatmap for chromosome-scale genome assembly.

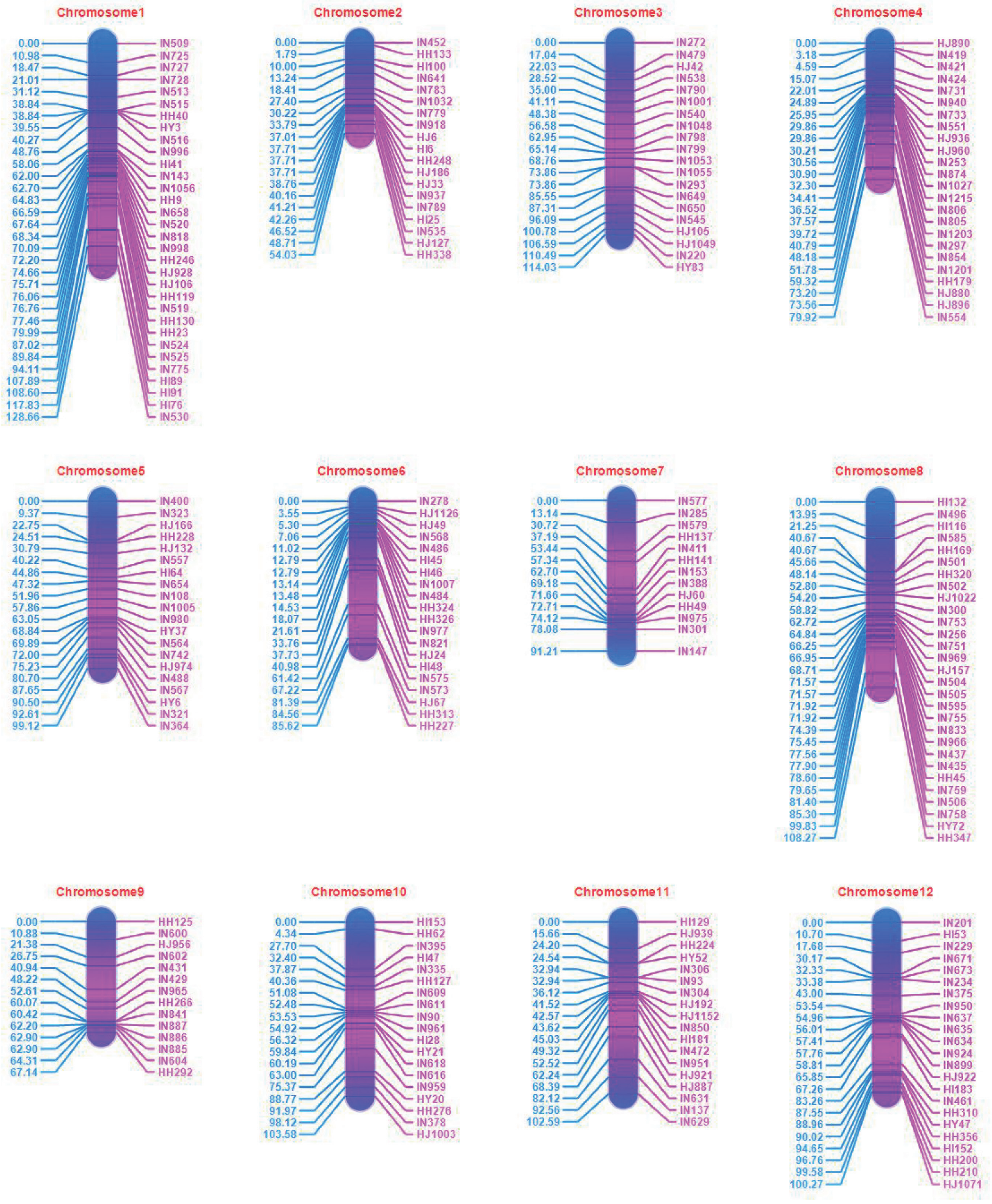

Fig. S3 The high-density genetic linkage map of safflower constructed from the F_2 population of a cross between the parents AH04 and YH04.

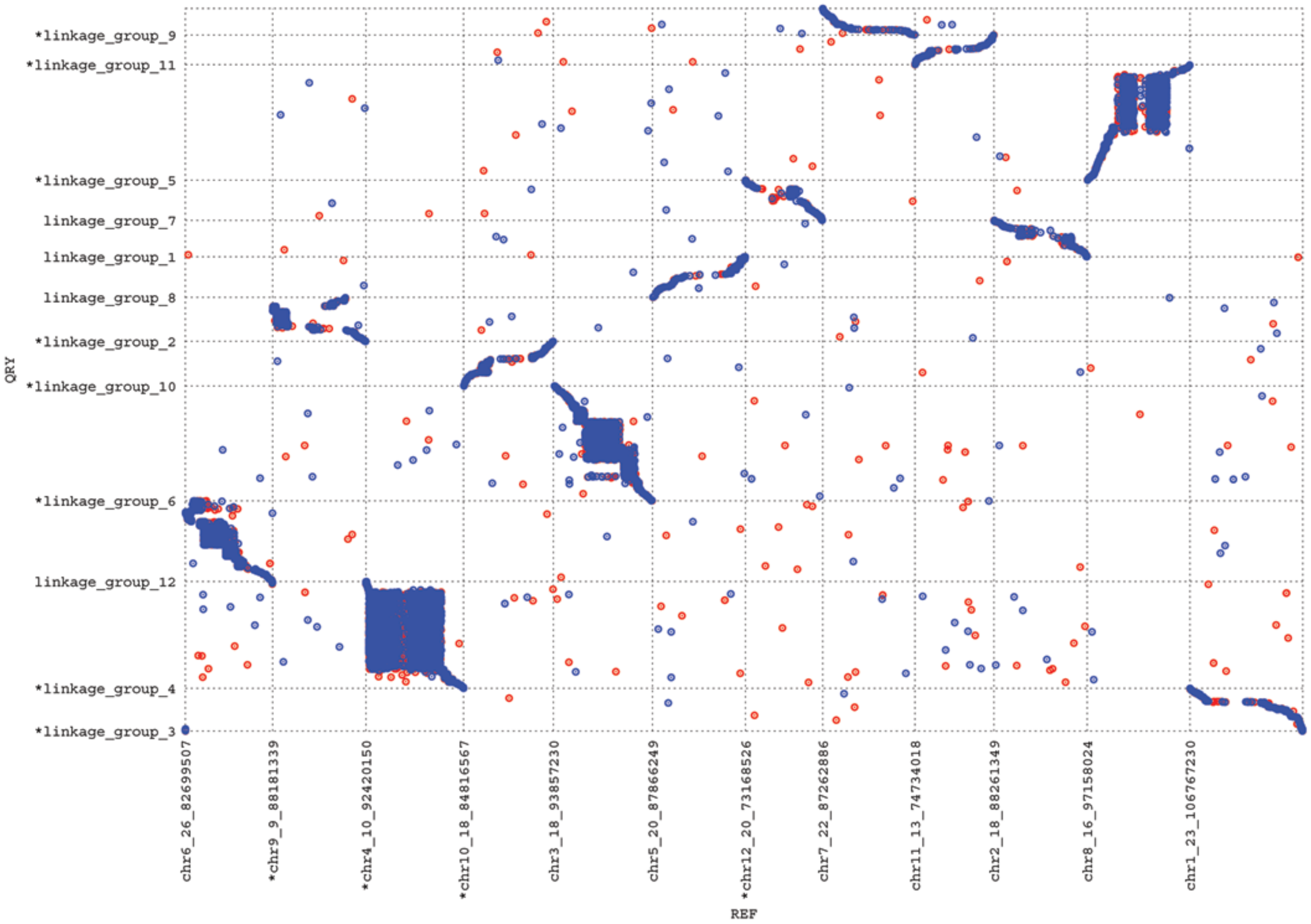

Fig. S4 Synteny plot between our assembled safflower genome using Pacbio and Hi-C, and the published draft genome of safflower generated using Illumina Hi-Seq. The x-axis and y-axis represent the 12 published linkage groups and 12 assembled chromosomes in the study, respectively. Each square represents the region for pairwise synteny among chromosomes and linkage groups. Red dots and blue dots represent reverse mapping sequences and forward mapping sequences between published and our genomes, respectively.

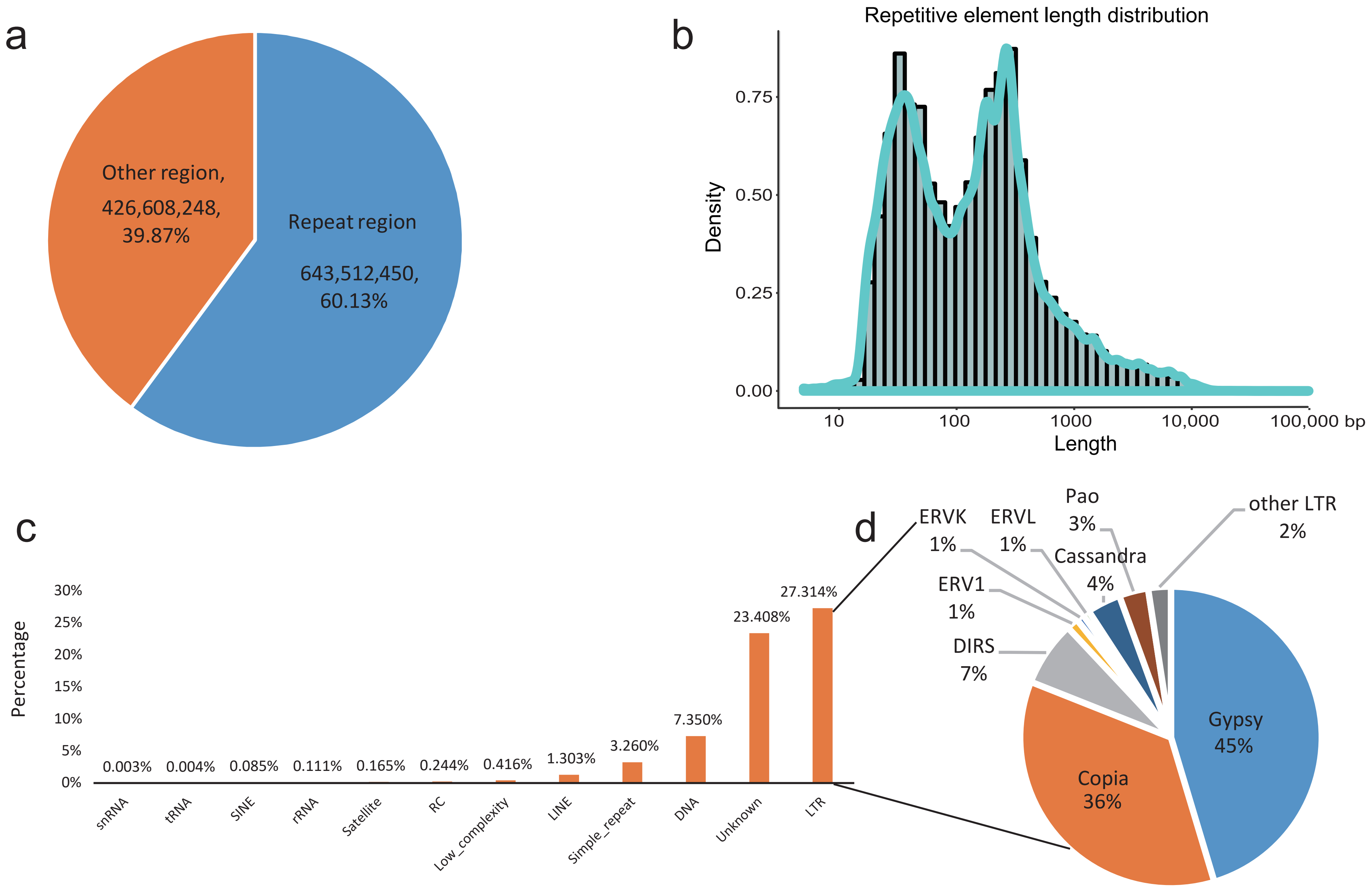

Fig. S5 Characteristics of the repetitive elements in the safflower genome. **(a)** The proportions of repetitive elements in the safflower genome. **(b)** The length distribution of repetitive elements. **(c)** The proportions of different classes of repetitive elements in the safflower genome. **(d)** The proportions of different LTR species.

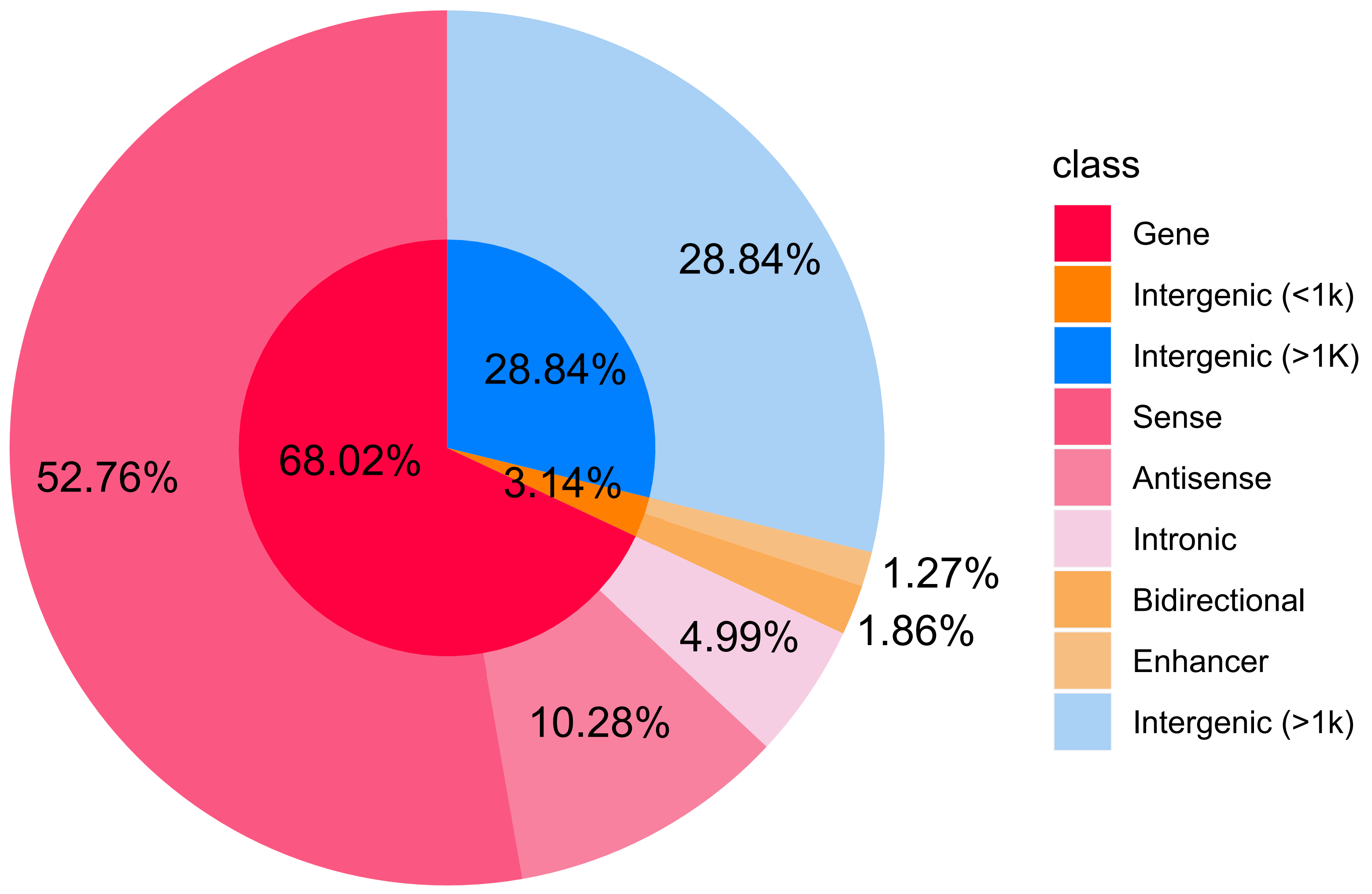

Fig. S6 Identification and classification of long noncoding RNAs according to their position in the safflower genome.

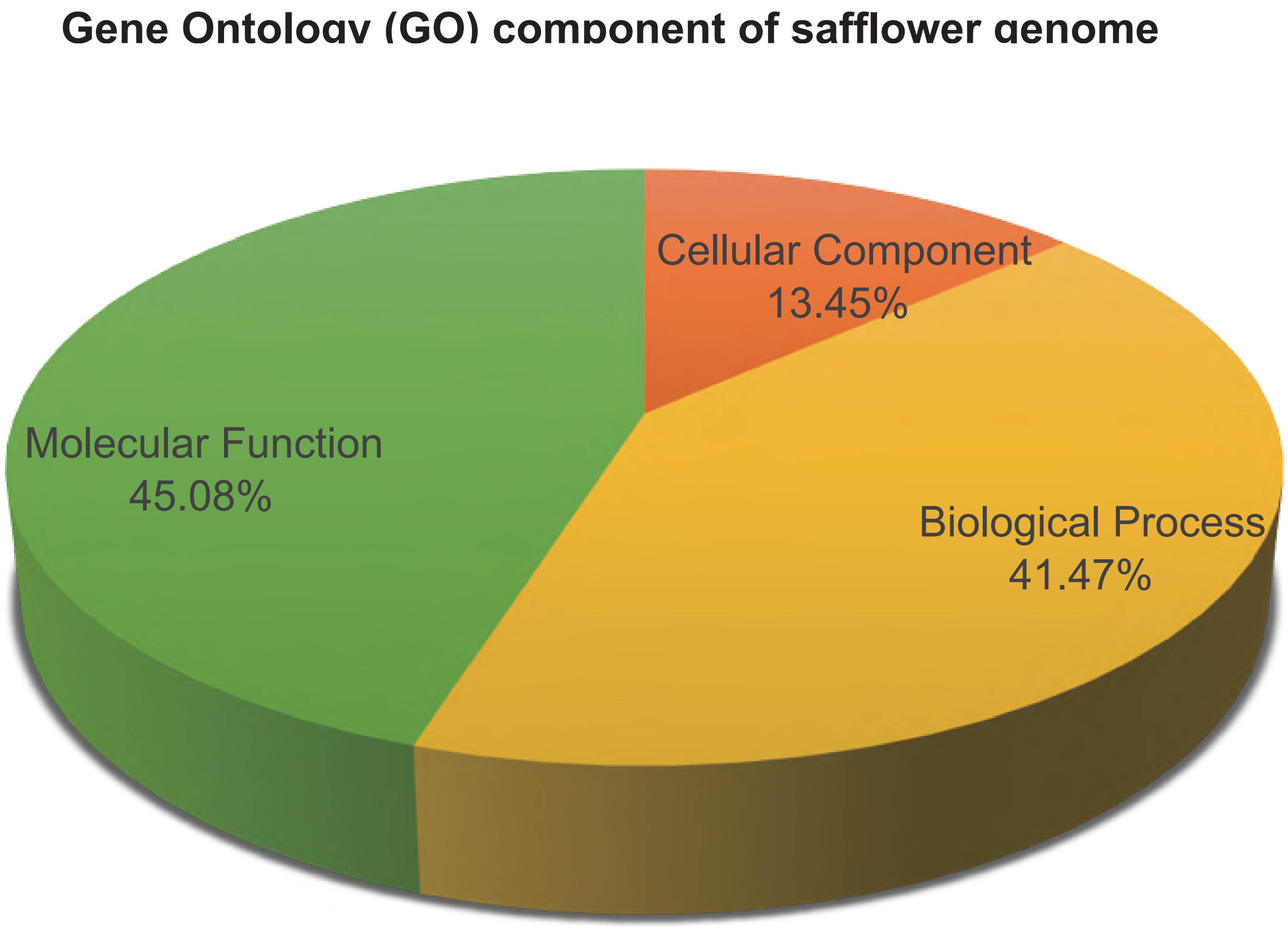

Fig. S7 Gene Ontology categories associated with the annotated genes in the safflower genome.

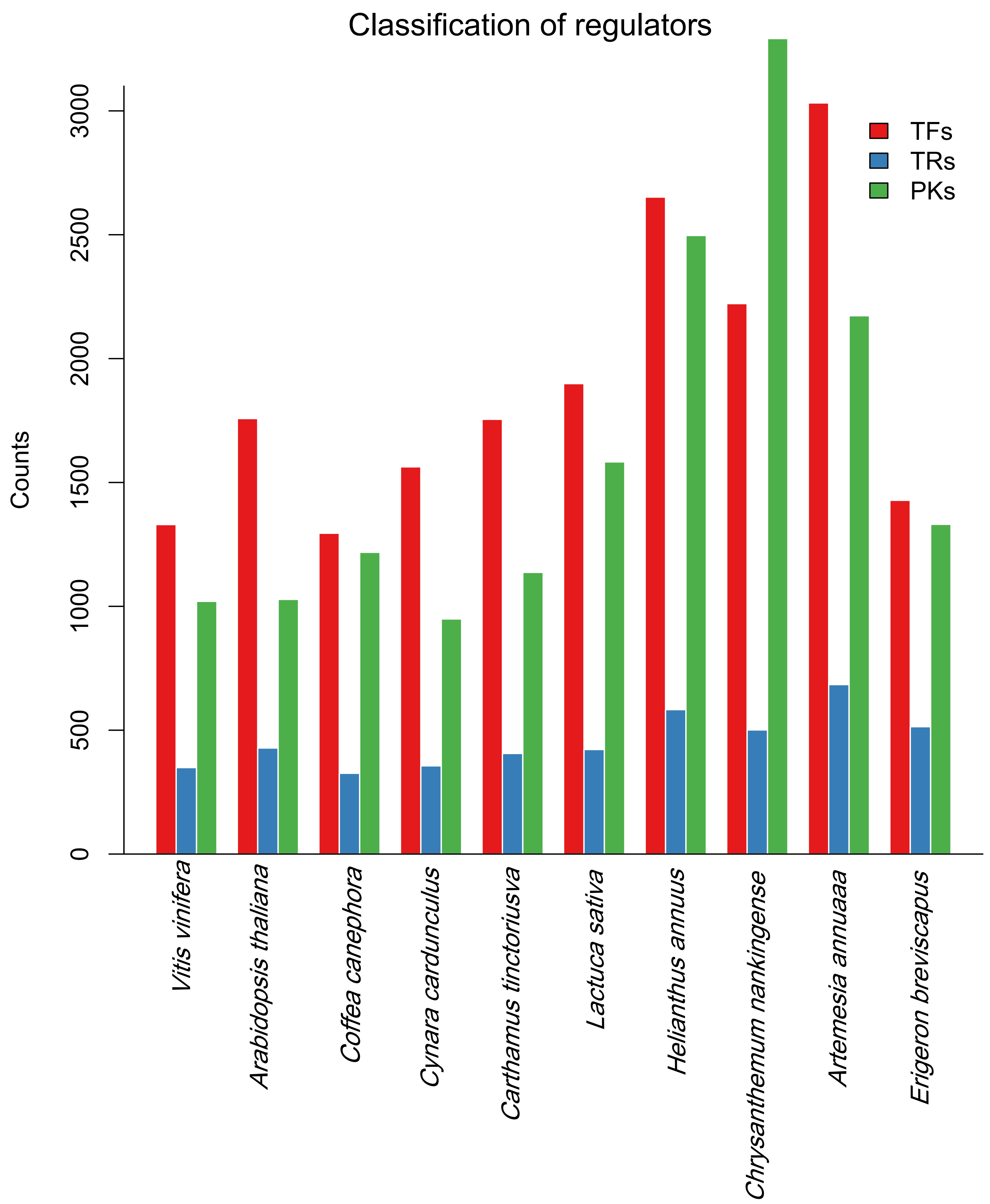

Fig. S8 The number of transcriptional factors (TFs), transcriptional regulators (TRs), and protein kinases (PKs) in the safflower (*Carthamus tinctorius*) and nine other plant genomes.

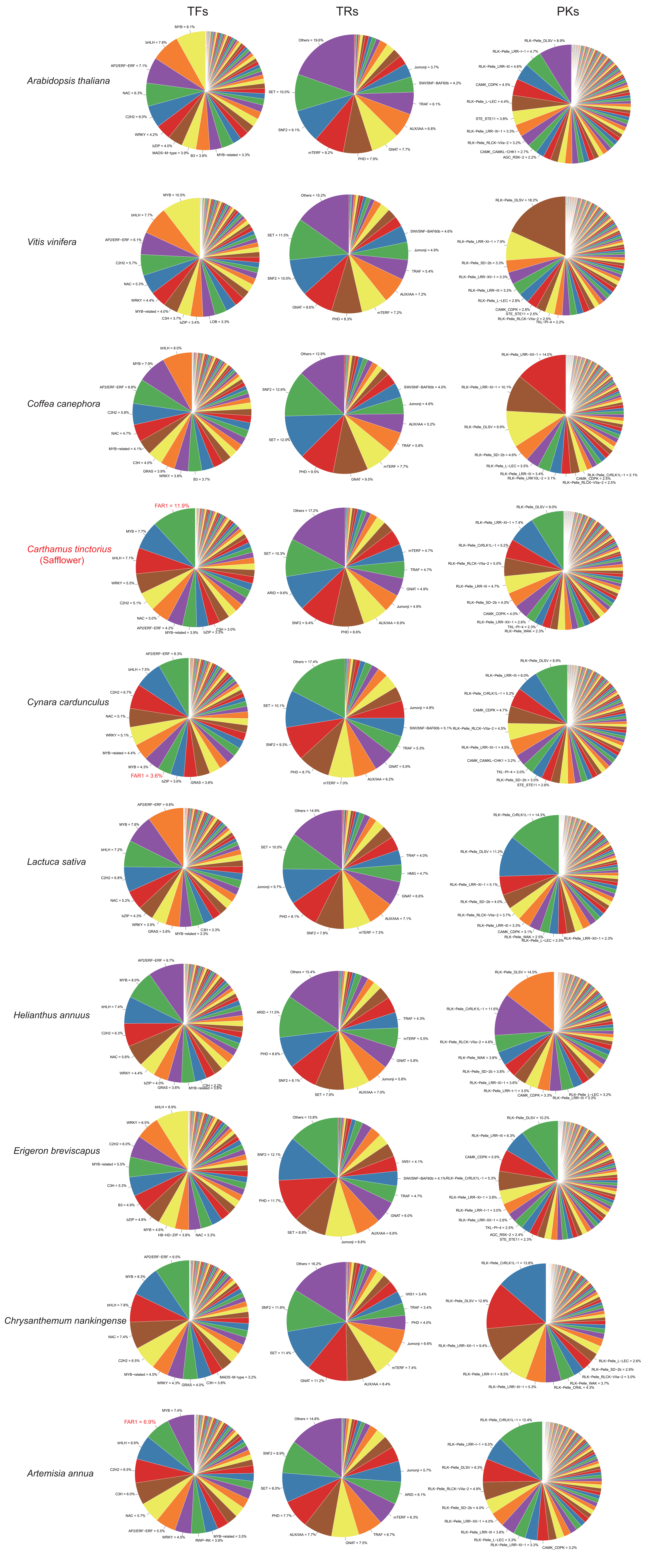

Fig. S9 Proportions of transcriptional factors (TFs), transcriptional regulators (TRs), and protein kinases (PKs) in the safflower and nine other plant genomes.

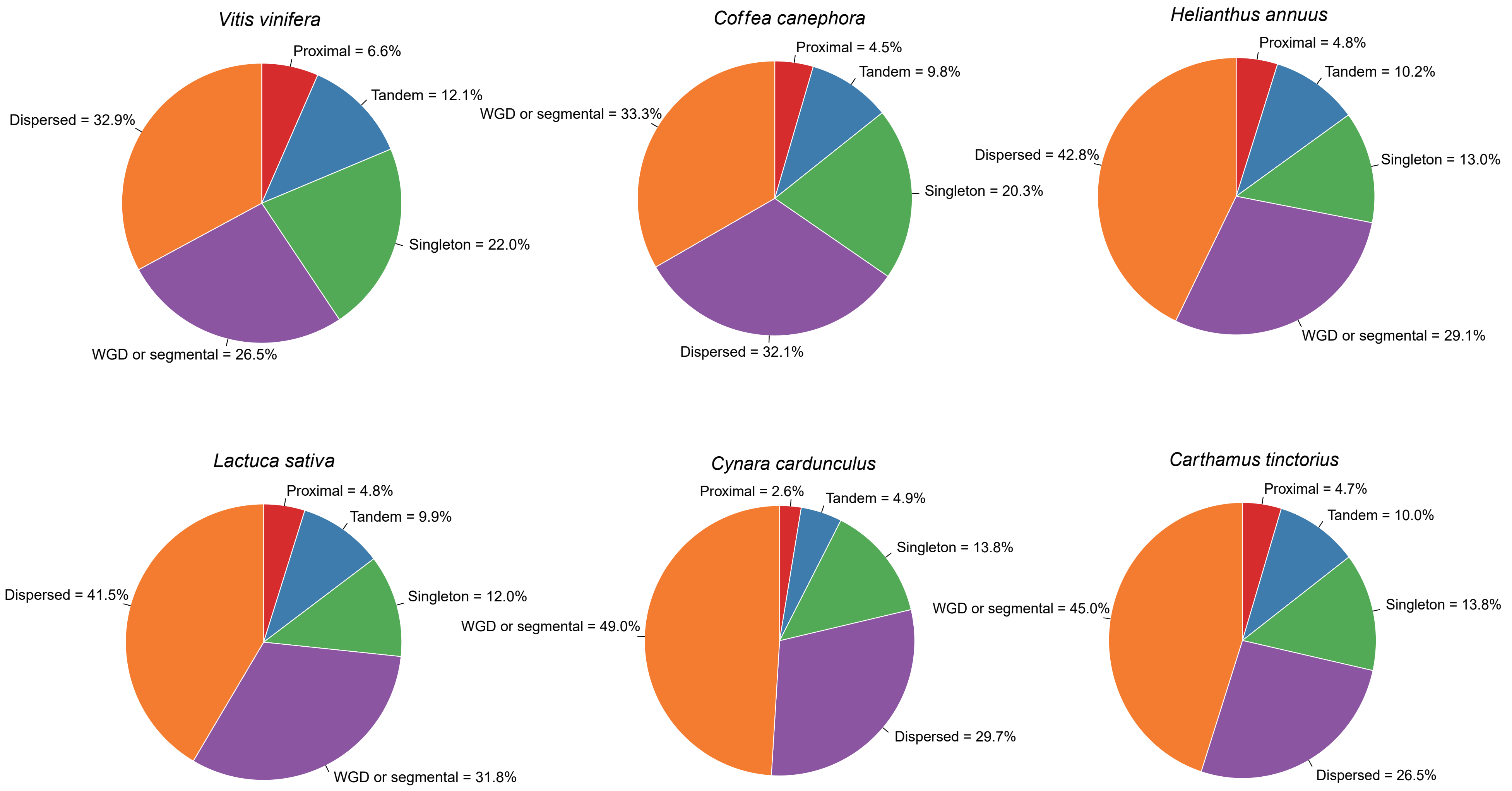

Fig. S10 Types of gene duplication in the safflower (*Carthamus tinctorius*) genome and five other plant species. The distribution of different duplication types was classified using MCScanX as follows: Singleton: no duplication; WGD/segmental: whole-genome or segmental duplications (collinear genes in collinear blocks); Tandem: consecutive duplication; Proximal: duplications in a nearby but not adjacent chromosomal region; Dispersed: duplications using modes other than tandem, proximal, or WGD/segmental. *Vitis vinifera*: grape; *Coffea canephora*: robusta coffee; *Helianthus annuus*: sunflower; *Lactuca sativa*: lettuce; *Cynara cardunculus*: artichoke.

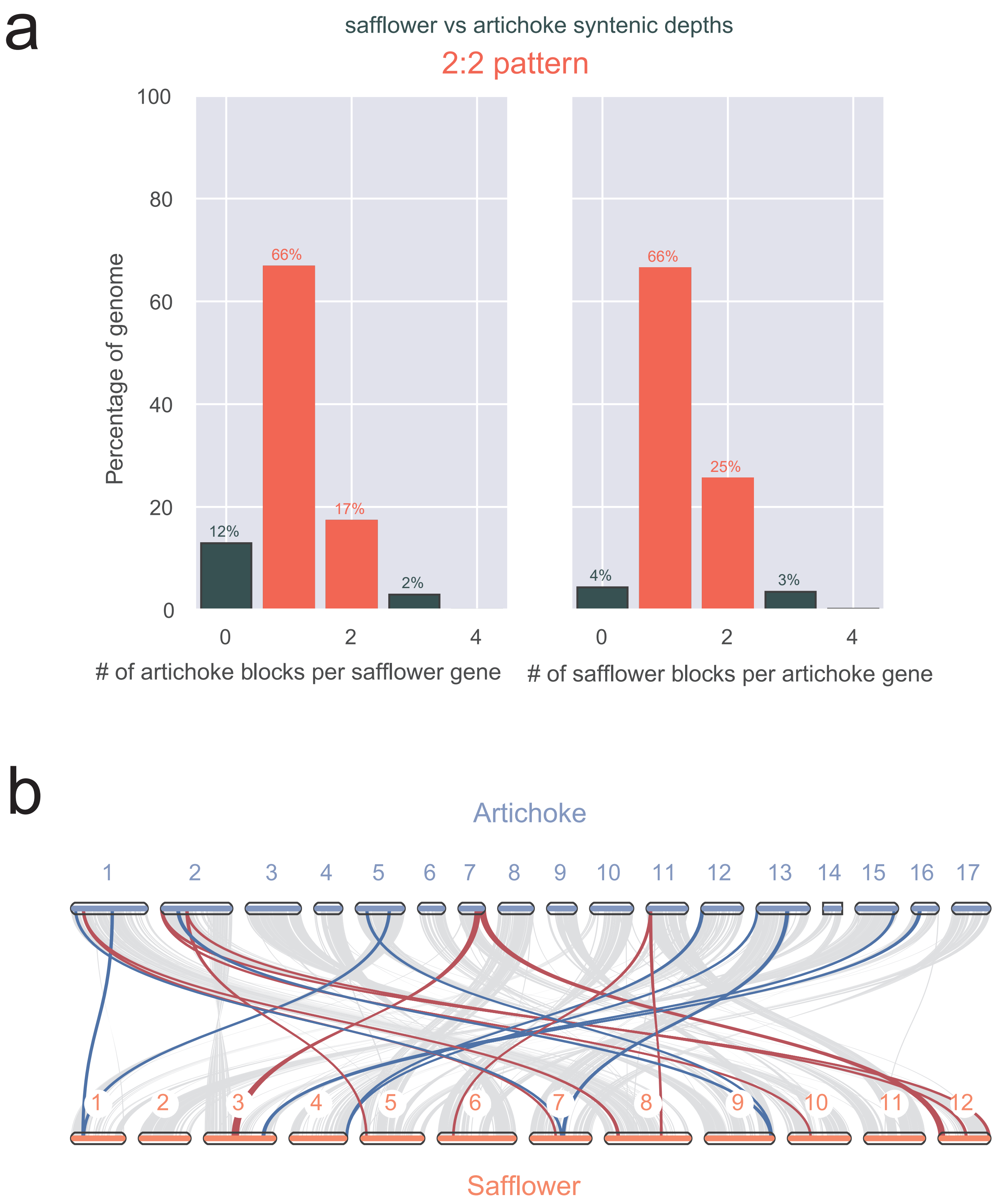

Fig. S11 Syntenic depths in the artichoke versus safflower genome comparison. **(a)** There are two synteny blocks for both artichoke and safflower, indicating they underwent two common WGD events. **(b)** Macrosynteny between the artichoke and safflower karyotypes. Red lines highlight the two copies of artichoke syntenic blocks per corresponding safflower block. Blue lines highlight the two copies of safflower syntenic block per corresponding artichoke block.

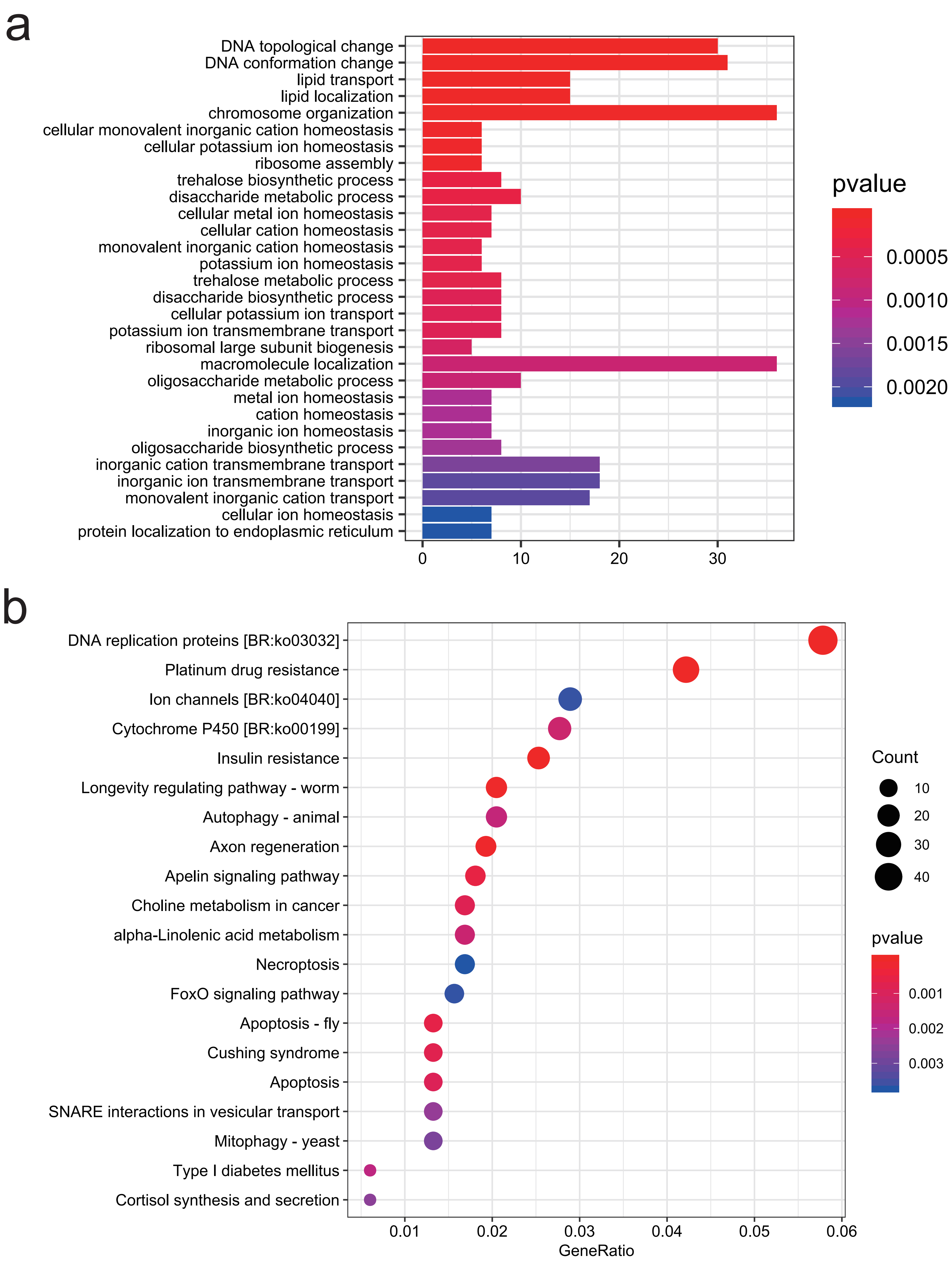

Fig. S12 Enrichment of biological process GO terms **(a)** and KEGG pathways **(b)** associated with the gene families specific to safflower with a q-value <0.05.

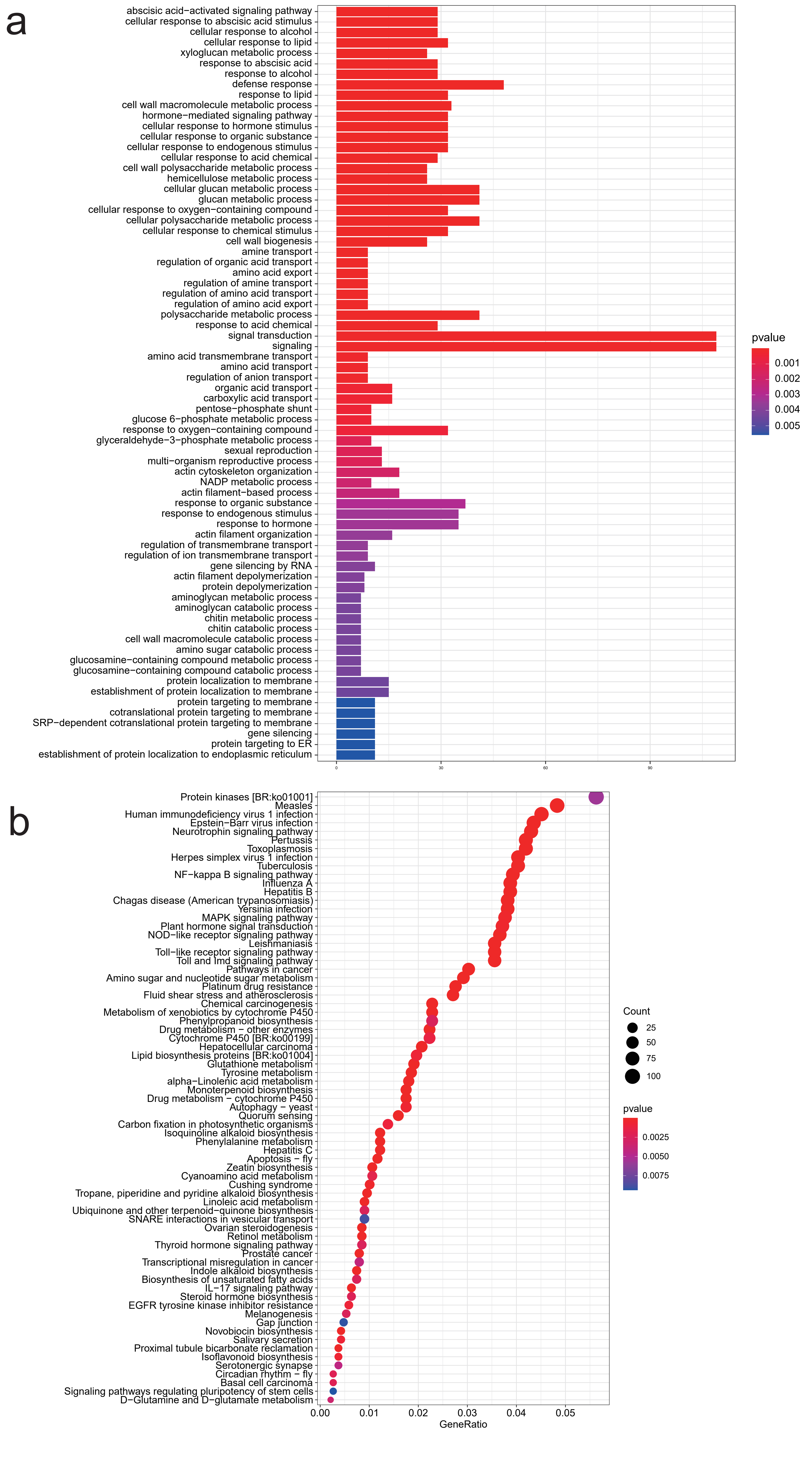

Fig. S13 Enrichment of biological process GO terms **(a)** and KEGG pathways **(b)** associated with the expanded gene families in safflower with a q-value <0.05.

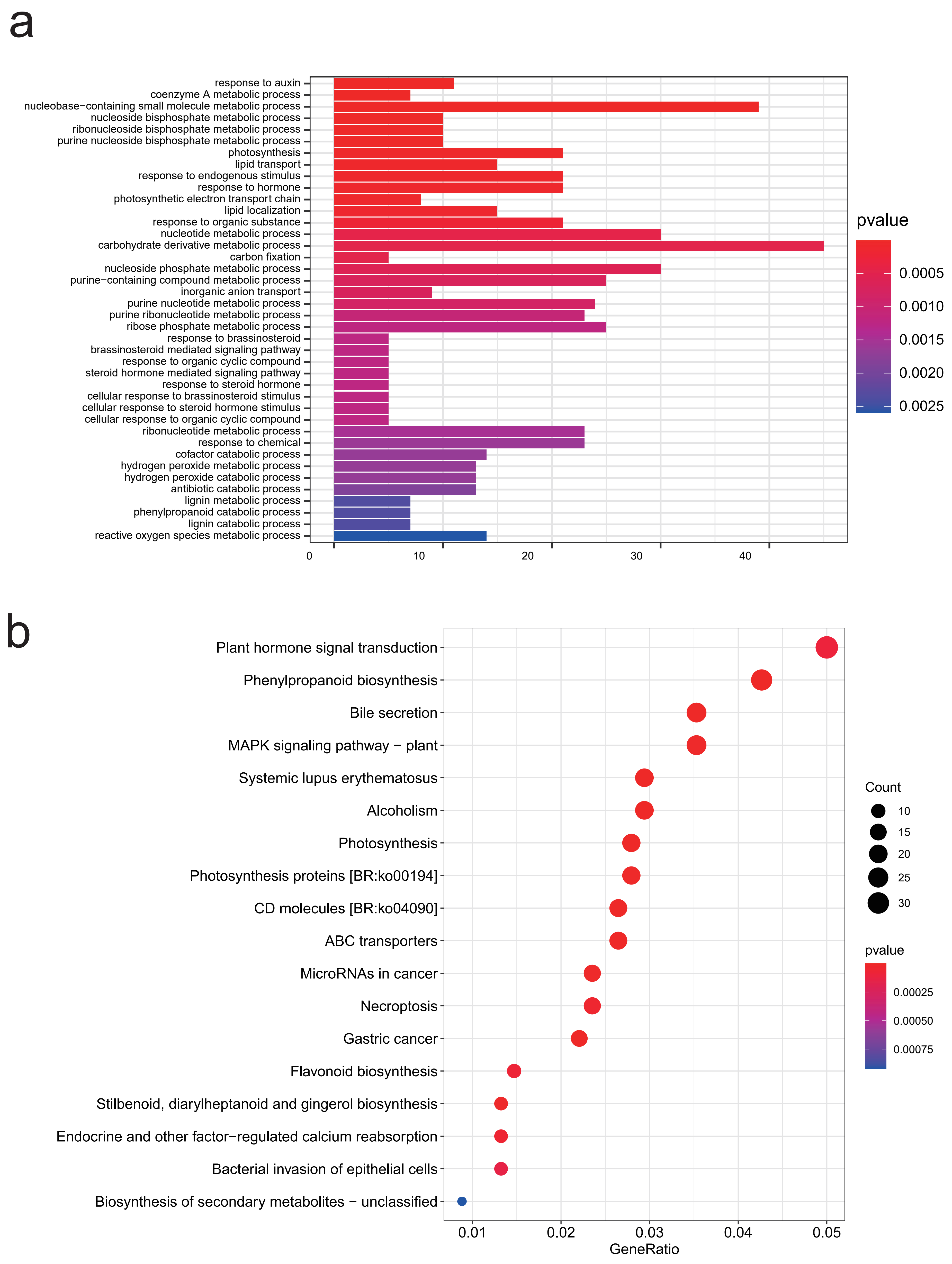

Fig. S14 Enrichment of biological process GO terms **(a)** and KEGG pathways **(b)** associated with the contracted gene families in safflower with q-value <0.05.

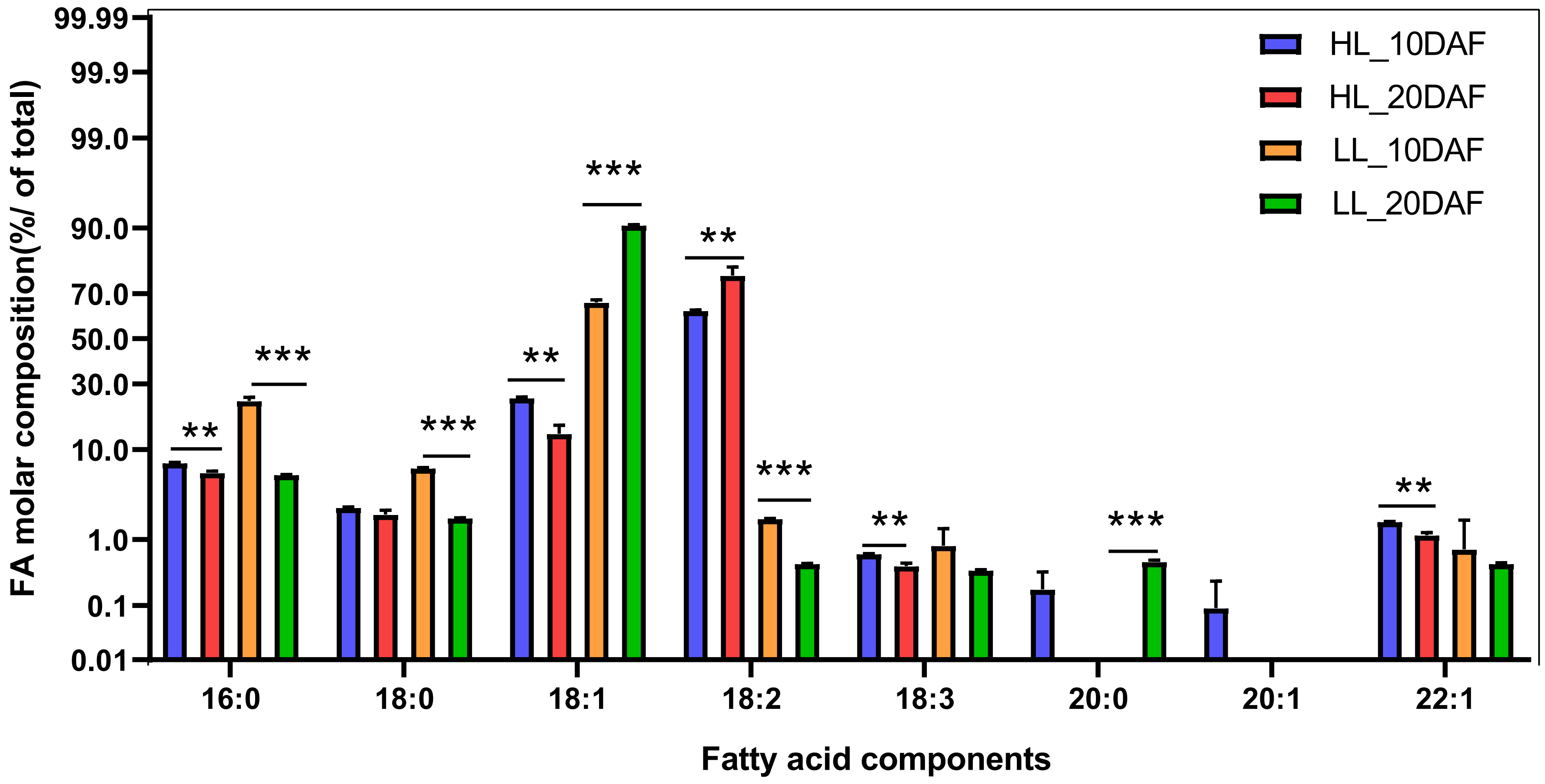

Fig. S15 Safflower seed oil content and fatty acid composition of 'HL' (high linoleic acid) and 'LL' (low linoleic acid) cultivar plants. DAF: day after flowering. Total oil is analyzed using gas chromatography. Values are means ± SD from three independent experiments. Student's t-test: *, $P < 0.05$; **, $P < 0.01$; ***, $P < 0.001$.

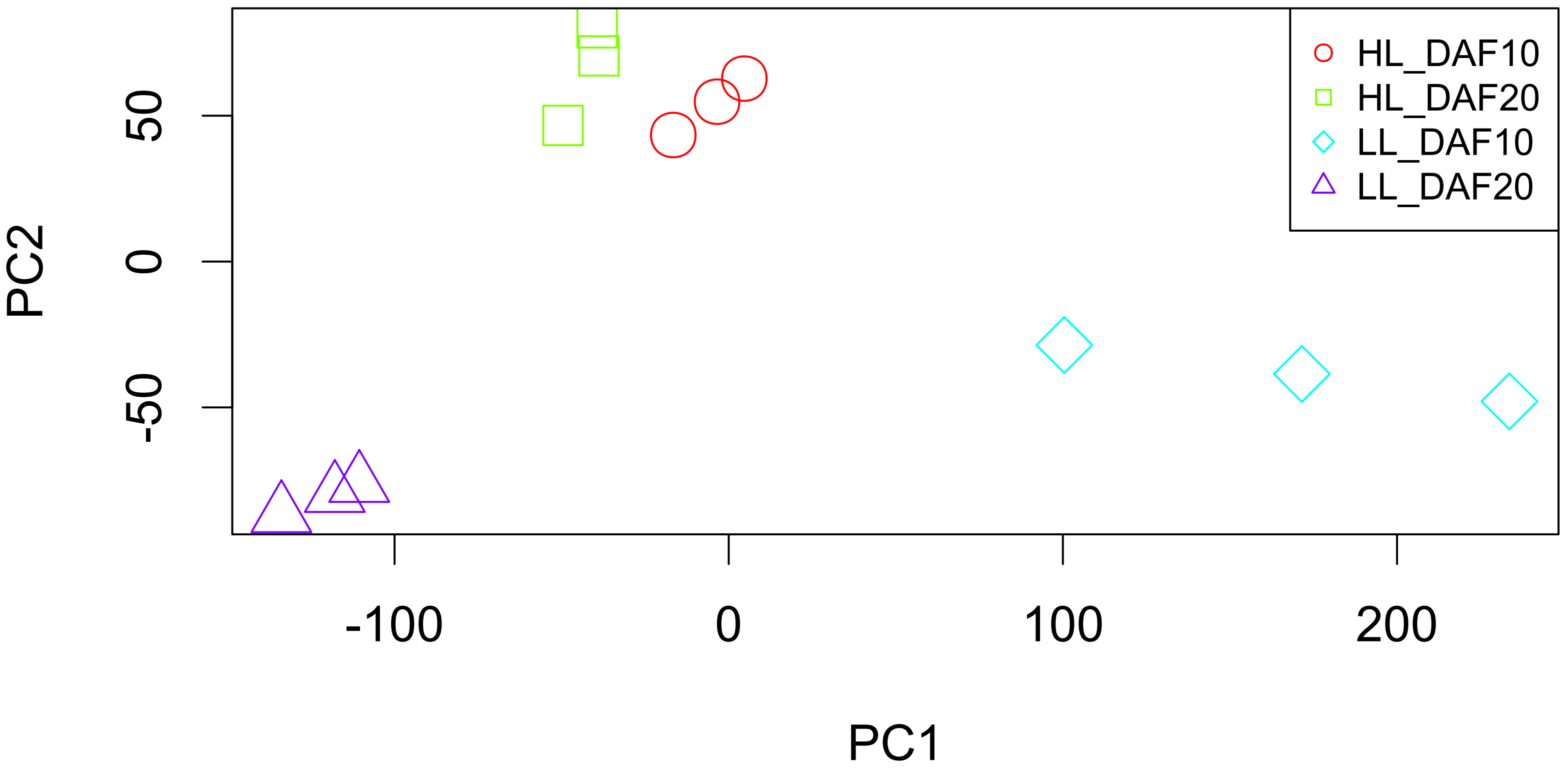

Fig. S16 Sample distance of 12 RNA-seq samples of ‘HL’ (high linoleic acid) and ‘LL’ (low linoleic acid) cultivar seeds at 10 days after flowering (DAF) and 20 DAF. The samples are analyzed using a principal component analysis. DAF: days after flowering.

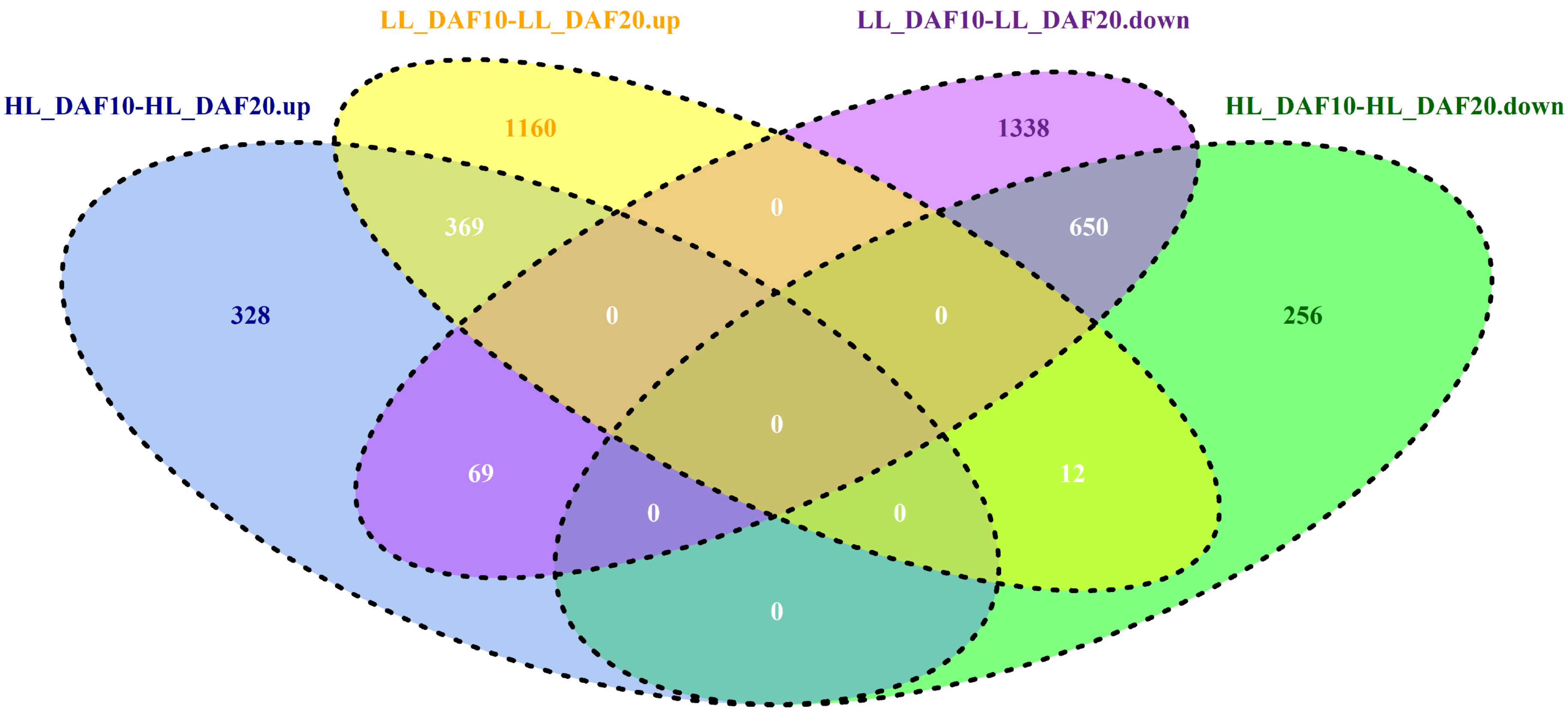

Fig. S17 Venn diagram of four sets of differentially upregulated and downregulated genes in DAF20 versus DAF10 of 'HL' and 'LL' cultivar.

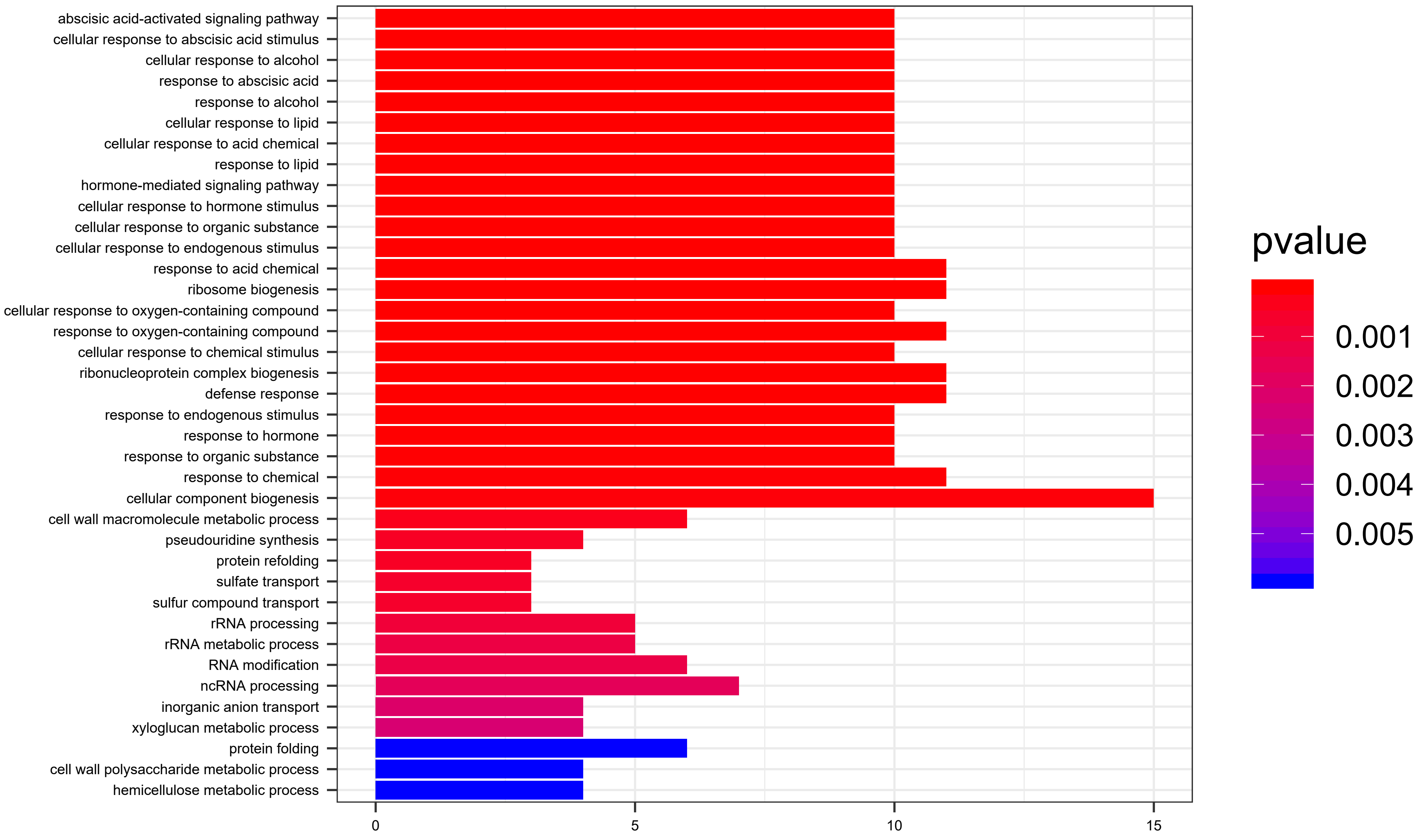

Fig. S18 Enrichment of biological process GO terms of 328 uniquely upregulated genes in DAF20 versus DAF10 in the 'HL' cultivar compared with 'LL' cultivar.

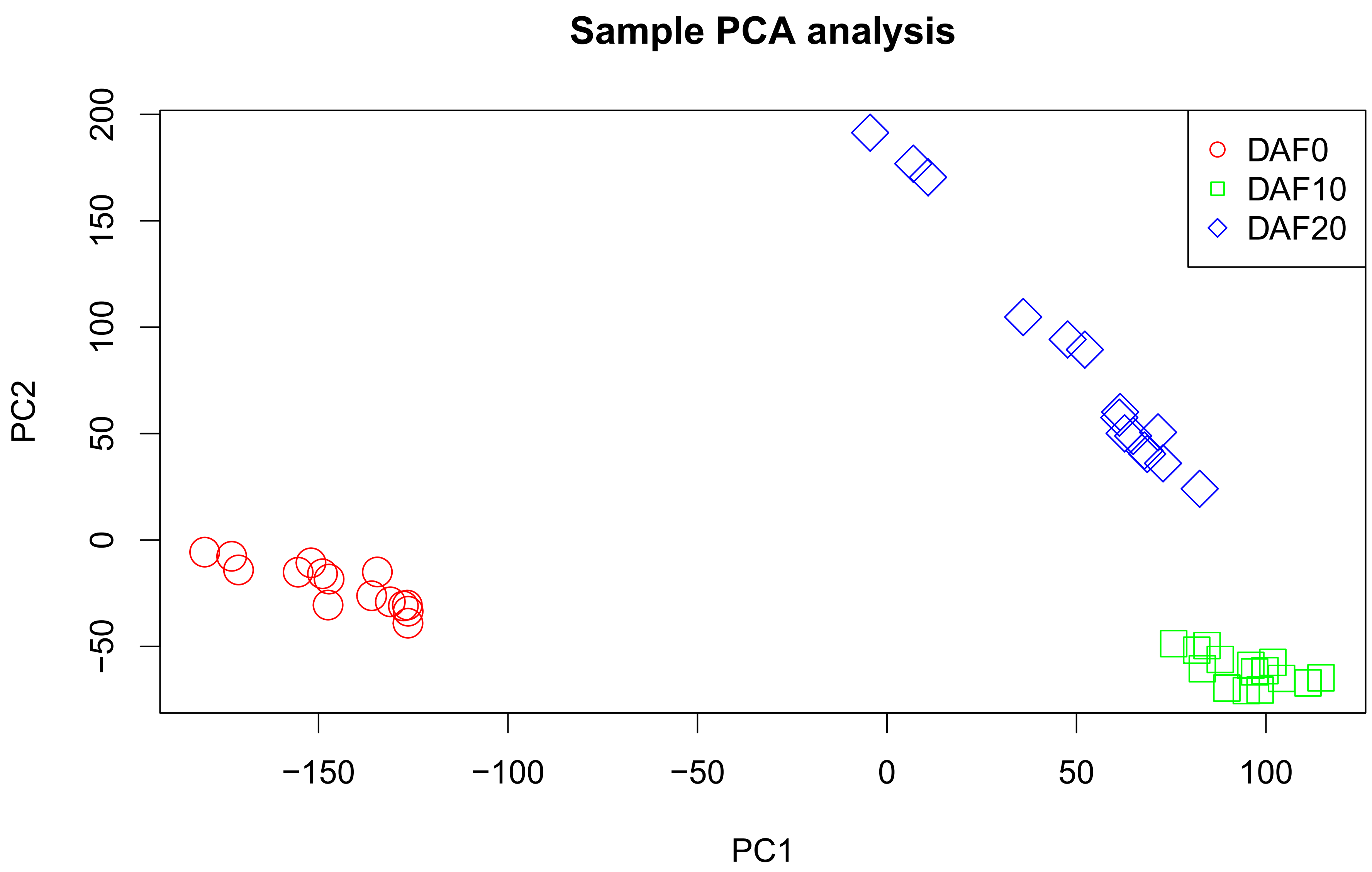

Fig. S19 Sample distance of 45 RNA-seq samples, including 15 ovaries from the 'HL' cultivar at 0 DAF, 15 seeds at 10 DAF, and 15 seeds at 20 DAF, determined using a principal component analysis.

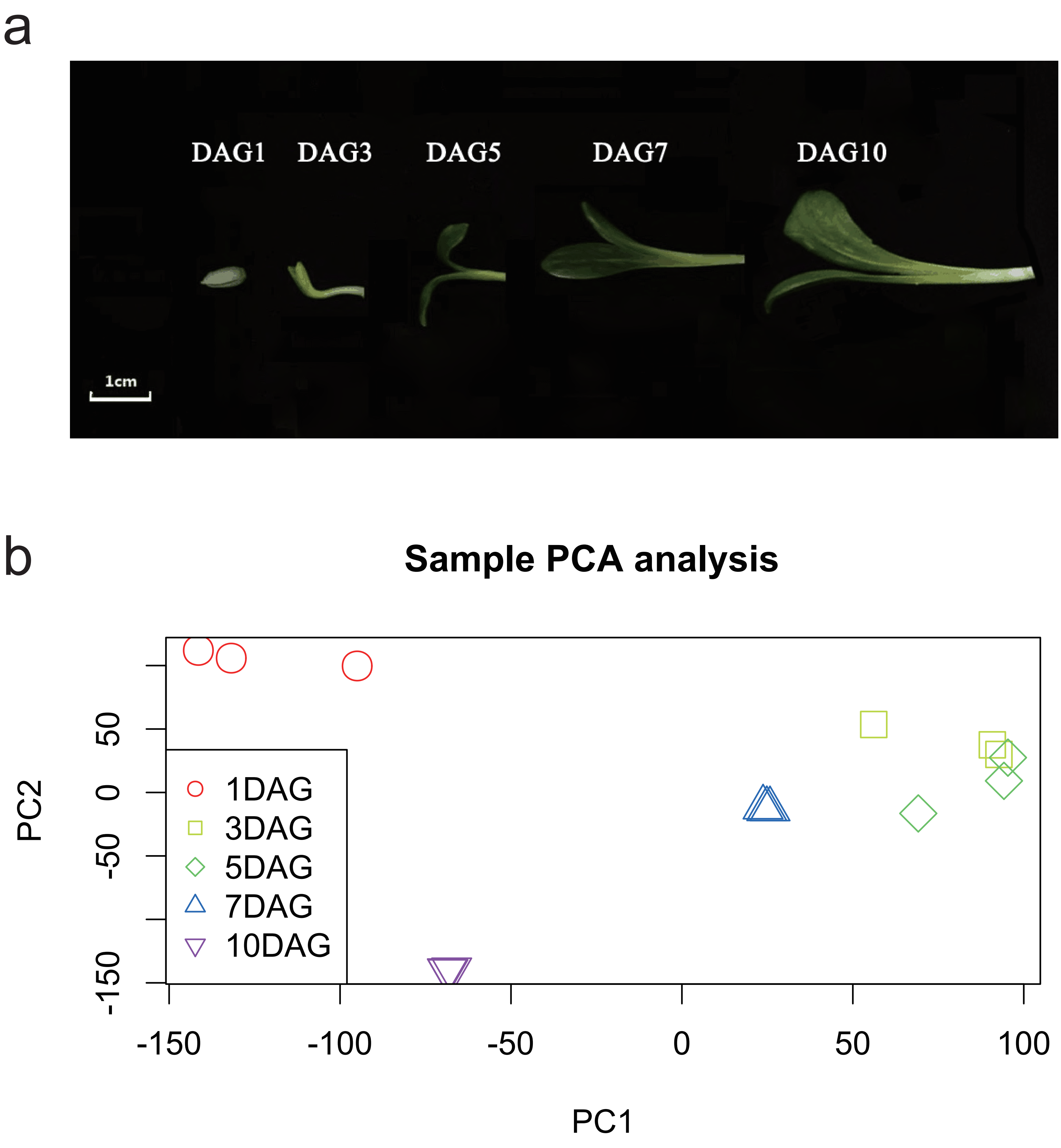

Fig. S20 Cotyledons at different days after germination (DAG; **a**) and sample distances **(b)** of 15 RNA-seq samples at 1 DAG, 3 DAG, 5 DAG, and 10 DAG, as determined using a principal component analysis.

a

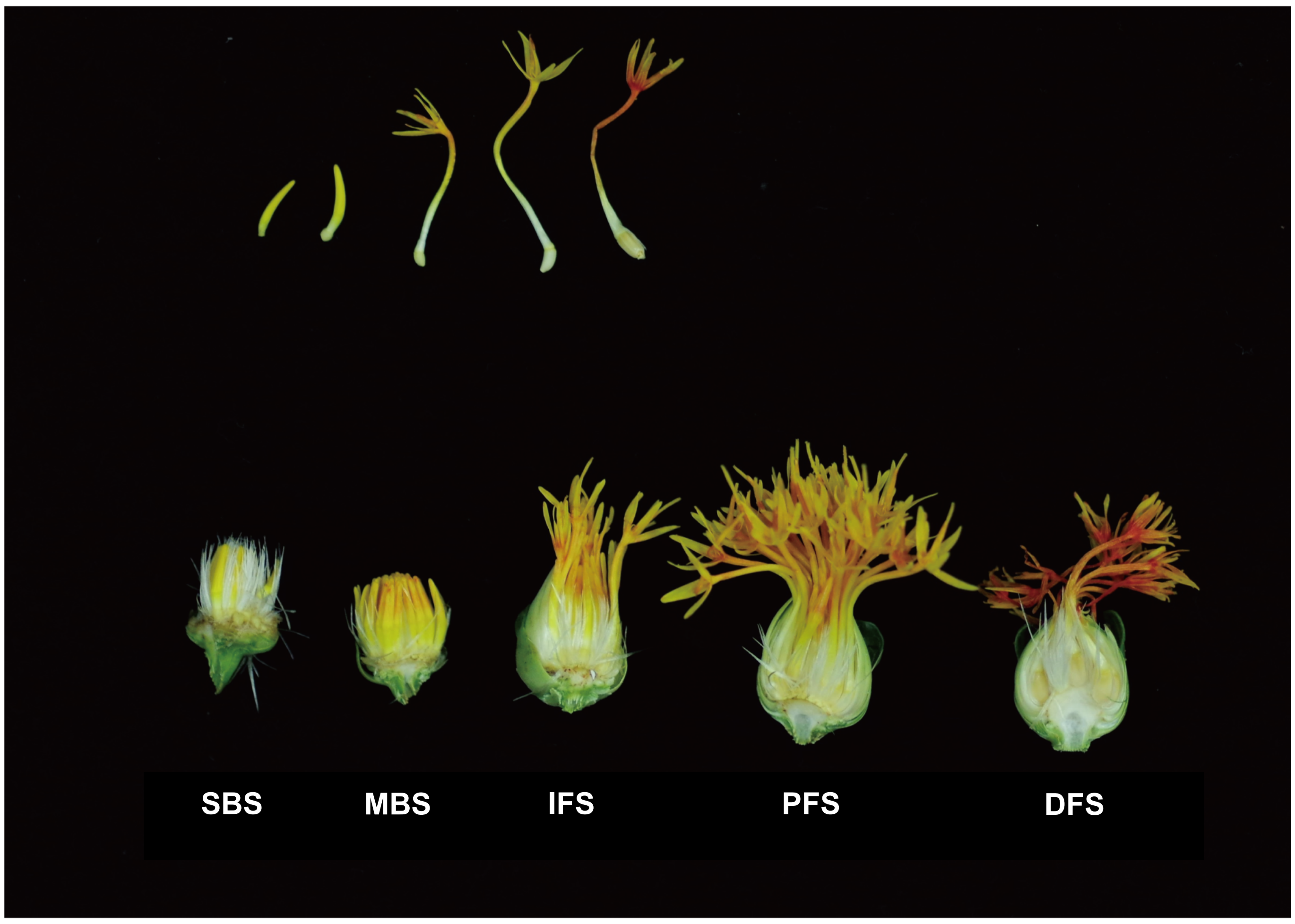

b

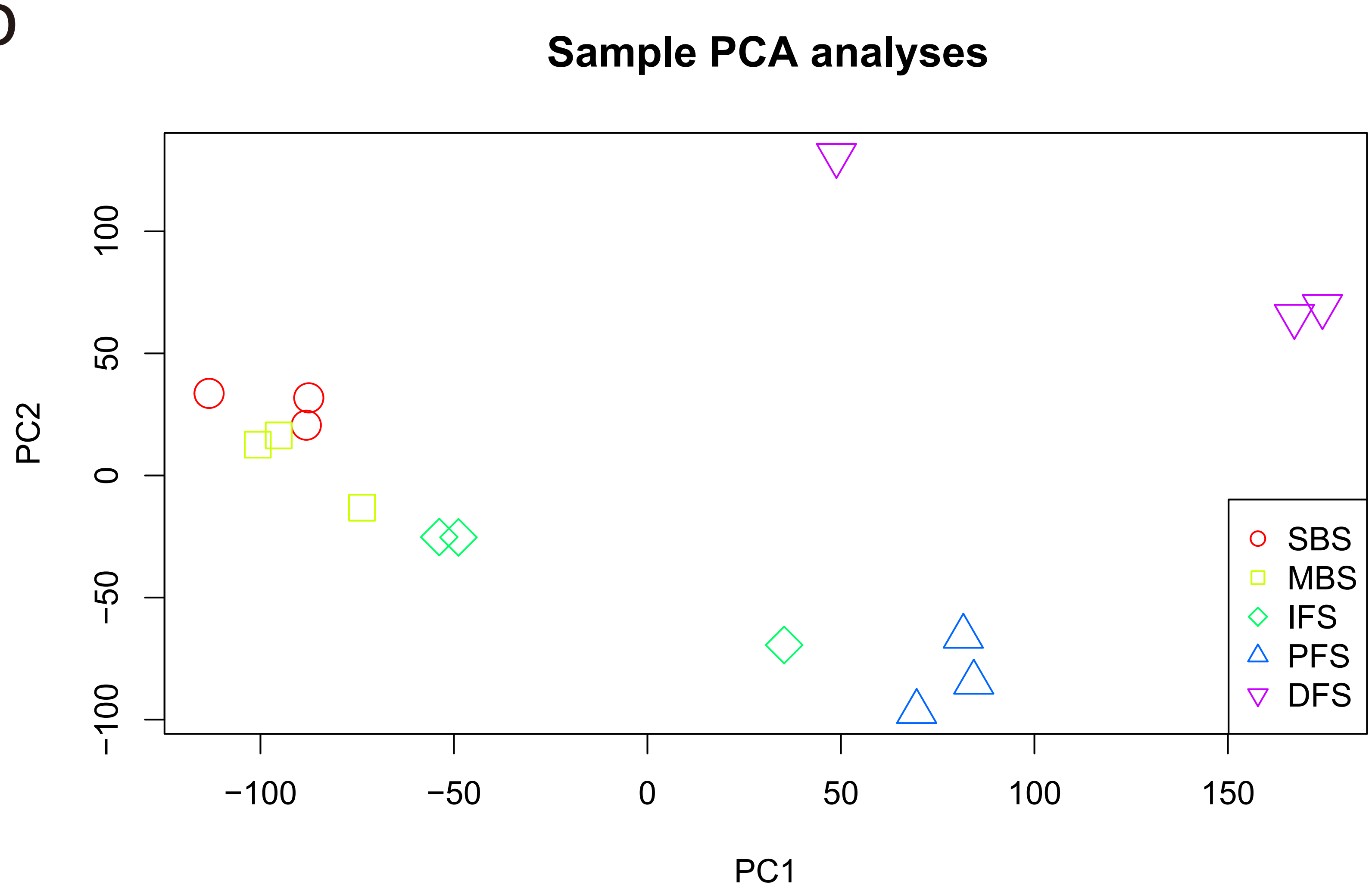

Fig. S21 Filaments **(a)** and sample distances **(b)** of 15 RNA-seq samples at five different stages: small bud stage (SBS), middle bud stage (MBS), initial flowering stage (IFS), peak flowering stage (PFS), and decayed flowering stage (DFS) during flower development, as determined using a principal component analysis.

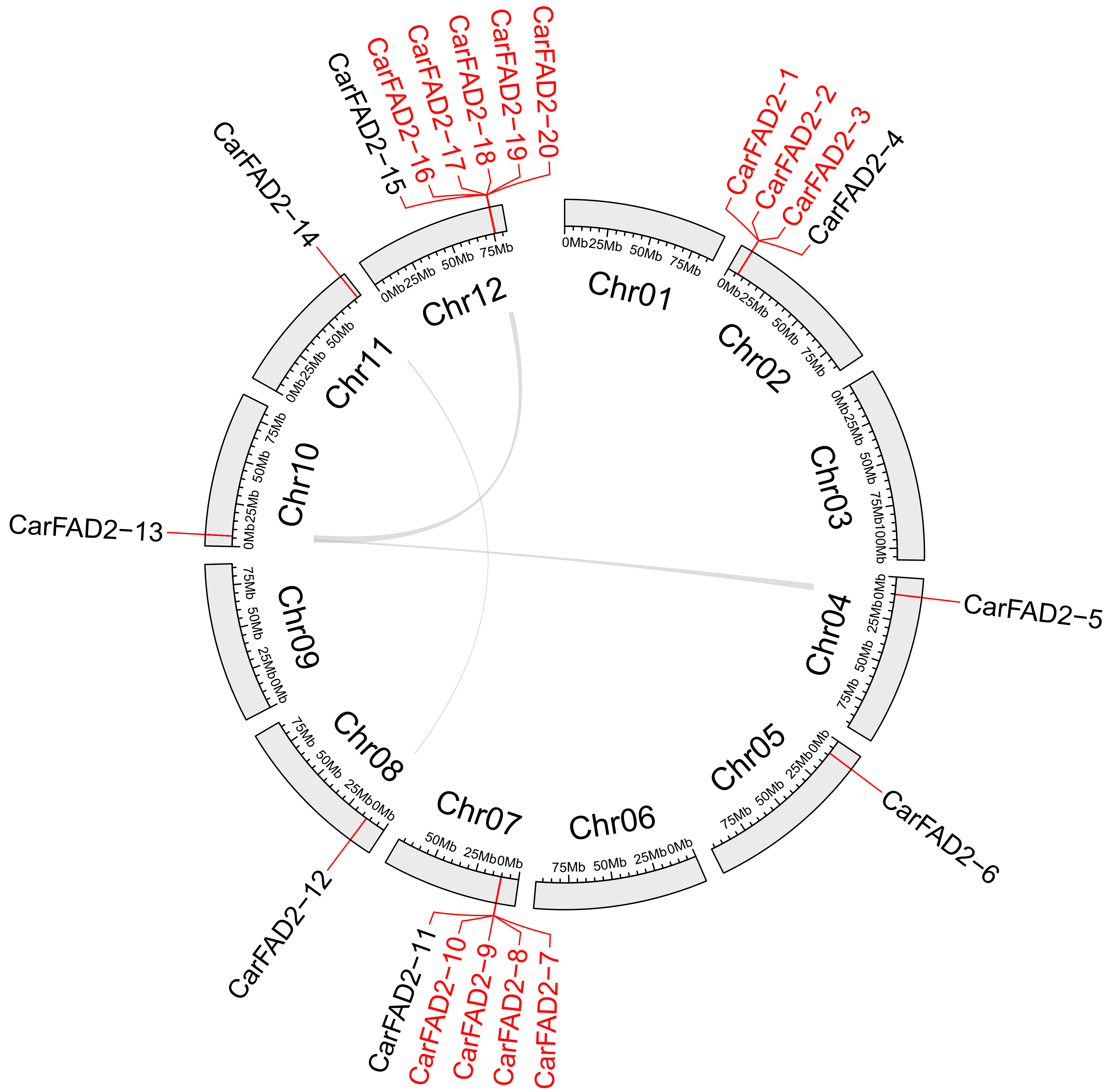

Fig. S22 Distribution of *CarFAD2s* in 12 safflower chromosomes. The links among chromosomes represent collinear blocks, the genes marked with red colors represent the tandem duplication.

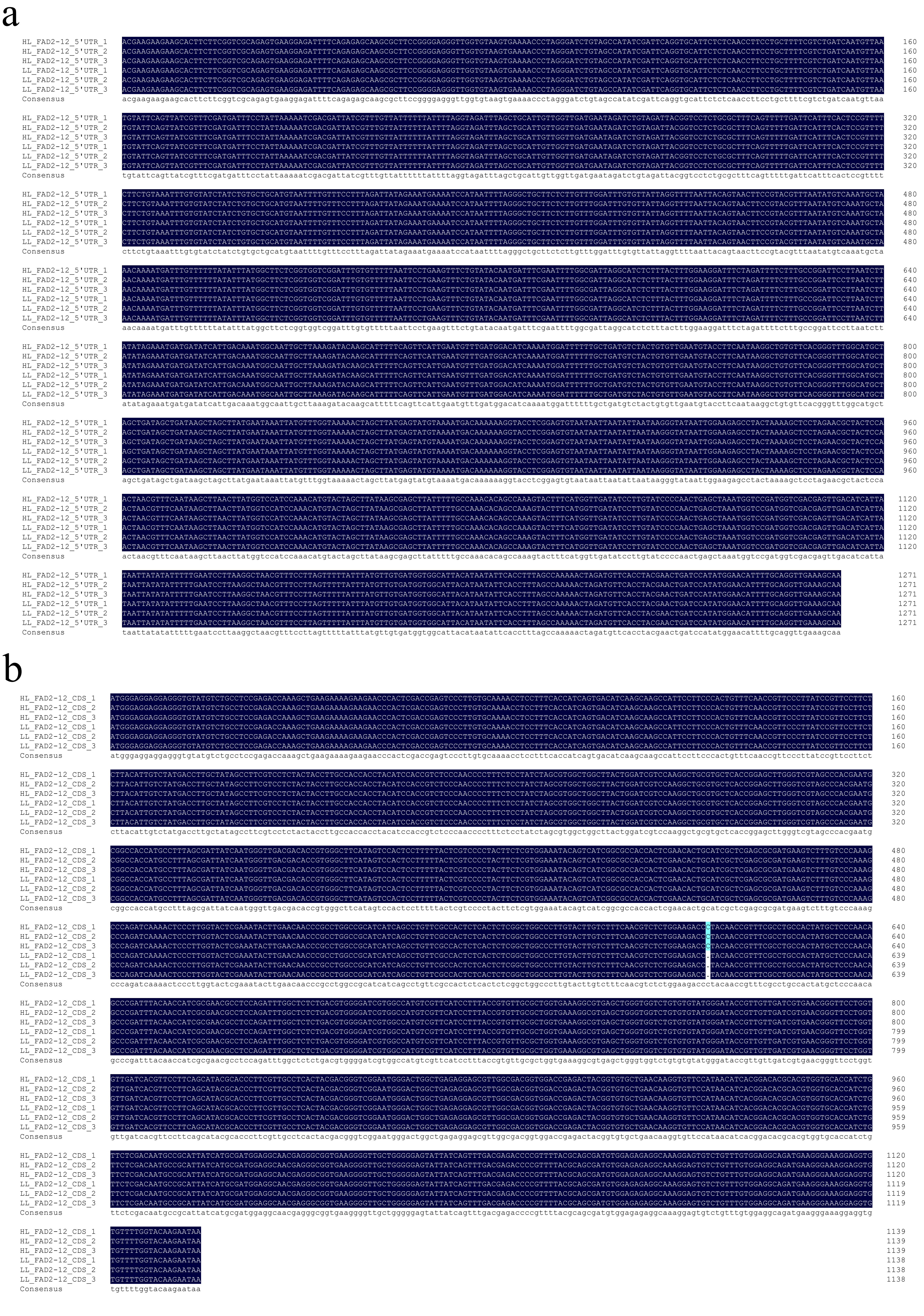

Fig. S23 Sequence alignment of 5' UTR region **(a)** and coding sequence **(b)** of *CarFAD2-12* in 'HL' and 'LL' cultivars.

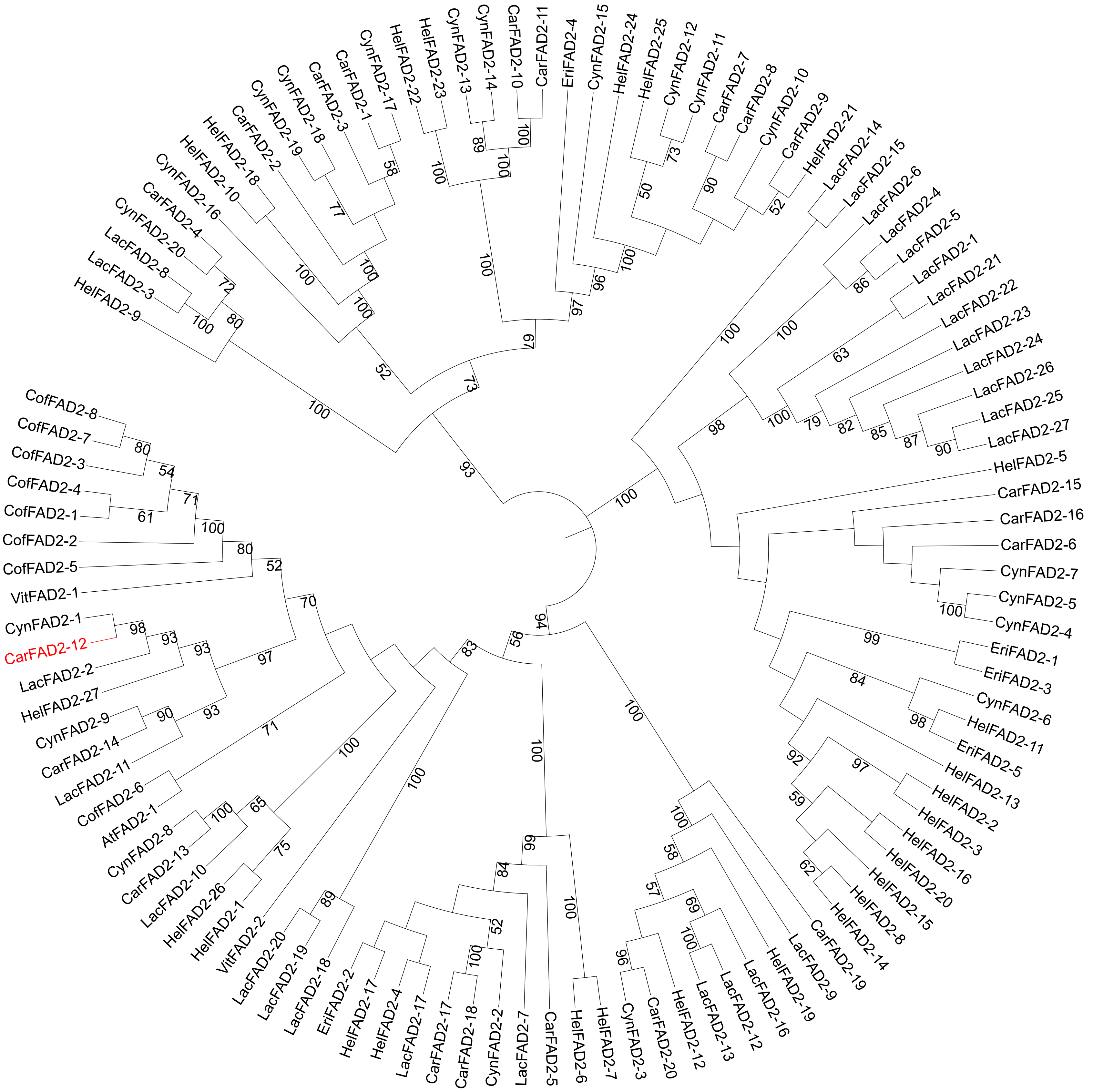

Fig. S24 The phylogenetic tree of *FAD2* (*FATTY ACID DESATURASE 2*) genes of eight species, *Arabidopsis thaliana* (At), *Vitis vinifera* (Vit), *Coffea canephora* (Cof), *Cynara cardunculus* (Cyn), *Erigeron breviscapus* (Eri), *Helianthus annuus* (Hel), *Lactuca sativa* (Lac), and *Carthamus tinctorius* (Car), constructed using Mega X with a Neighbor-Joining method and 500 bootstraps. Only bootstrap values > 50 are shown. The identifier numbers of the *FAD2* members in each species are ordered by their location on the chromosomes. The highly specifically expressed *FAD2* gene in the safflower seeds is *CarFAD2-12*, marked in red.

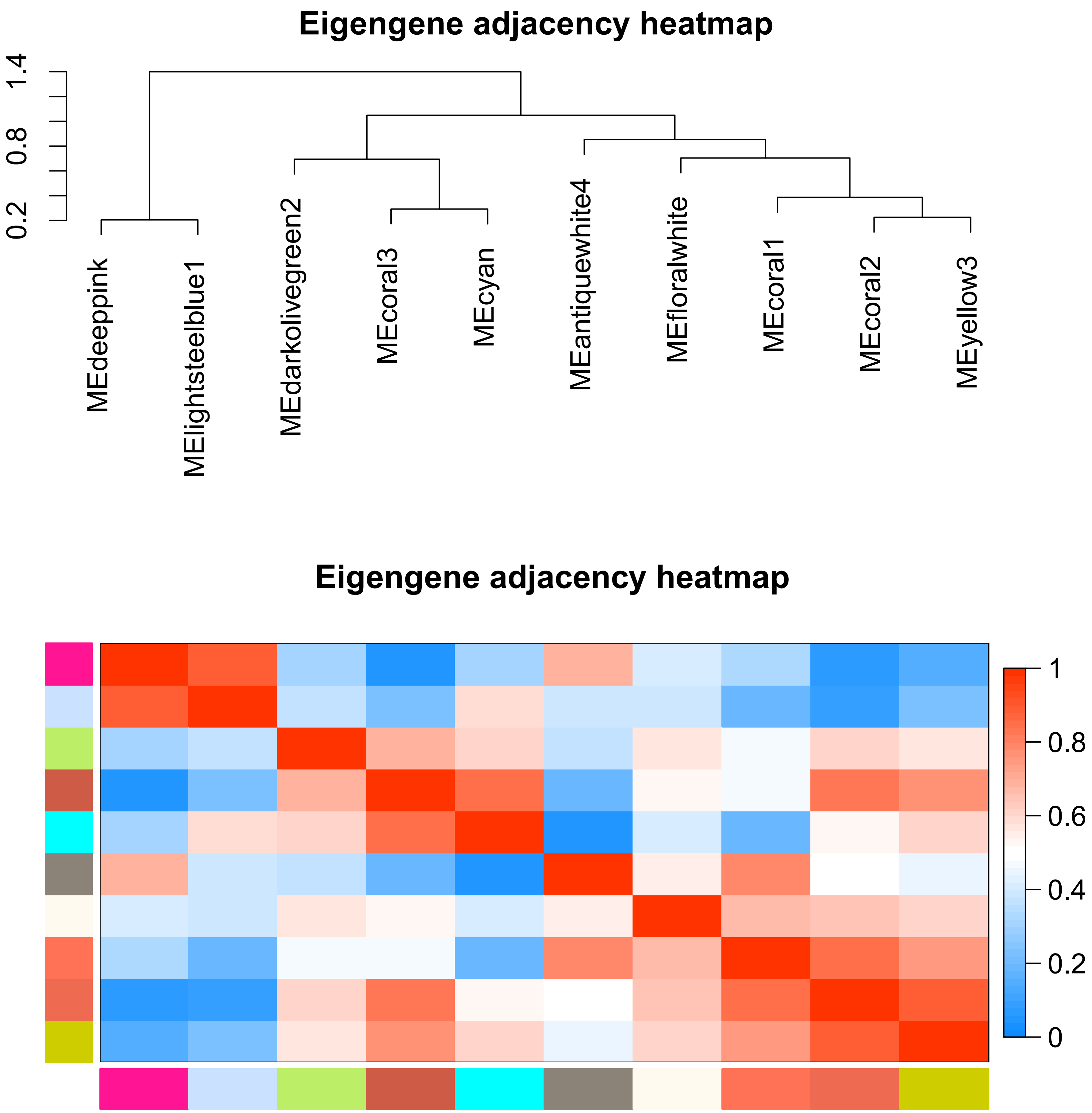

Fig. S25 Relationships of the coexpressed modules from 45 RNA-seq samples in seed development, revealed by the correlation of the module eigengene values (i.e., the first principal component).

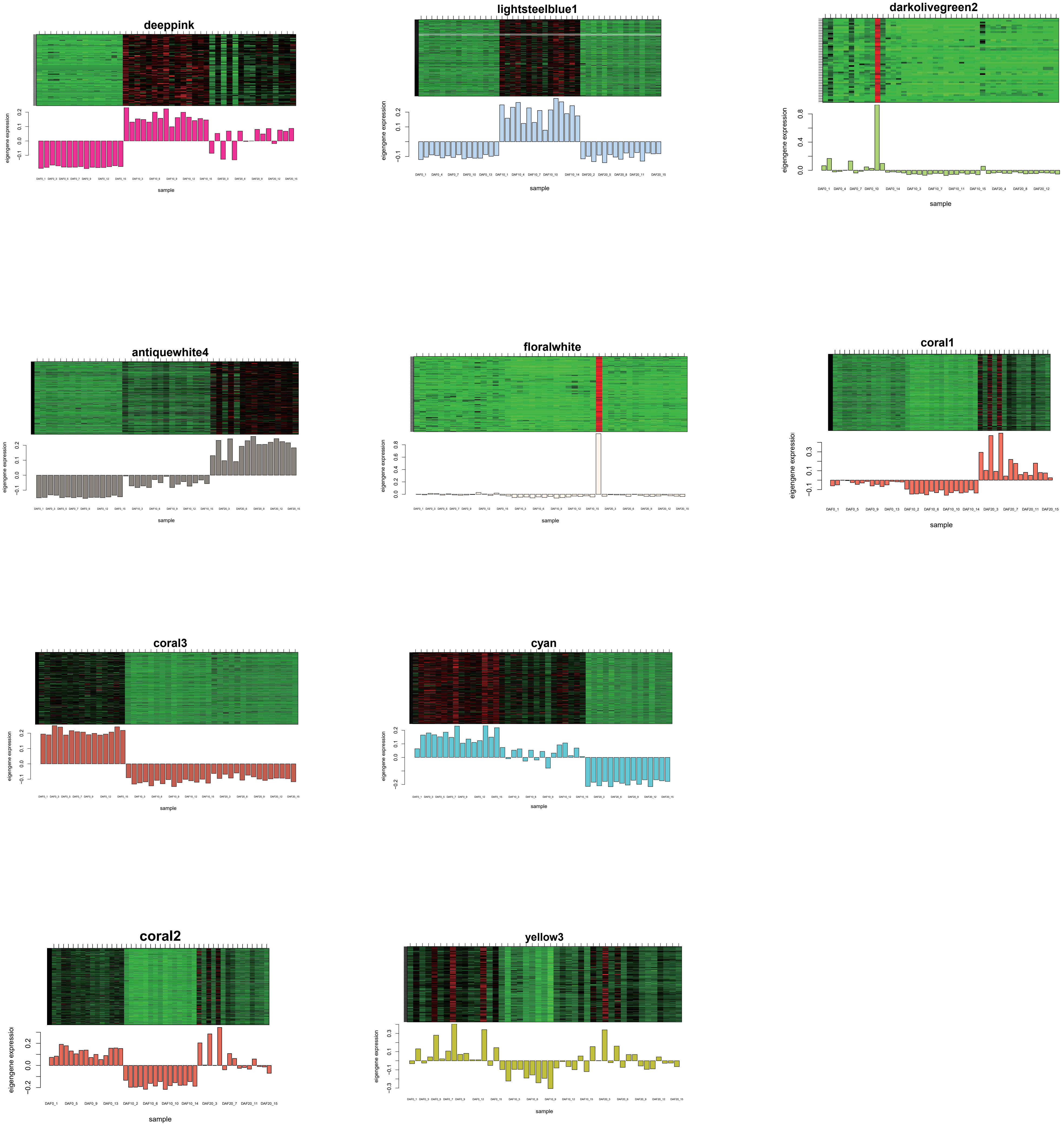

Fig. S26 Expression pattern for each gene module from 45 RNA-seq samples in seed development. The bar plots show eigengene values calculated from the singular value composition.

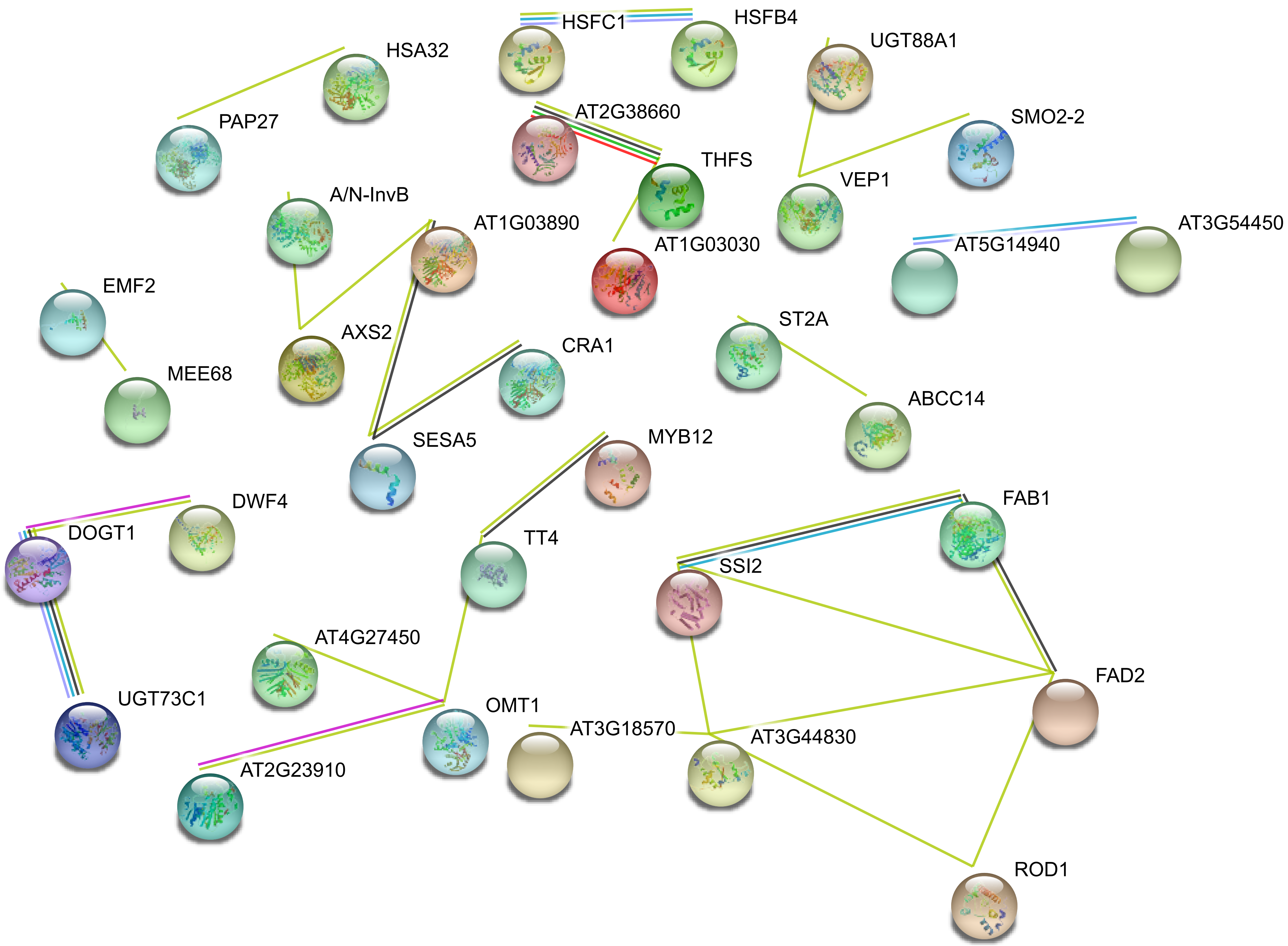

Fig. S27 Associated protein network of *Arabidopsis* homologs of the genes in 'deeppink' module including *CarFAD2-12*, as determined using STRING 11.0.

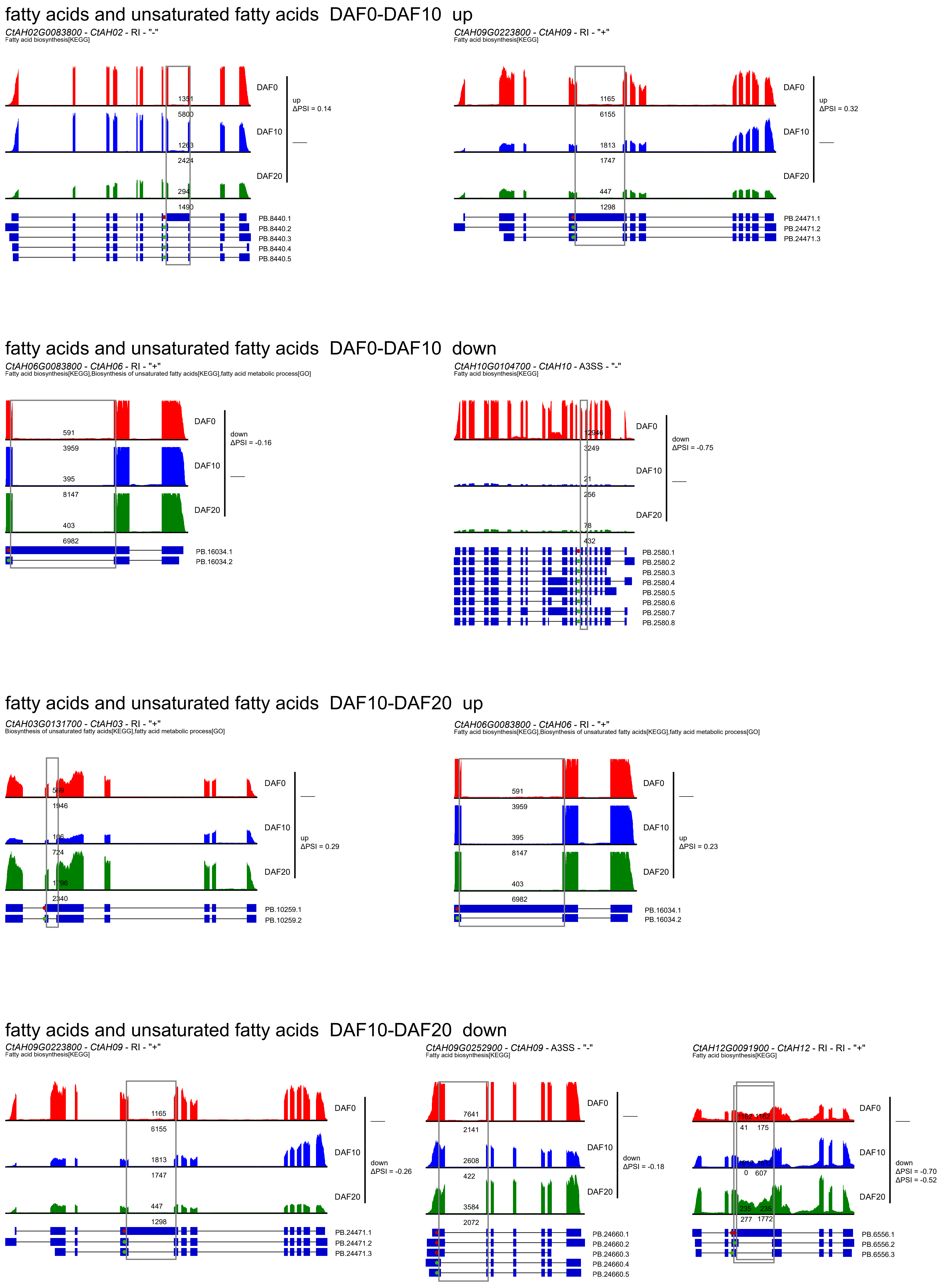

Fig. S28 Differentially alternatively spliced (AS) genes involved in fatty acid biosynthesis. For each gene, AS-covering and total long read counts are shown in AS variants in each stage of seed formation, and differential expression of AS variants is indicated by ΔPSI on the left. The 'up', 'down', and '-' on the left represent upregulated, downregulated, and no differential expression between adjacent groups, respectively. Each vertical line at the bottom represents continuous DASGs among different stages.

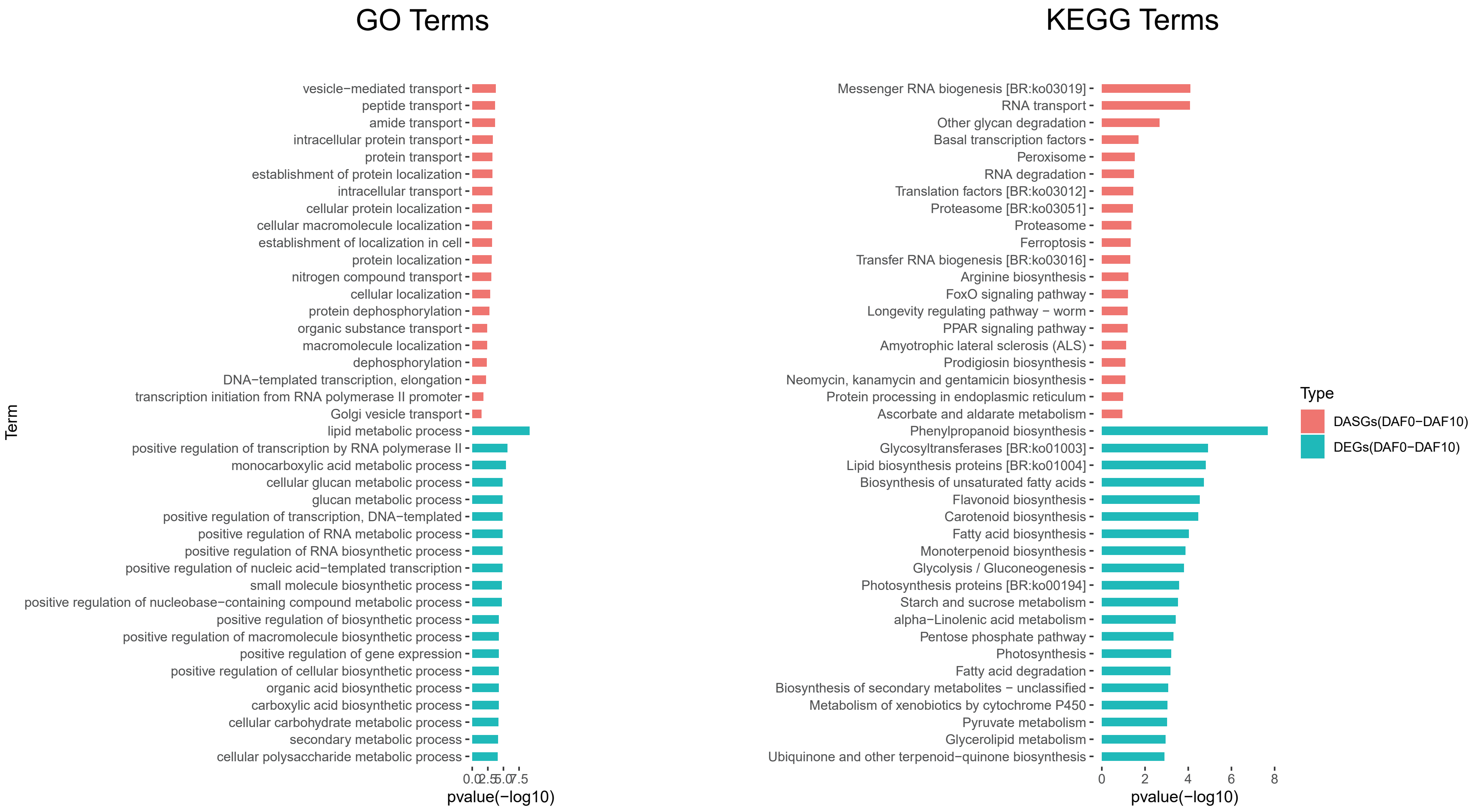

Fig. S29 Top 20 GO and KEGG terms associated with the unique differentially expressed genes and differentially alternatively spliced genes in the comparison of seeds at 10 days after flowering (DAF10) versus DAF0.

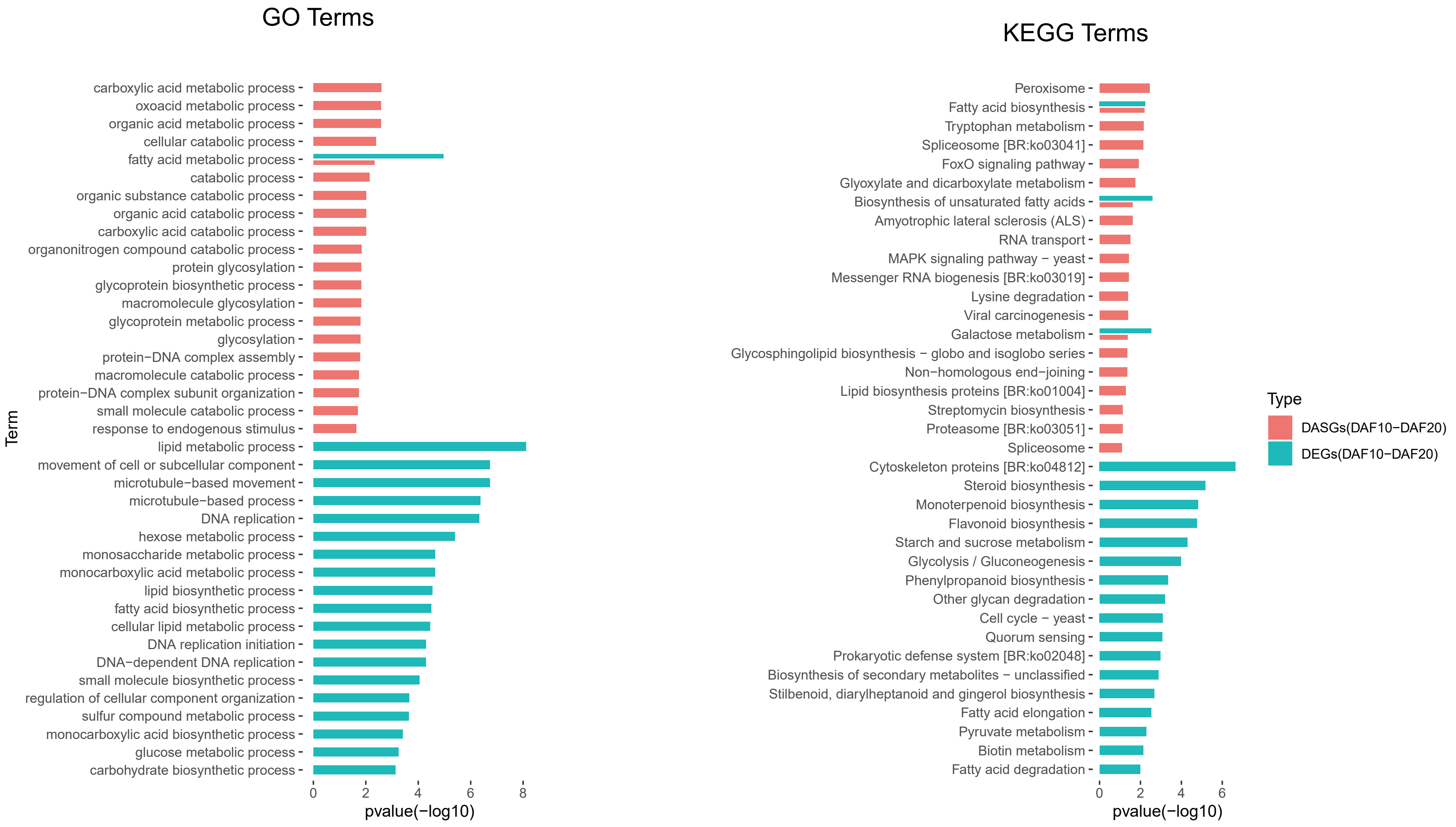

Fig. S30 Top 20 GO and KEGG terms associated with the unique differentially expressed genes and differentially alternatively spliced genes in the comparison of seeds at 20 days after flowering (DAF20) versus DAF10.

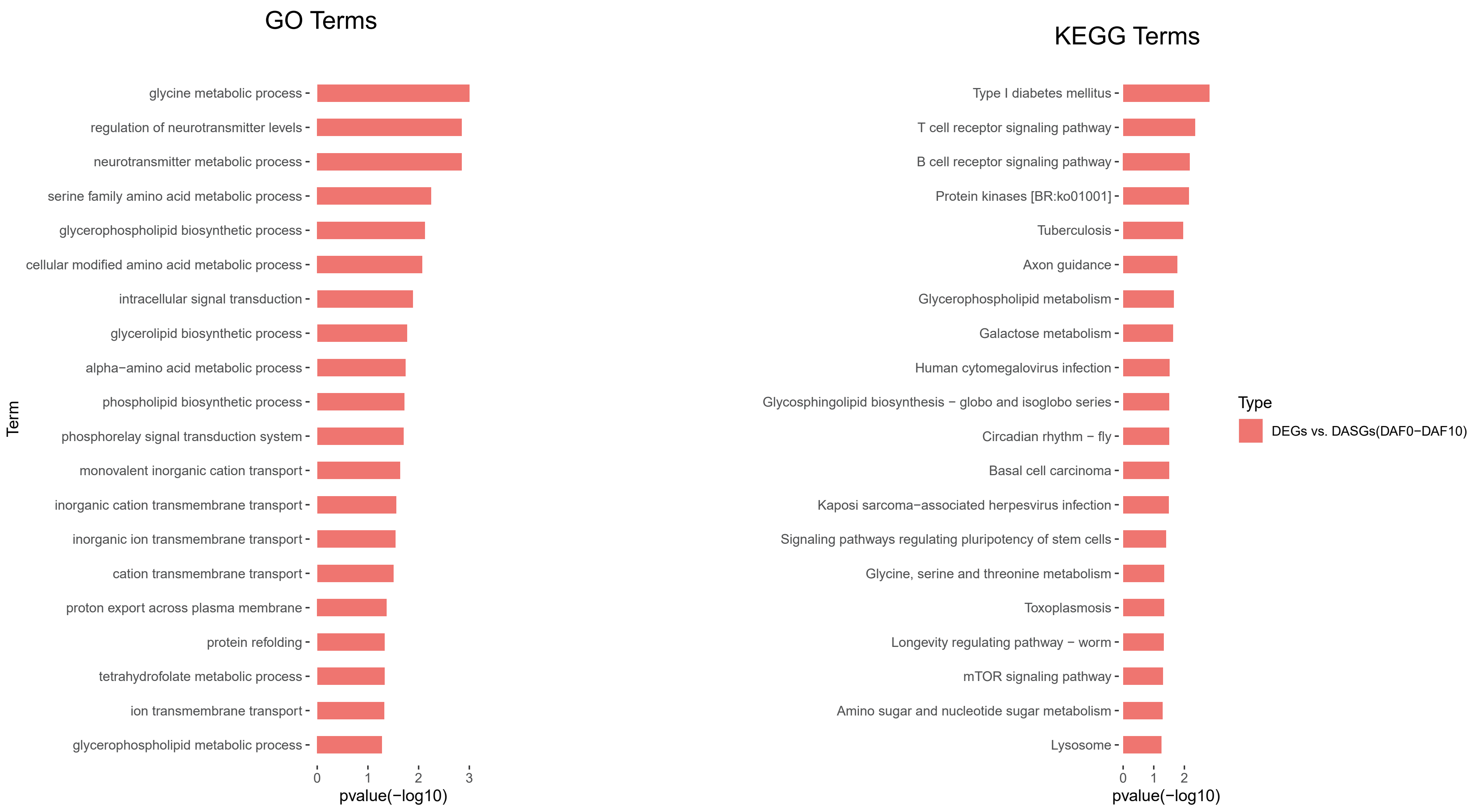

Fig. S31 Top 20 GO and KEGG terms associated with the common differentially expressed genes and differentially alternatively spliced genes in the comparison of seeds at 10 days after flowering (DAF10) versus DAF0.

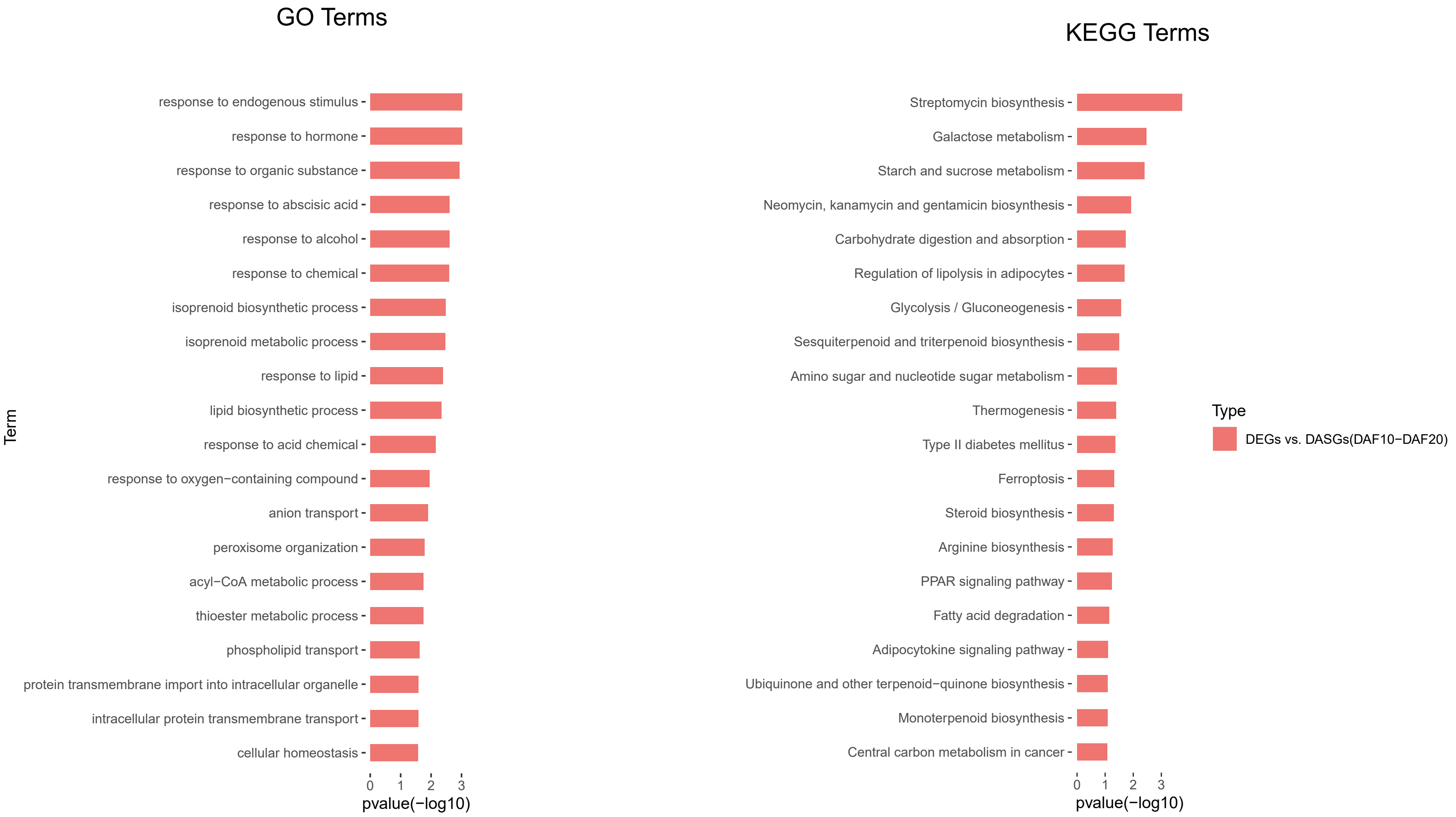

Fig. S32 Top 20 GO and KEGG terms associated with the common differentially expressed genes and differentially alternatively spliced genes in the comparison of seeds at 10 days after flowering (DAF20) versus DAF10.

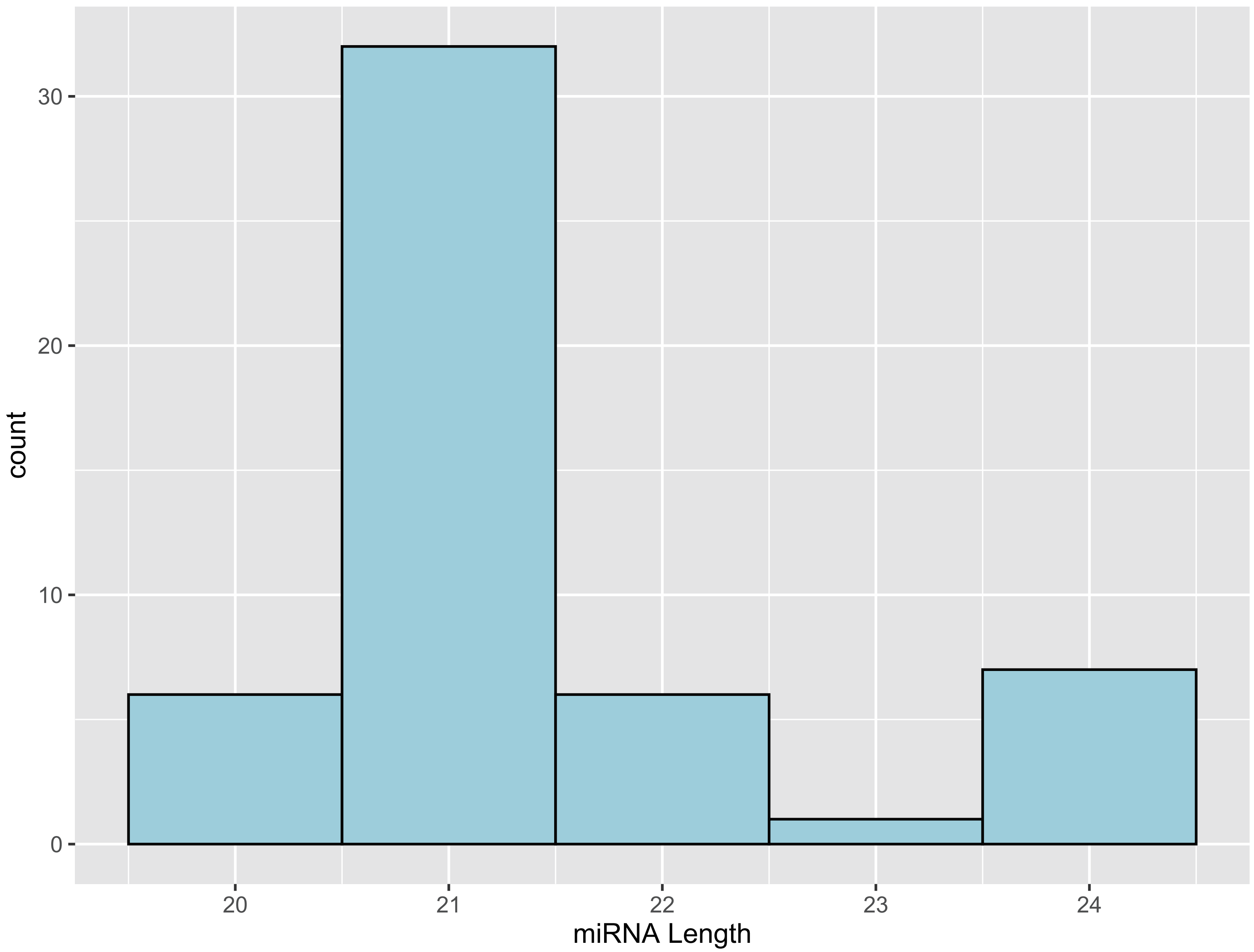

Fig. S33 The length distribution of the 52 identified miRNAs in seed formation.

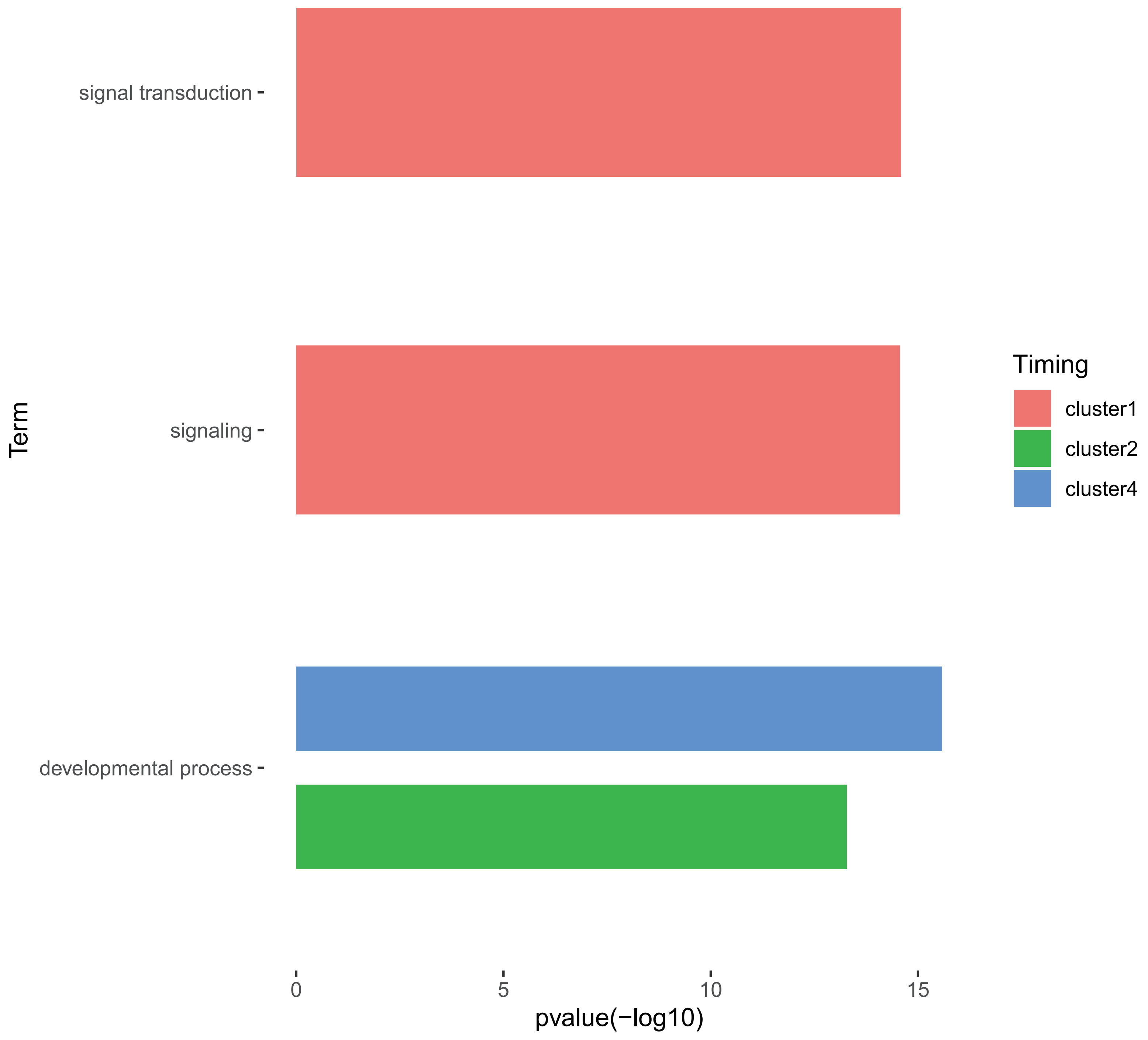

Fig. S34 Enrichment of GO terms in the miRNA-targeted genes for each cluster with a q-value < 0.05.

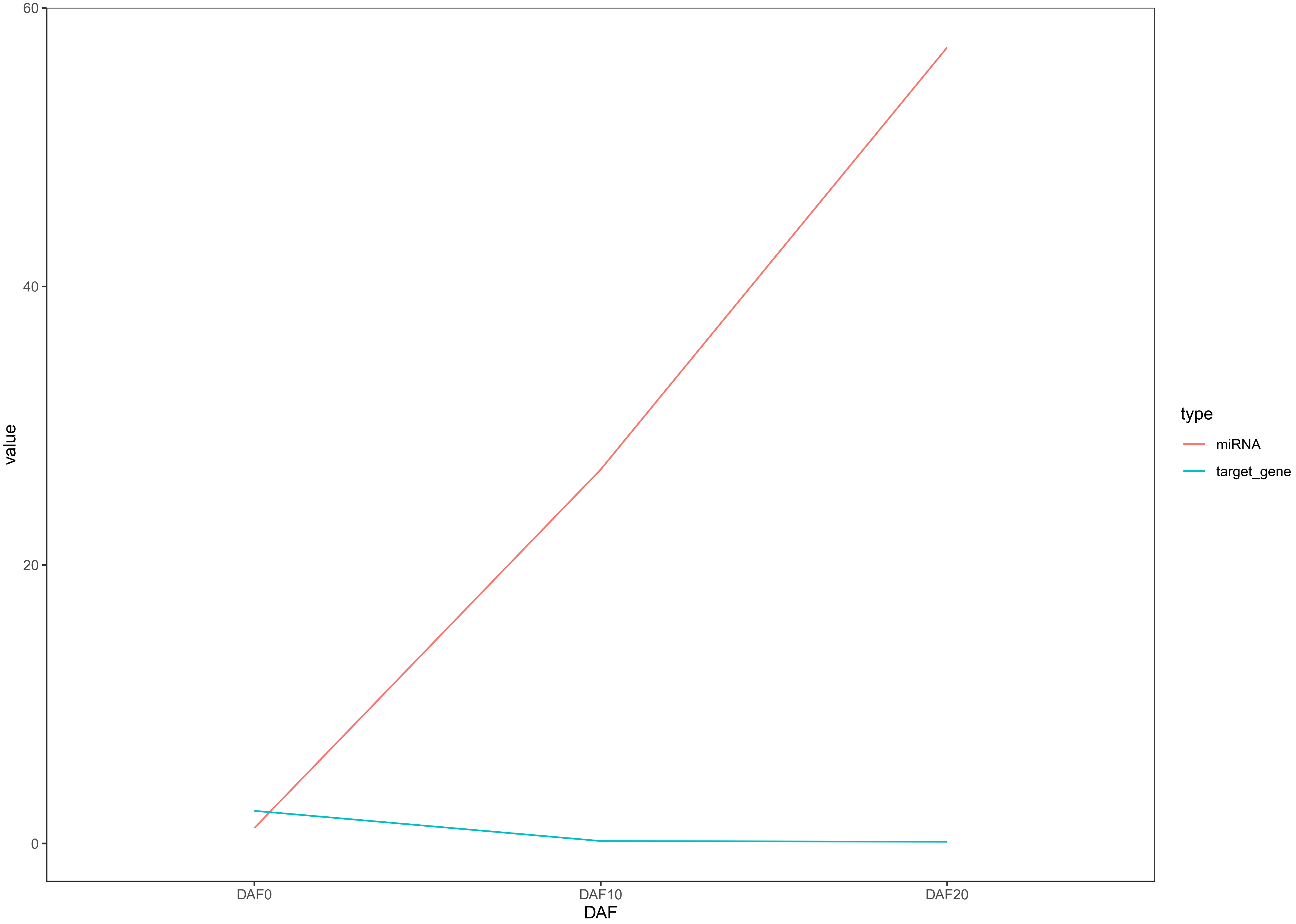

Fig. S35 Expression relationship of Cluster_135896 (ath-miR156h) and its possible target gene *CarFAD2-4* throughout seed development.

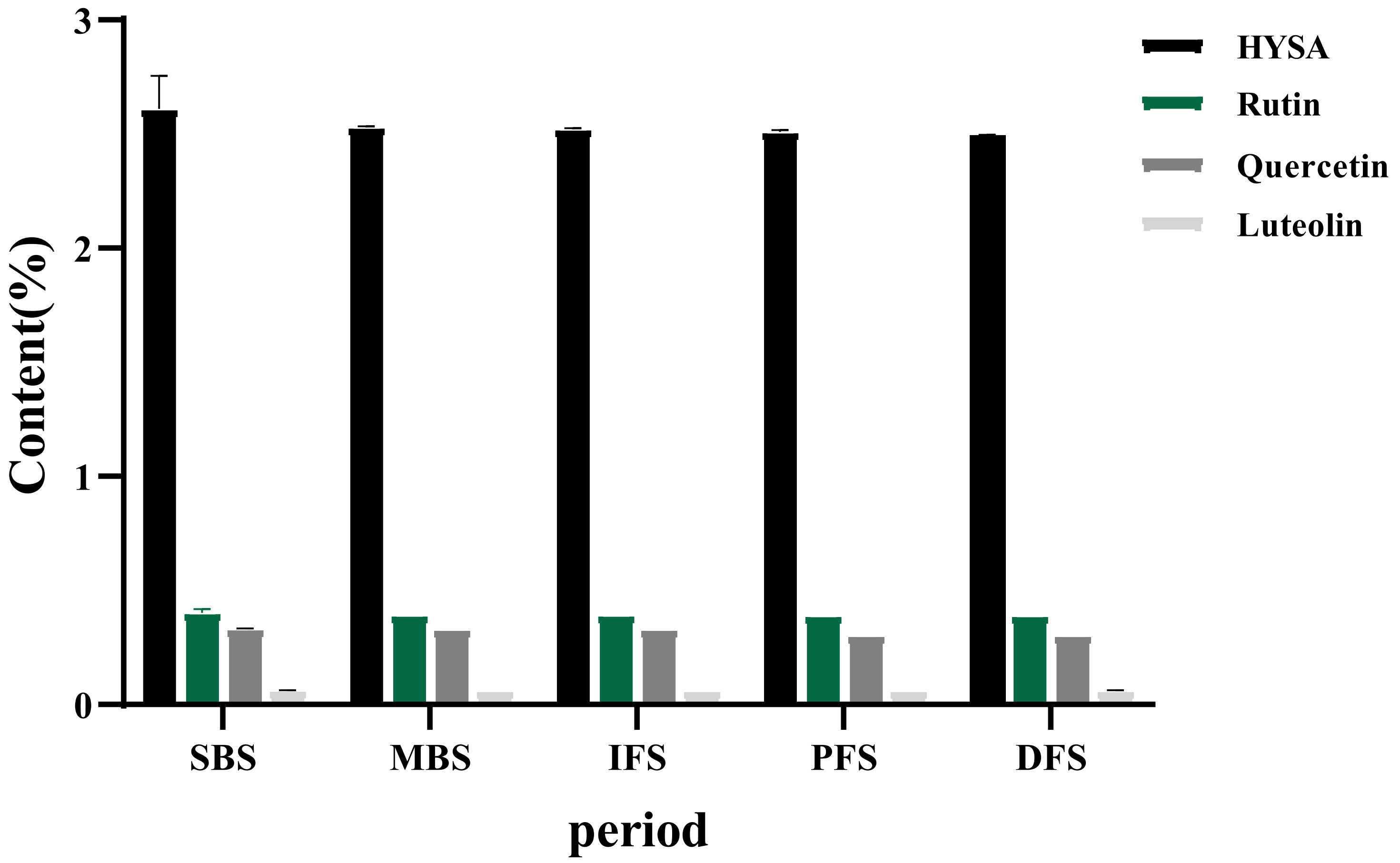

Fig. S36 Contents of HSYA, rutin, luteolin, and quercetin from five different stages: small bud stage (SBS), middle bud stage (MBS), initial flowering stage (IFS), peak flowering stage (PFS), and decayed flowering stage (DFS) during flower development.

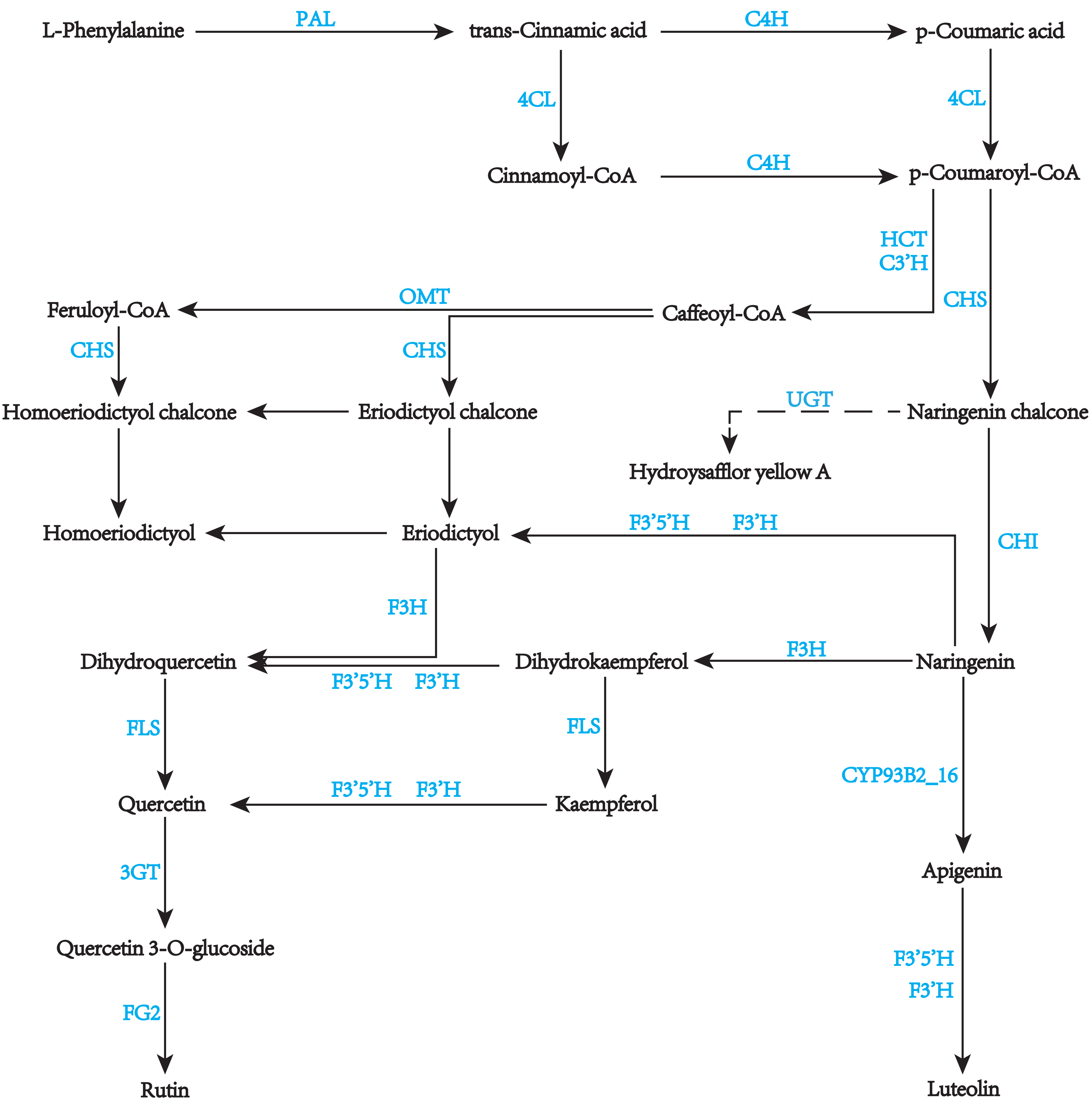

Fig. S37 Schematic diagram of the flavonoid biosynthesis pathway in safflower. Intermediate compounds and enzymes in the flavonoid biosynthesis pathway are represented in black and blue type, respectively.

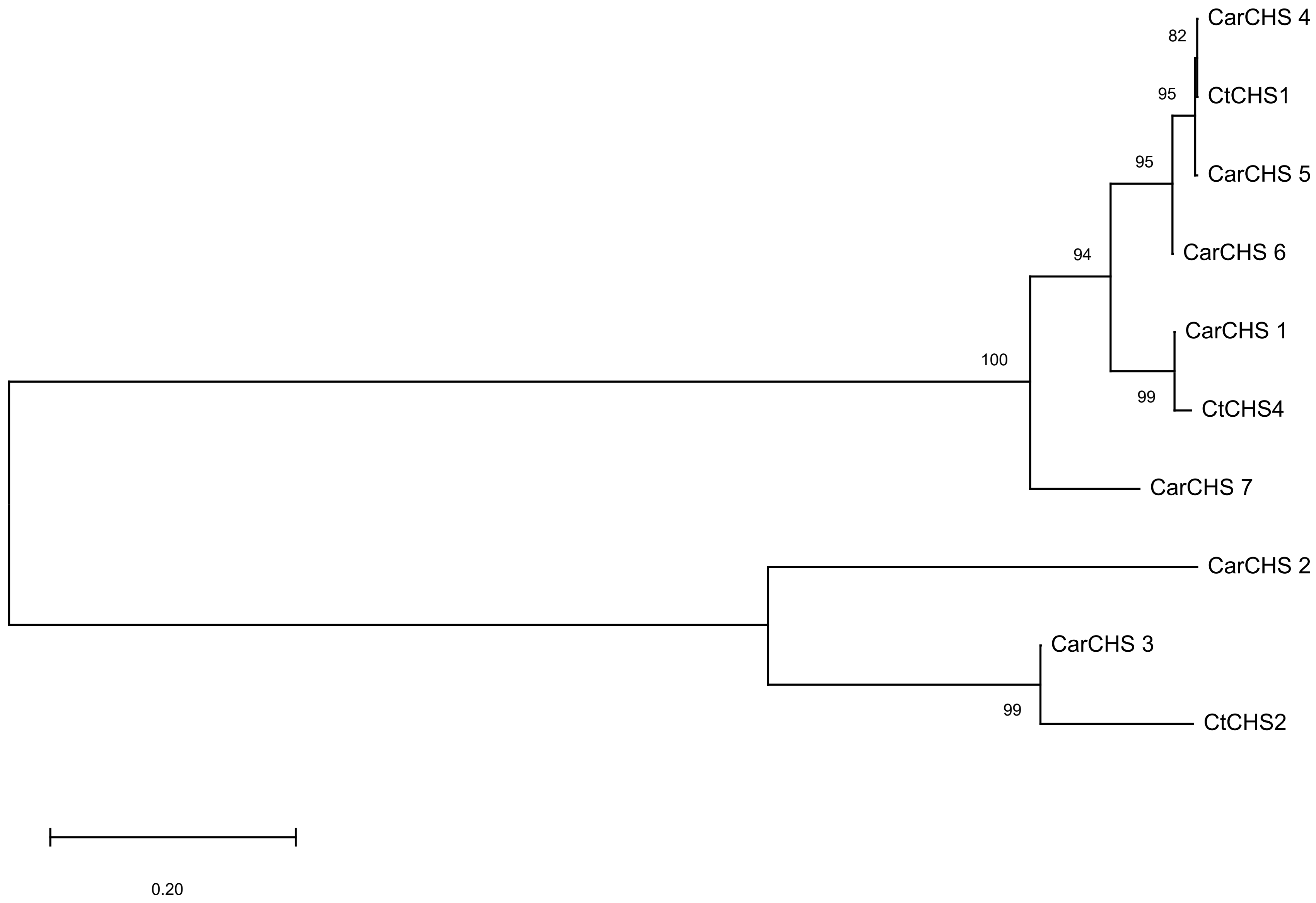

Fig. S38 Gene tree of *CarCHSs* and reported *CtCHS1*, *CtCHS2*, and *CtCHS4* constructed by neighbor-joining method. Only bootstrap values > 50 are shown.

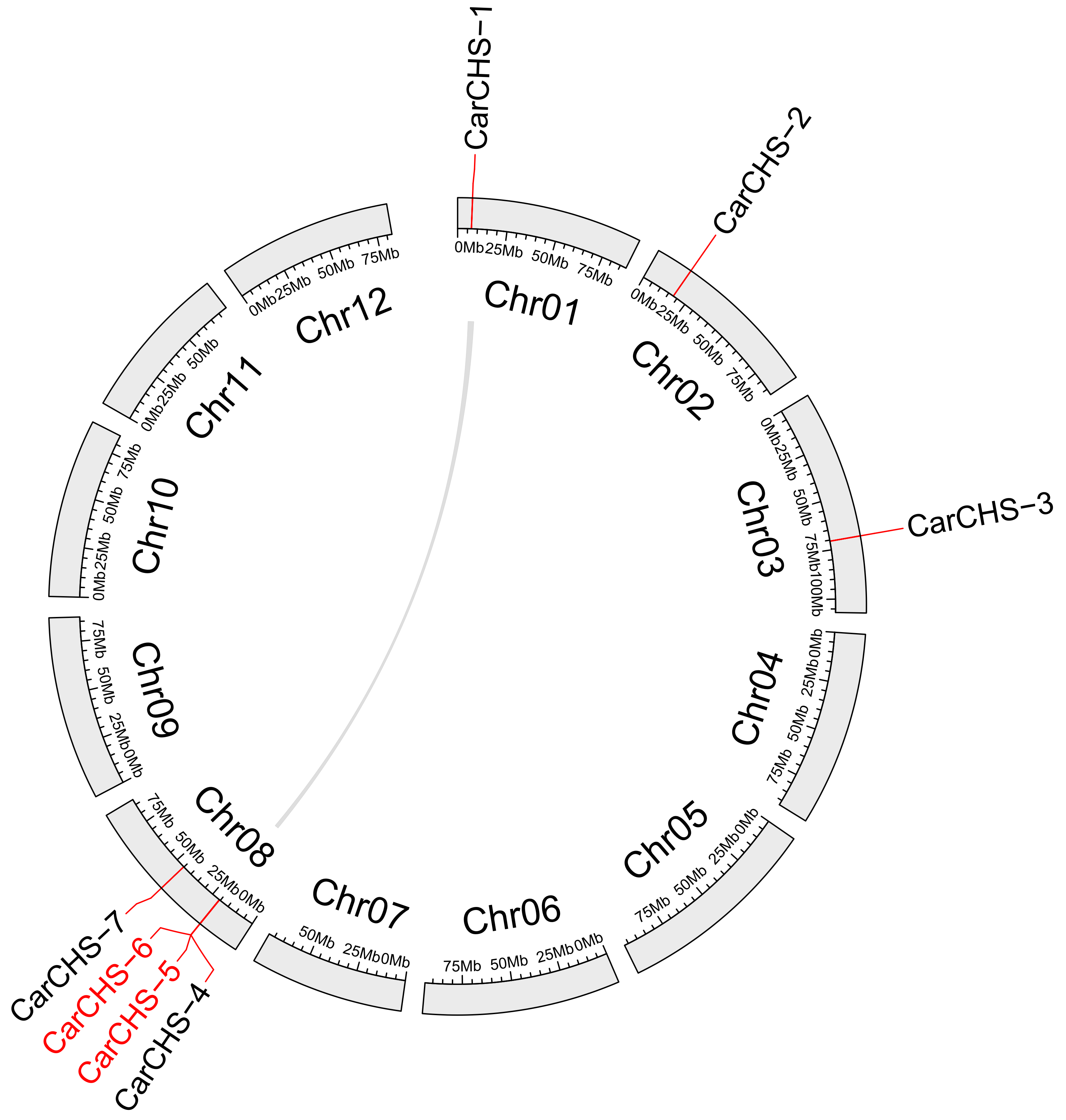

Fig. S39 Distribution of *CarCHSs* in 12 safflower chromosomes. The links among chromosomes represent collinear blocks, the genes marked with red represent the tandem duplication.

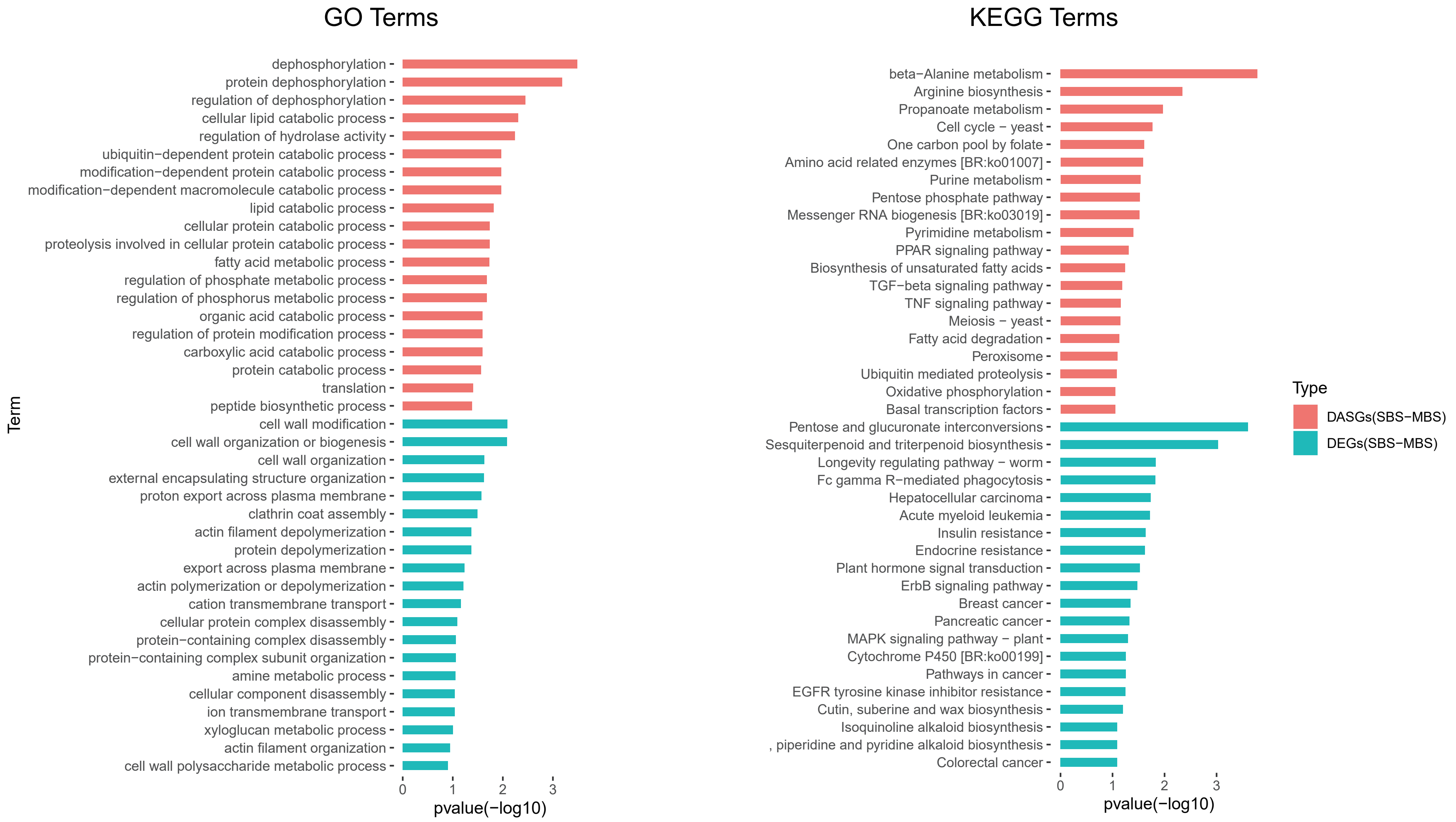

Fig. S40 Top 20 GO and KEGG terms associated with the unique differentially expressed genes and differentially alternatively spliced genes in the comparison of flowers at the small bud stage (SBS) versus middle bud stage (MBS).

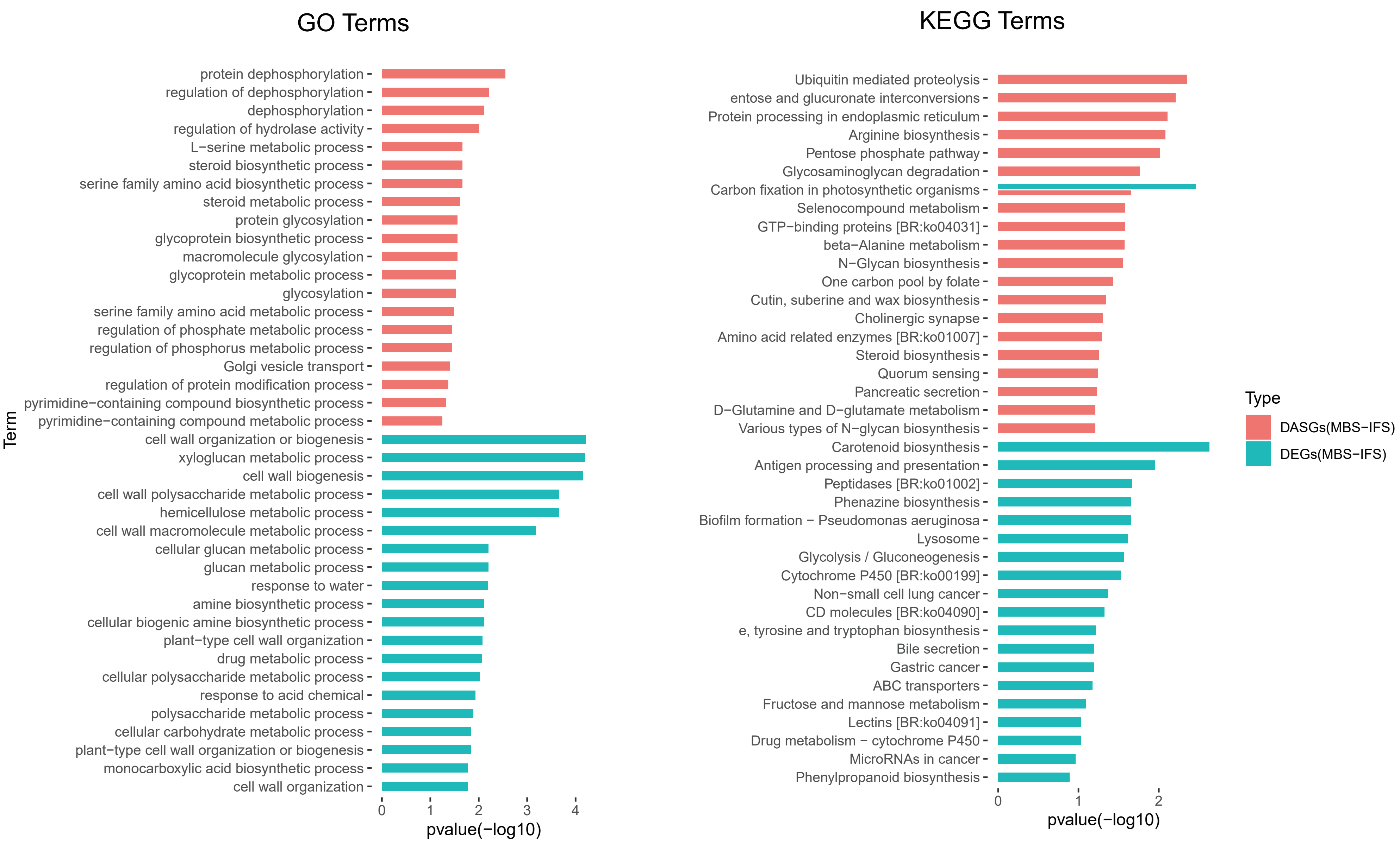

Fig. S41 Top 20 GO and KEGG terms associated with the unique differentially expressed genes and differentially alternatively spliced genes in the comparison of flowers at the middle bud stage (MBS) versus the initial flowering stage (IFS).

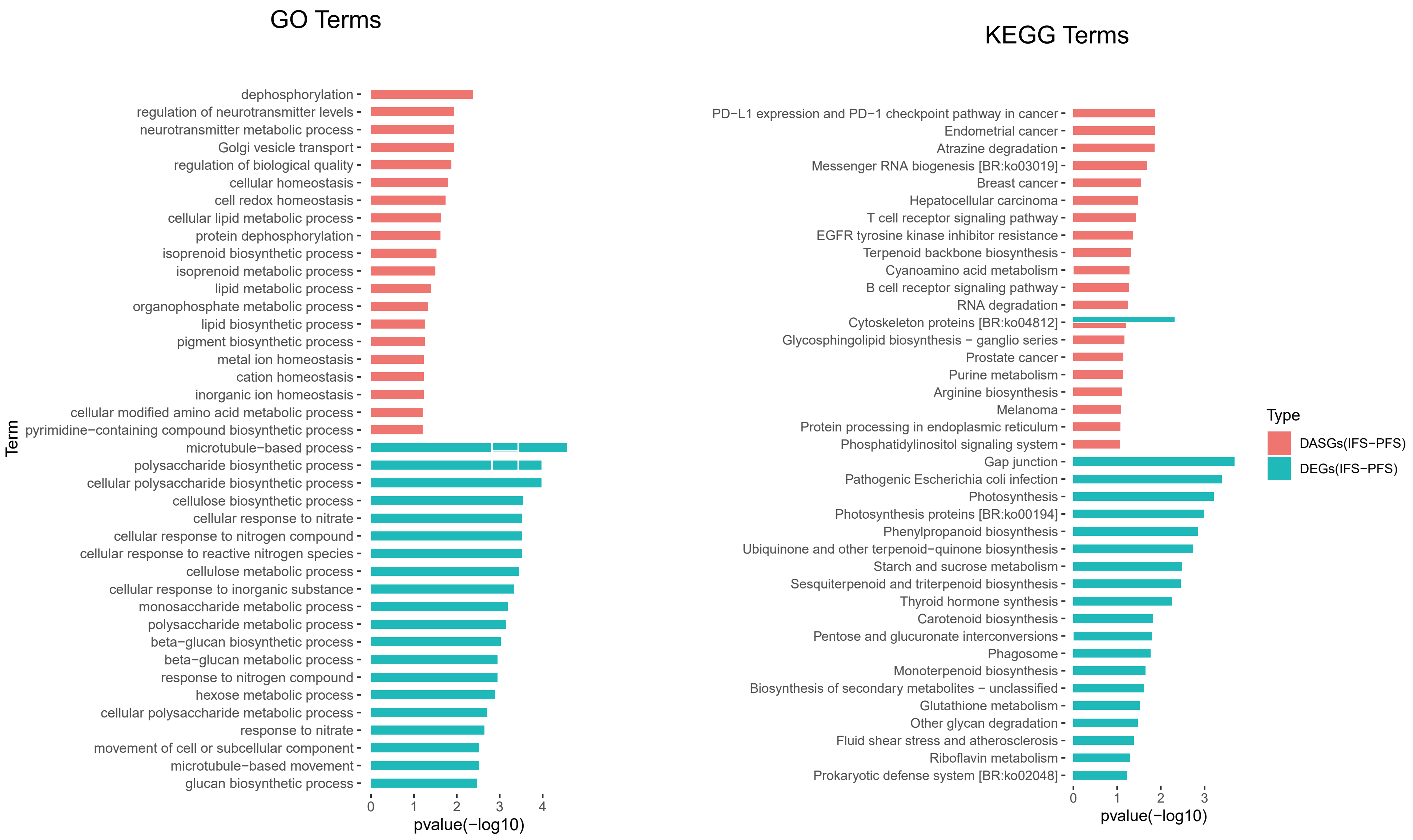

Fig. S42 Top 20 GO and KEGG terms associated with the unique differentially expressed genes and differentially alternatively spliced genes in the comparison of flowers at the initial flowering stage (IFS) versus the peak flowering stage (PFS).

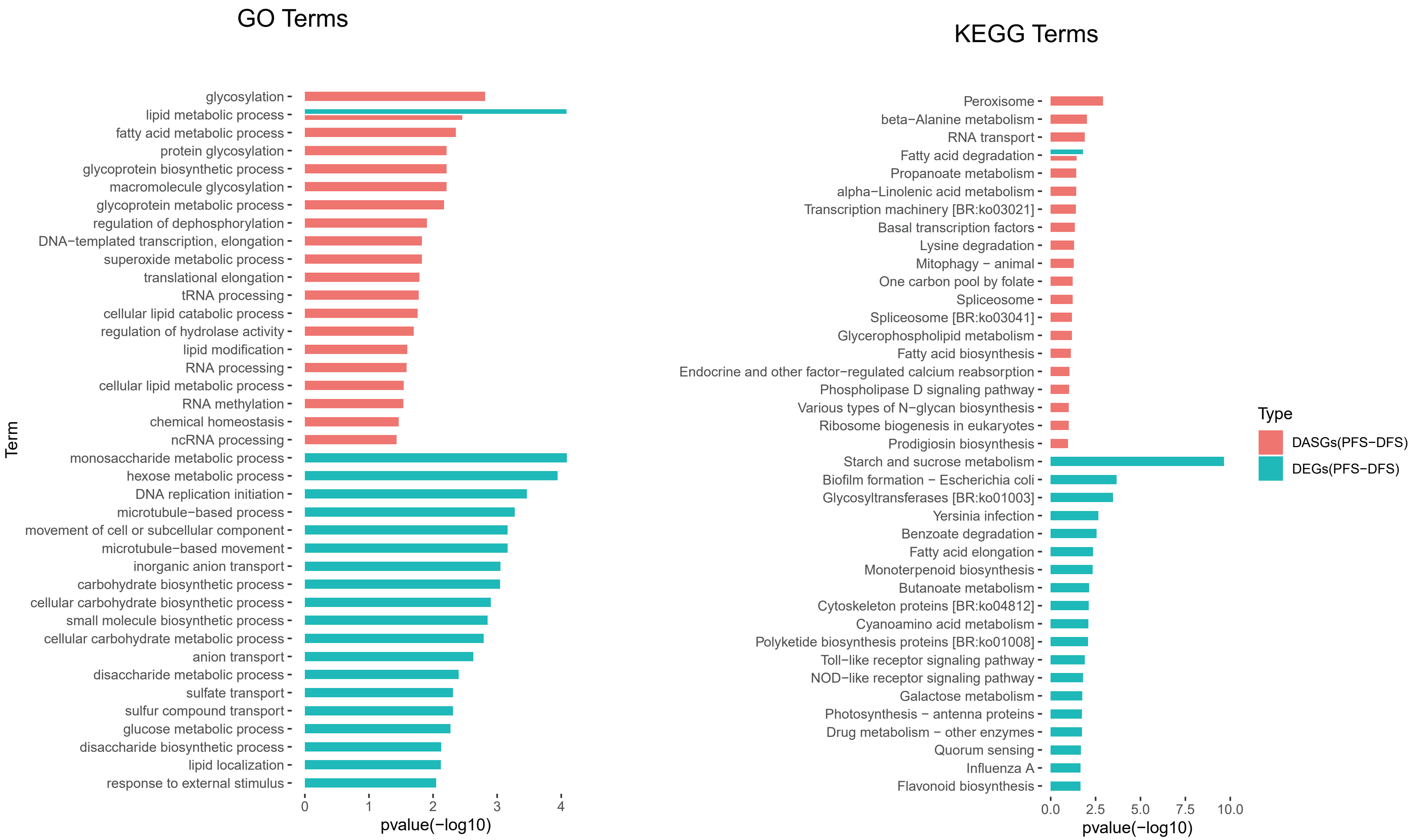

Fig. S43 Top 20 GO and KEGG terms associated with the unique differentially expressed genes and differentially alternatively spliced genes in the comparison of flowers at the peak flowering stage (PFS) versus the decayed flowering stage (DFS).

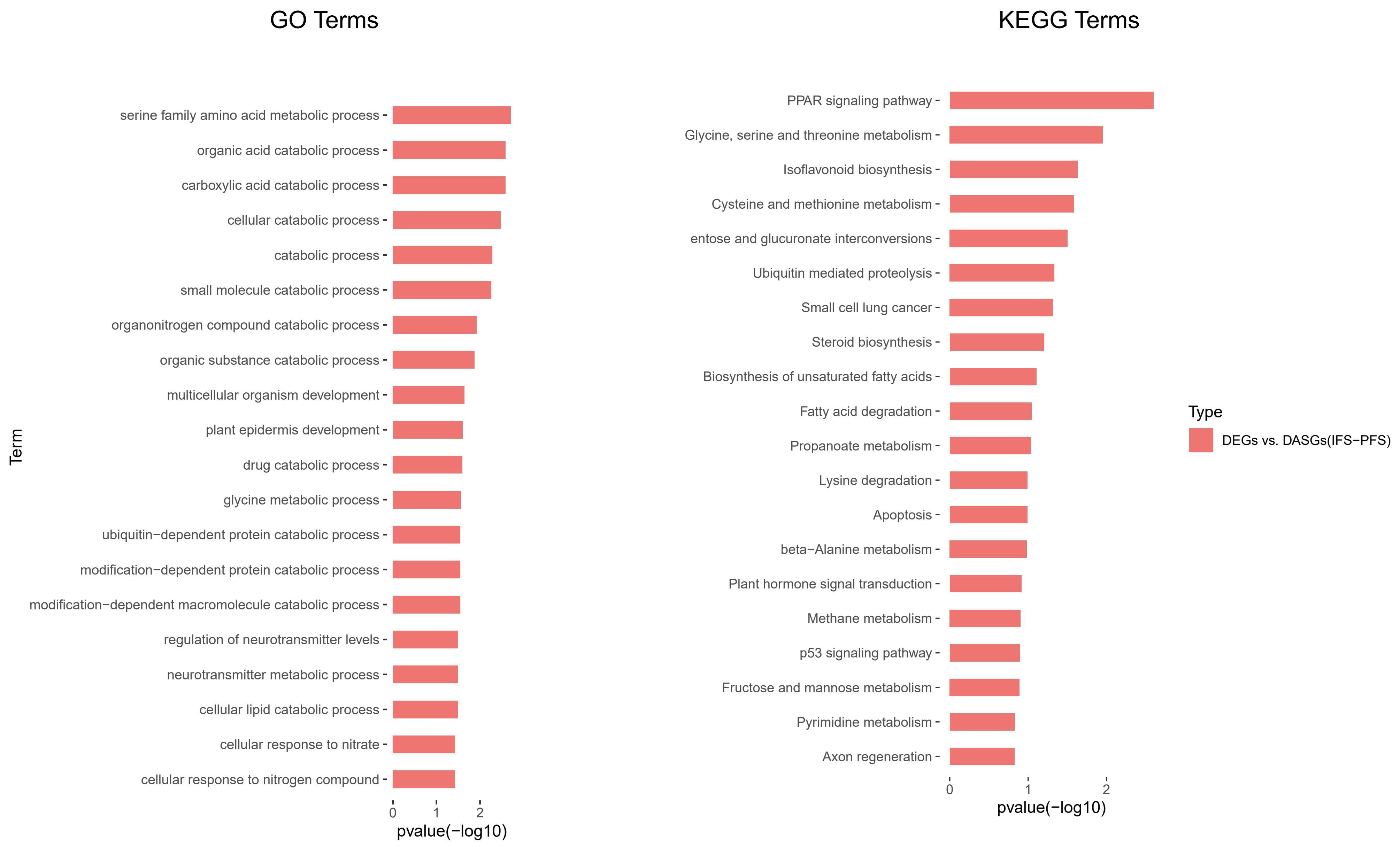

Fig. S44 Top 20 GO and KEGG terms associated with the common differentially expressed genes and differentially alternatively spliced genes in the comparison of flowers at the initial flowering stage (IFS) versus the peak flowering stage (PFS).

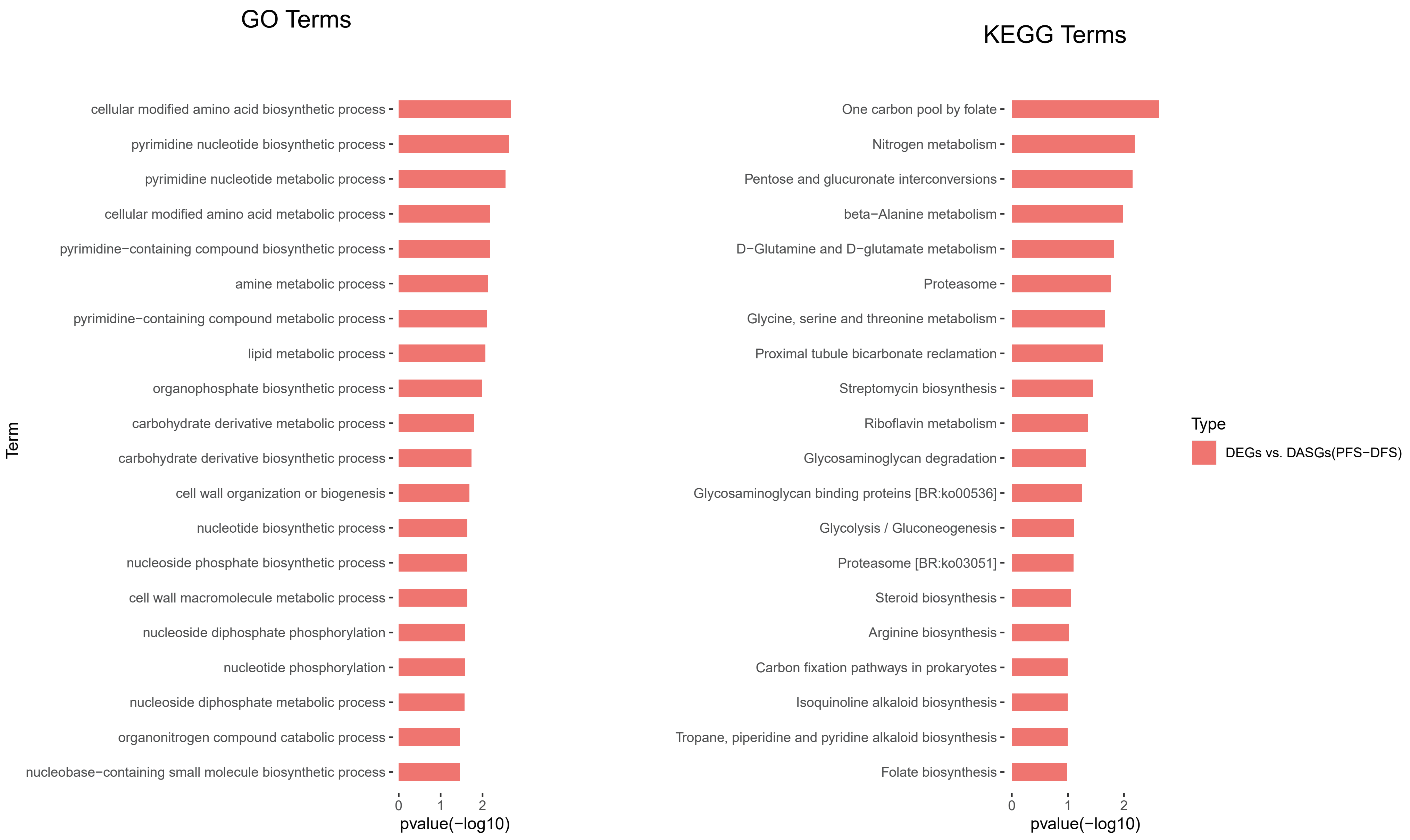

Fig. S45 Top 20 GO and KEGG terms associated with the common differentially expressed genes and differentially alternatively spliced genes in the comparison of flowers at the peak flowering stage versus (PFS) the decayed flowering stage (DFS).

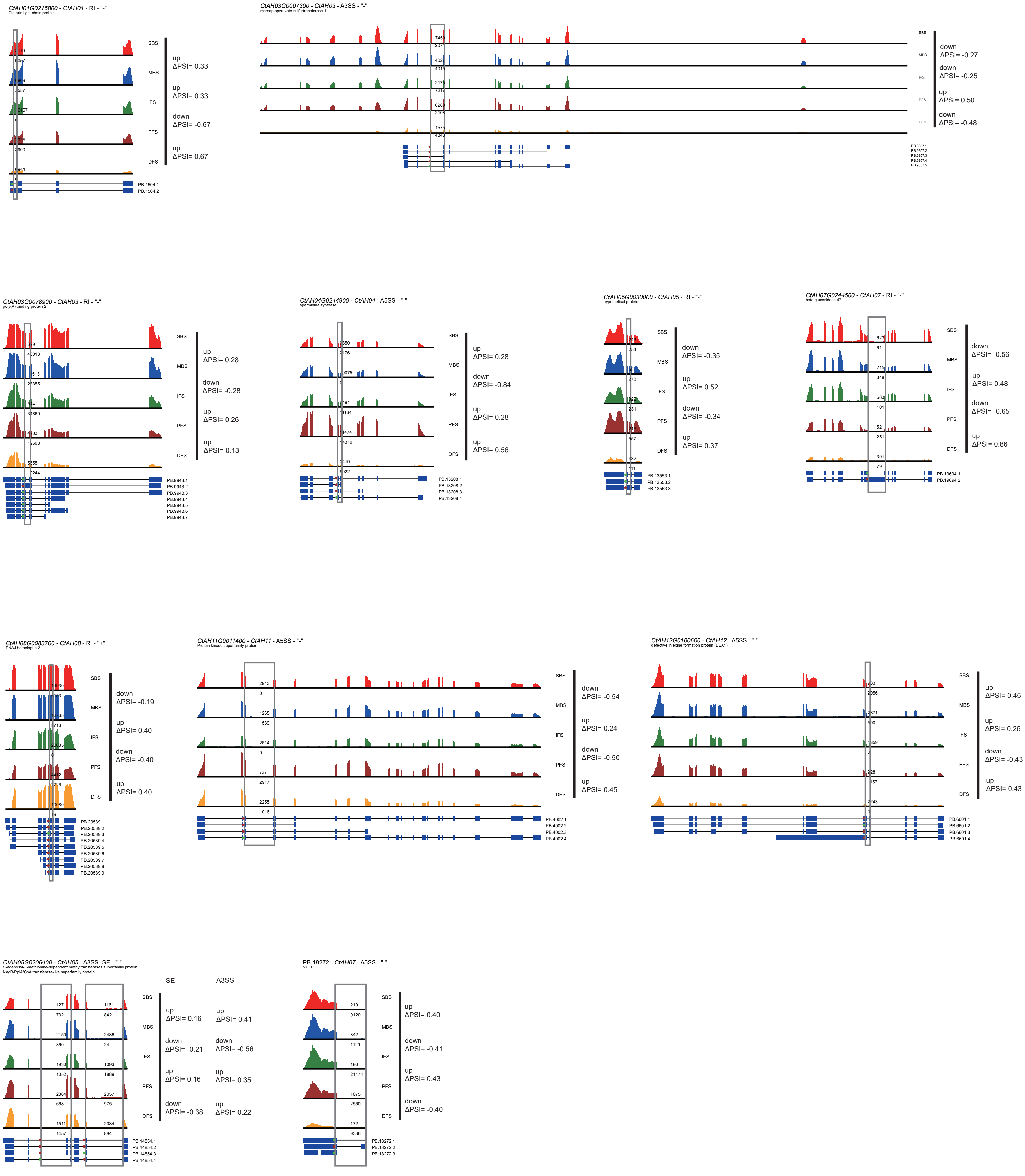

Fig. S46 Expression pattern of the 11 genes common in the differentially alternatively spliced of the four groups (SBS-MBS, MBS-IFS, IFS-PFS, and PFS-DFS). For each gene, AS-covering and total long read counts are shown in AS variants in each stage of flower development, and differential expression of AS variants is indicated by ΔPSI on the left. The 'up', 'down', and '-' on the left represent upregulated, downregulated, and no differential expression between adjacent groups, respectively. Each vertical line at the bottom represents continuous DASGs among different stages.

```
CarCHS4      MASLTDIAEIRKAQRAEGPATILAIGTATPNNCIYQADYPDYYFRITNSEHMVELKQKFKRMCDKSMIRKRYMHITEEFL    80
PB.21282.1   MASLTDIAEIRKAQRAEGPATILAIGTATPNNCIYQADYPDYYFRITNSEHMVELKQKFKRMCDKSMIRKRYMHITEEFL    80
PB.21282.2   MASLTDIAEIRKAQRAEGPATILAIGTATPNNCIYQADYPDYYFRITNSEHMVELKQKFKRMCDKSMIRKRYMHITEEFL    80
Consensus    masltdiaeirkaqraegpatilaigtatpnnciyqadypdyyfritnsehmvelkqkfkrmcdksmirkrymhiteefl

CarCHS4      KENPNMCEYMAPSLDARQDVVVVEVPKLGKEAATKAIKEWGQPKSKITHLIVCTTSGVDMPGADYQITKLLGLRPSVKRF   160
PB.21282.1   KENPNMCEYMAPSLDARQDVVVVEVPKLGKEAATKAIKEWGQPKSKITHLIVCTTSGVDMPGADYQITKLLGLRPSVKRF   160
PB.21282.2   KENPNMCASTVTCRVRVCCSLLTRGRSRRRMVAPPPEKVWIGVFCLGLVLVRWRRWSFTVSQPPPLPLKIHNYYS..NPL   158
Consensus    kenpnmceymapsldarqdvvvvevpklgkeaatkaikewgqpkskithlivcttsgvdmpgadyqitkllglrpsvkrf

CarCHS4      MMYQQGCFAGGTVLRLAKDLAENNKGARVLVVCSEITAVTFRGPNDTHLDSLVGQALFGDGAAAVIVGADPDLATERPLF   240
PB.21282.1   MMYQQGCFAGGTVLRLAKDLAENNKGARVLVVCSEITAVTFRGPNDTHLDSLVGQALFGDGAAAVIVGADPDLATERPLF   240
PB.21282.2   CLYNNAELG..FFLYILINLESVCVFFMLCMILQWVVPIVIWLLNSMSLICLLX..........................   210
Consensus    mmyqqgcfaggtvlrlakdlaennkgarvlvvcseitavtfrgpndthldslvgqalfgdgaaavivgadpdlaterplf

CarCHS4      EMVSAAQTILPDSEGAIDGHLREVGLTFHLLKDVPGLISKNIEKALVQAFSPLGISDWNSLFWIAHPGGPAILDQVEQKL   320
PB.21282.1   EMVSAAQTILPDSEGAIDGHLREVGLTFHLLKDVPGLISKNIEKALVQAFSPLGISDWNSLFWIAHPGGPAILDQVEQKL   320
PB.21282.2   ................................................................................   210
Consensus    emvsaaqtilpdsegaidghlrevgltfhllkdvpglisknieka lvqafsplgisdwnslfwiahpggpaildqveqkl

CarCHS4      GLKEEKMRATRHVLSEYGNMSSACVLFIIDEMRKKSAEDGCATTGEGLDWGVLFGFGPGLTVETVVLHSVPTTPIA       396
PB.21282.1   GLKEEKMRATRHVLSEYGNMSSACVLFIIDEMRKKSAEDGCATTGEGLDWGVLFGFGPGLTVETVVLHSVPTTPIA       396
PB.21282.2   ............................................................................       210
Consensus    glkeekmratrhvlseygnmssacvlfiidemrkksaedgcattgegldwgvlfgfgpgltvetvvlhsvpttpia
```

Fig. S47 Protein sequence alignment of two alternative-splicing variants of *CarCHS4* in flower development.

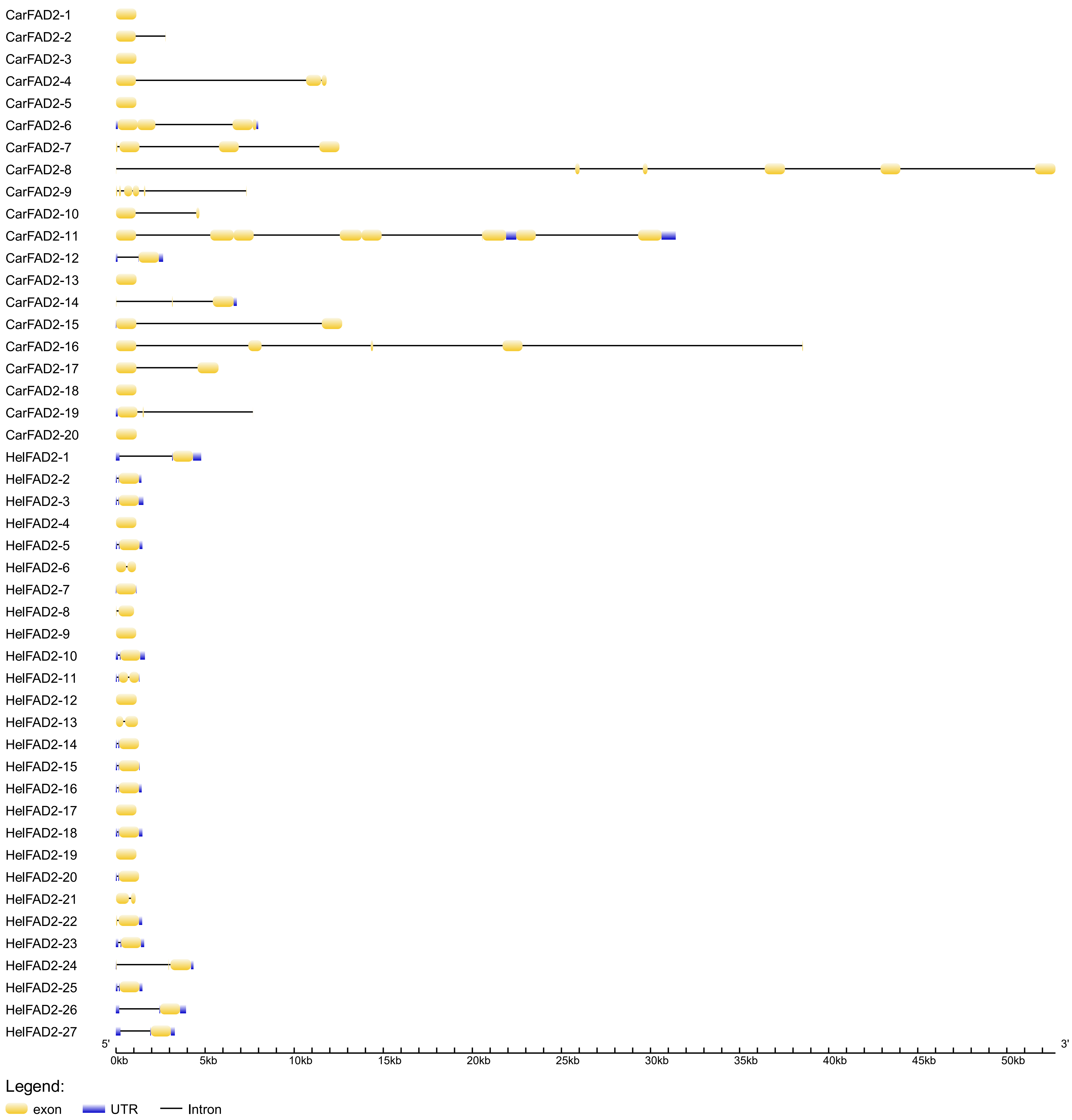

Fig. S48 Gene structure of *FAD2* members identified in safflower and sunflower.

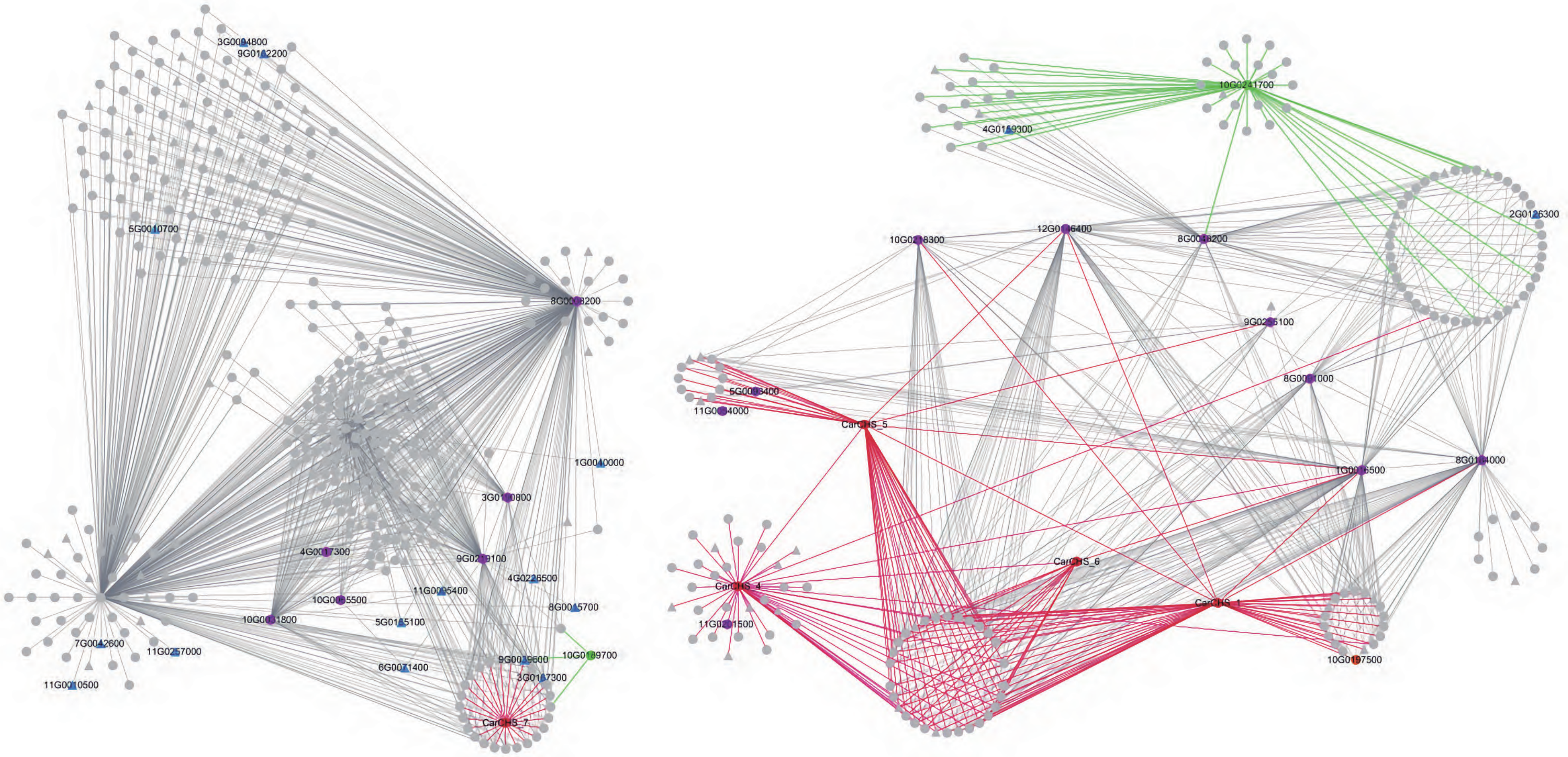

Fig. S49 *CarCHSs*-associated coexpression network in flower. Pink and green dots represent *UGTs* and *ASPs*, respectively; blue triangles represent transcriptional factors, *MYBs* and *bHLHs*.
