# Supplementary material for: The chromosome‐scale reference genome of safflower (Carthamus tinctorius) provides insights into linoleic acid and flavonoid biosynthesis
Source: Plant Biotechnol J. 2021 Apr 8;19(9):1725–42. doi: 10.1111/pbi.13586 (PMC8428823; doi:10.1111/pbi.13586)
Supplement: Supplementary file 3 — Table S2 Statistics of 12 chromosomes (superscaffolds) assembled by long reads assisted with Hi‐C sequencing Table S4 BUSCO results for the safflower genome Table S7 Genomes of the representative plant species used in the phylogenomic and comparative genomics analyses [file PBI-19-1725-s015.docx]

**Table S2.** **Assembled 12 chromosomes (superscaffolds) with Hi-C sequencing of safflower.** Length of contigs represents the total length of all contigs for each supercaffold, and length of superscaffold represents the total length of all contigs and 500 ‘N’ joining the adjacent contigs for these contigs.

| **Superscaffold** | **Number of contigs** | **Length of contigs** | **Length of superscaffolds** |
| --- | --- | --- | --- |
| superscaffold1 | 23 | 106,756,230 | 106,767,230 |
| superscaffold2 | 18 | 88,252,849 | 88,261,349 |
| superscaffold3 | 18 | 93,848,730 | 93,857,230 |
| superscaffold4 | 10 | 92,415,650 | 92,420,150 |
| superscaffold5 | 20 | 87,856,749 | 87,866,249 |
| superscaffold6 | 26 | 82,687,007 | 82,699,507 |
| superscaffold7 | 22 | 87,252,386 | 87,262,886 |
| superscaffold8 | 16 | 97,150,524 | 97,158,024 |
| superscaffold9 | 9 | 88,177,339 | 88,181,339 |
| superscaffold10 | 18 | 84,808,067 | 84,816,567 |
| superscaffold11 | 13 | 74,728,018 | 74,734,018 |
| superscaffold12 | 20 | 73,159,026 | 73,168,526 |
| Total | 213 | 1,057,092,575 | 1,057,193,075 |

**Table S4. Report of BUSCO results for the safflower genome.**

| C:90.7% [S:85.0%, D:5.7%], F:2.4%, M:6.9%, n:1440 | |
| --- | --- |
| 1306 | Complete BUSCOs (C) |
| 1224 | Complete and single-copy BUSCOs (S) |
| 82 | Complete and duplicated BUSCOs (D) |
| 35 | Fragmented BUSCOs (F) |
| 99 | Missing BUSCOs (M) |
| 1440 | Total BUSCO groups searched |

**Table S7.** Genomes of representative plant species used in phylogenomics and comparative genomics analyses.

| **Index** | **Common name**  **(Taxon)** | **Scientific name** | **Reference** |
| --- | --- | --- | --- |
| 1 | Arabidopsis  (Brassicaceae) | *Arabidopsis thaliana* | Arabidopsis Genome Initiative, 2000, Nature |
| 2 | Grape  (Vitaceae) | *Vitis vinifera* | Jaillon *et al.*,2007, Nature |
| 3 | Coffea  (Rubiaceae, Asterid I) | *Coffea canephora* | Denoeud *et al.*, 2014 Science |
| 4 | Sweet wormwood (Asteraceae,  Asterid II) | *Artemisia annua* | Shen *et al.*,2018, Molecular Plant |
| 5 | Juhuanao  (Asteraceae,  Asterid II) | *Chrysanthemum nankingense* | Song *et al.*,2018, Molecular Plant |
| 6 | Artichoke  (Asteraceae,  Asterid II) | *Cynara cardunculus* | Acquadro *et al.*,2017, Scientific Reports |
| 7 | Dengzhanhua  (Asteraceae,  Asterid II) | *Erigeron breviscapus* | Yang *et al.*,2017, Giga Science |
| 8 | Sunflower  (Asteraceae,  Asterid II) | *Helianthus annuus* | Badouin *et al.*, 2017, Nature |
| 9 | Lettuce  (Asteraceae,  Asterid II) | *Lactuca sativa* | Zhang *et al.*,2017, Nature Communications |
| 10 | Safflower  (Asteraceae,  Asterid II) | *Carthamus tinctorius* | The present study |
